# Gasdermin C promotes Stemness and Immune Evasion in Pancreatic Cancer via Pyroptosis‐Independent Mechanism

**DOI:** 10.1002/advs.202308990

**Published:** 2024-09-19

**Authors:** Renfei Wu, Jingwei Li, Alexandra Aicher, Ke Jiang, Serena Tondi, Shuang Dong, Quan Zheng, Siqi Tang, Minchun Chen, Zhenyang Guo, Berina Šabanović, Preeta Ananthanarayanan, Lingxi Jiang, Anna Sapino, Chenlei Wen, Da Fu, Baiyong Shen, Christopher Heeschen

**Affiliations:** ^1^ Center for Single‐Cell Omics School of Public Health Shanghai Jiao Tong University School of Medicine 227 South Chongqing Road Shanghai 200025 P. R. China; ^2^ State Key Laboratory of Systems Medicine for Cancer Shanghai Jiao Tong University School of Medicine 227 South Chongqing Road Shanghai 200025 P. R. China; ^3^ Department of General Surgery Pancreatic Disease Center Ruijin Hospital Shanghai Jiao Tong University School of Medicine 227 South Chongqing Road Shanghai 200025 P. R. China; ^4^ Research Institute of Pancreatic Diseases Shanghai Key Laboratory of Translational Research for Pancreatic Neoplasms Shanghai Jiaotong University School of Medicine 227 South Chongqing Road Shanghai 200025 P. R. China; ^5^ Precision Immunotherapy Graduate Institute of Biomedical Sciences China Medical University No. 91, Xueshi Road Taichung 404 Taiwan; ^6^ Immunology Research and Development Center China Medical University No. 91, Xueshi Road Taichung 404 Taiwan; ^7^ Pancreatic Cancer Heterogeneity Candiolo Cancer Institute – FPO – IRCCS Strada Provinciale 142 Km 3,95 Candiolo (TO) 10060 Italy; ^8^ School of Pharmacy East China University of Science and Technology 130 Meilong Road Shanghai 200237 P. R. China; ^9^ Department of Pathology Candiolo Cancer Institute – FPO – IRCCS Strada Provinciale 142 Km 3,95 Candiolo (TO) 10060 Italy

**Keywords:** cancer stem cells, gasdermin C, immune evasion, invasion, immunotherapy, KRAS inhibition, metastasis, pancreatic ductal adenocarcinoma

## Abstract

Pancreatic ductal adenocarcinoma (PDAC) is a highly metastatic and lethal disease. Gasdermins are primarily associated with necrosis via membrane permeabilization and pyroptosis, a lytic pro‐inflammatory type of cell death. In this study, GSDMC upregulation during PDAC progression is reported. GSDMC directly induces genes related to stemness, EMT, and immune evasion. Targeting Gsdmc in murine PDAC models reprograms the immunosuppressive tumor microenvironment, rescuing the recruitment of anti‐tumor immune cells through CXCL9. This not only results in diminished tumor initiation, growth and metastasis, but also enhances the response to KRAS^G12D^ inhibition and PD‐1 checkpoint blockade, respectively. Mechanistically, it is discovered that ADAM17 cleaves GSDMC, releasing nuclear fragments binding to promoter regions of stemness, metastasis, and immune evasion‐related genes. Pharmacological inhibition of GSDMC cleavage or prevention of its nuclear translocation is equally effective in suppressing GSDMC's downstream targets and inhibiting PDAC progression. The findings establish GSDMC as a potential therapeutic target for enhancing treatment response in this deadly disease.

## Graphical abstract

This study unveils Gasdermin C (GSDMC) as a transcriptional master regulator of stemness and immune evasion in pancreatic ductal adenocarcinoma, acting downstream of EMT‐inducing factors. Mechanistically, GSDMC blocks the T cell‐recruiting chemokine CXCL9 and directly upregulates stemness and immune checkpoing molecules. Genetic or pharmacological targeting of GSDMC might be useful for enhancing response to immunotherapy in this still deadly disease.

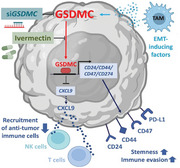

## 1 Introduction

Pancreatic cancer, including pancreatic ductal adenocarcinoma (PDAC) as its most common form, is an extremely deadly disease characterized by extensive metastasis.^[^ ^1^, ^2^, ^3^ ^]^ Current treatments for PDAC provide hardly any long‐term survival benefits,^[^ ^4^, ^5^, ^6^ ^]^ and by 2030, PDAC may even become the second leading cause of cancer‐related deaths.^[^ ^7^ ^]^ Compelling evidence, from our laboratory and others, suggests that PDAC harbors cancer cells with stemness features that bear the unique ability, even at the single‐cell level, to initiate new tumors and metastases. Due to their functional reminiscence to normal stem cells, they have been termed cancer stem cells (CSCs). As CSCs play a key role in metastasis, drug resistance, and relapse of the disease,^[^ ^8^, ^9^, ^10^ ^]^ we need to develop novel treatment strategies that also efficiently target CSCs. For this purpose, it is imperative to gain a thorough understanding of the regulatory mechanisms governing the aggressive phenotypes of CSCs.

In this context, epithelial‐mesenchymal transition (EMT) is a cellular process that is critical for invasion and metastasis of cancer. To date, various EMT‐related genes have been identified, and some of them, such as ZEB1/2, reportedly contribute to the aggressive behavior of PDAC cells.^[^ ^11^ ^]^ To identify novel downstream regulators of invasive pancreatic cancer (stem) cells, we performed an exploratory single‐cell RNA sequencing analysis using a representative set of primary human PDAC models. Our data revealed that invasive PDAC cells consistently overexpress Gasdermin C (*GSDMC*). Gasdermins are a family of pore‐forming proteins and are mainly expressed in the gastrointestinal tract, skin, and immune cells. They play a decisive role in pyrolytic cell death or pyroptosis, an inflammatory cell death that is commonly related to microbial infections. Five of the six members of the gasdermin family have been linked to important biological functions in inflammatory diseases, such as asthma and sepsis. Under pyroptosis‐inducing conditions, gasdermins are cleaved and release the pore‐forming N‐terminal fragment, allowing it to insert into cell membranes and form large oligomeric pores that disrupt ion homeostasis and ultimately lead to pyroptotic cell death.^[^ ^12^, ^13^ ^]^

Hence, initially, it was difficult to reconcile why PDAC cells would overexpress gasdermins, leading to their own cell death. However, recent studies suggested that in PDAC, gasdermins may play a tumor‐promoting role through mechanisms that are distinct from their known pore‐forming functions, without any demonstrated role in the nucleus. For example, *GSDME* is strongly expressed in PDAC and mediates resistance to pancreatic enzymatic digestion through the GSDME–YBX1–Mucin pathway.^[^ ^14^ ^]^ Specifically, GSDMC comprises the cytotoxic N‐terminal functional domain, but also contains a C‐terminal regulatory domain. These are connected by a linker containing a specific cleavage site. However, our understanding of the compartmentalization for GSDMC and the specific enzymes responsible for cleaving GSDMC into its N‐terminal and C‐terminal fragments was still scarce.^[^ ^12^, ^15^ ^]^ In the present study, we now demonstrate that, upon activation, the C‐terminal domain is cleaved by proteolytic enzymes, such as ADAM17, leading to the release of the C‐terminal domain. Subsequently, the C‐terminal domain is transported from the Golgi to the nucleus, resulting in the production of nuclear GSDMC. Notably, the C‐terminal fragment is exclusively responsible for binding to the relevant promoter regions and mediating the effects of GSDMC in PDAC.

Our findings demonstrate a cell context‐dependent role of *GSDMC* in promoting pancreatic cancer. Genetic intervention targeting *GSDMC* resulted in a significant reduction in the invasion and metastasis of pancreatic cancer cells, highlighting the critical role of GSDMC in the dissemination process of PDAC. In terms of its mechanism, GSDMC functions as a regulator of gene expression and is involved in promoting the expression of critical components of immune evasion and stemness, including CD47, CD24, CD44, and PD‐L1. Targeting GSDMC may prime the tumor microenvironment for subsequent immunotherapies, such as PD‐L1/PD‐1 checkpoint inhibition, offering a potentially more effective strategy for treating this fatal disease. These findings suggest that GSDMC could be a novel therapeutic target for combating pancreatic cancer progression.

## 2 Results

### 2.1 GSDMC is Upregulated Upon Induction of EMT in PDAC Cells

**Figure 1:**
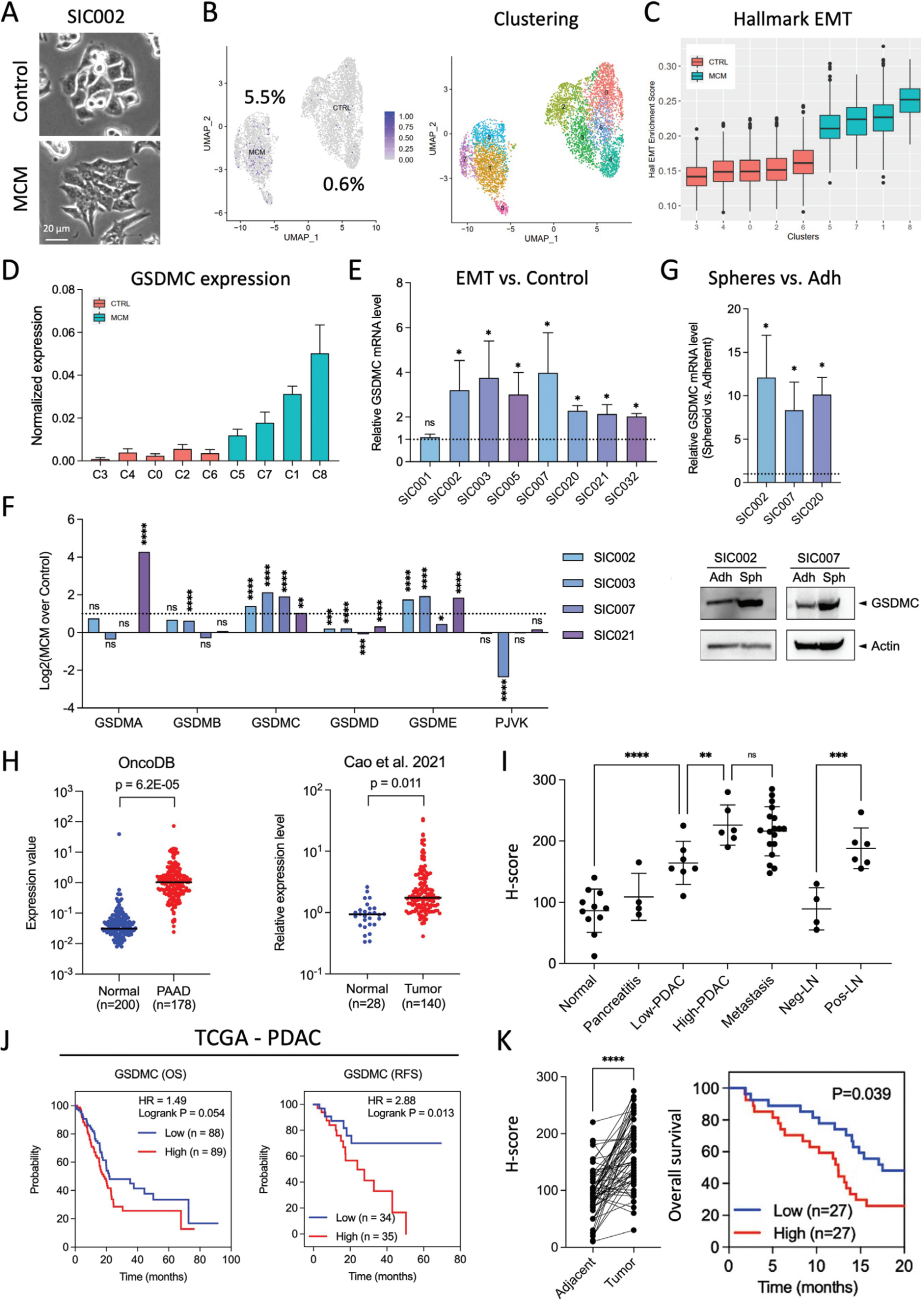
Gasdermin C is overexpressed in advanced PDAC. A) Representative morphology of primary human PDAC culture SIC002 following treatment with macrophage‐conditioned medium (MCM) or control medium. B) UMAP projection of single‐cell data demonstrating the percentage of GSDMC^+^ cancer cells within primary PDAC cultures (SIC002) after pre‐treatment with EMT‐inducing MCM or control medium (left panel). UMAP projection specifically highlighting clusters 0–8 (right panel). C) Hallmark EMT enrichment score for clusters 0–8 in PDAC cultures treated with MCM or control medium. D) Expression levels of *GSDMC* in clusters 0–8 from the UMAP projection shown in (C). E) The qPCR fold change of *GSDMC* expression in eight different human primary PDAC cell cultures after 48 hours of exposure to EMT‐inducing MCM pre‐treatment. The dotted line represents the fold change in expression without MCM treatment (n = 3 independent samples). F) The single‐cell RNA‐seq data illustrates the expression of *GSDMA*, *GSDMB*, *GSDMC*, *GSDMD*, *GSDME*, and *PJVK* in four different primary PDAC cultures. The dotted line indicates Log_2_2 = 1, which signifies a threshold of 2 (t‐test). G) qPCR fold change of *GSDMC* expression in CSC‐enriched spheres derived from three human primary PDAC cell cultures (upper panel). Fold change values are compared to expression levels in adherent cells (Adh), indicated by the dotted line. Protein levels of GSDMC in spheres versus adherent cells, detected by Western blot, are shown (lower panel) (n = 3 independent samples). Full‐length GSDMC was detected by the GSDMC antibody BS‐16332R (Bioss). H) *GSDMC* gene expression profiles in PDAC tumor tissue compared to normal tissue from two different datasets (left panel: OncoDB (https://oncodb.org), right panel: Cao et al.,^[^ ^49^ ^]^ I) H‐score analysis of GSDMC staining in histological specimens from PDAC patients, including low‐grade well‐differentiated PDAC (Low‐PDAC, n = 7), high‐grade poorly differentiated PDAC (High‐PDAC, n = 6), metastatic PDAC specimens (n = 18), PDAC tumor‐positive lymph nodes (Pos‐LN, n = 6), negative lymph nodes (Neg‐LN, n = 4), and pancreatitis specimens (n = 4). One‐way ANOVA with Fisher's LSD test was used for statistical analysis. J) Prognostic value of *GSDMC* mRNA expression in PDAC patients from the TCGA data base for overall survival (OS) and relapse‐free survival (RFS) as analyzed by Kaplan‐Meier Plotter (https://kmplot.com/analysis/).^[^ ^50^ ^]^ Patient samples were dichotomized by the median value of the target gene. K) H‐score analysis of staining for GSDMC protein in histological specimens from a different set of PDAC patients enrolled at Ruijin Hospital (Shanghai, China), comparing adjacent tumor‐free tissue with tumor tissue (left panel) (n = 60). Prognostic value of GSDMC expression derived from PDAC patients for overall survival (OS) (right panel) (n = 54) using the log‐rank test (right panel). *p < 0.05, **p < 0.01, ***p < 0.001, ****p < 0.0001; Mann Whitney test, two‐tailed. Please also see Figure S1 (Supporting Information).

To identify genes that may play an important role in invasive PDAC cells, we first produced single‐cell RNA‐seq data from a representative set of primary human PDAC cultures derived from circulating tumor cells (CTCs; SIC002, SIC003, SIC007) and resected primary tumors (SIC021) in the presence or absence of EMT‐inducing macrophage‐conditioned medium (MCM). EMT induction was validated by morphological changes of the cells (**Figure** **1**A; Figure S1A, Supporting Information) and upregulation of the EMT‐related genes *ZEB1/2, SNAIL, SLUG, VIM, LOXL2*, and *SOX4* (Figure S1B, Supporting Information). We previously showed that oncostatin M (OSM), derived from tumor‐associated macrophages, acts as a major inducer of the upregulation of EMT‐related genes, including *LOXL2*.^[^ ^16^ ^]^ Indeed, we found that OSM was equally potent as MCM for inducing EMT and upregulating *GSDMC* (Figure S1C, Supporting Information). UMAP analysis comparing the MCM‐treated and control populations in human primary PDAC models showed a notable difference in *GSDMC* expression, with higher levels detected in the MCM‐treated population (Figure 1B). This unexpected discovery sparked our interest in investigating the potential implications of GSDMC differential expression in the context of EMT induction and its potential significance for PDAC invasiveness. Using EMT hallmark signatures, we confirmed the specific upregulation of *GSDMC* in PDAC cells that responded to above EMT cues (Figure 1C,D; Figure S1D,E, Supporting Information). Subsequently, qPCR analysis of eight different human primary PDAC cell cultures exposed to EMT‐inducing MCM corroborated these results showing a significant increase in expression of *GSDMC* for seven models (Figure 1E). Moreover, validation of GSDMC expression at the protein level was performed on three PDAC models, further substantiating the qPCR results (Figure S1F, Supporting Information). When checking the modulation of other members of the gasdermin family in the context of EMT, we found that *GSDME* was the only other family member apart from *GSDMC* that was highly significantly upregulated in three out of the four PDAC models tested (Figure 1F). This gasdermin family member was previously studied in the context of PDAC revealing that GSDME mediates resistance of PDAC to enzymatic digestion via the YBX1‐mucin pathway.^[^ ^14^ ^]^

To identify additional pathways that may be altered alongside EMT, GSEA plots were generated for GSDMC^+^ versus GSDMC^–^ cells treated with MCM (Figure S1G, Supporting Information). In addition to EMT‐related pathways, several other pathways including immune response, ER‐Golgi transport as well as cellular dynamics and metabolism were enriched in GSDMC^+^ cells. Consistently, GSDMC was strongly upregulated both at mRNA and protein levels in CSC‐enriched spheres compared to their more differentiated progenies in adherent cultures (Figure 1G; Figure S1H, Supporting Information), suggesting a potential role in stemness. Notably, none of the other gasdermins showed a similarly prominent and consistent expression pattern (Figure S1I, Supporting Information). Therefore, we focused on GSDMC for our subsequent experiments.

### 2.2 GSDMC is Overexpressed in PDAC Tumors and Associated with Poor Survival

To further support our hypothesis that GSDMC plays an important functional role in PDAC progression, we explored the expression of *GSDMC* in PDAC tumors versus normal tissue using two different datasets. Our results showed that GSDMC was significantly and consistently upregulated in PDAC tumors compared to normal tissue (Figure 1H). Additionally, we performed immunohistochemistry (IHC) to investigate GSDMC expression in various pancreatic tissues using a TMA with normal pancreas, pancreatitis, primary PDAC lesions, metastases, and lymph nodes (Figure 1I; Figure S1J, Supporting Information J). H‐score analysis showed that GSDMC is upregulated in low‐grade PDAC, which is further enhanced in high‐grade PDAC and metastasis. Similarly strong GSDMC expression can be detected in cancer‐positive lymph nodes, but not in cancer‐negative lymph nodes. These findings could be validated in a larger set of paired PDAC versus adjacent tissues (Figure S1K, Supporting Information) as well as a different cohort of 14 PDAC patients, demonstrating that GSDMC is indeed consistently overexpressed in PDAC tissue (Figure S1L, Supporting Information). In addition, GSDMC expression patterns were examined in KPC mice that display lesions ranging from pancreatic intraepithelial neoplasia (PanIN) toward fully developed PDAC. Interestingly, we observed a progressive increase in nuclear GSDMC staining during the transformation process in the pancreas, suggesting a potential involvement of GSDMC in the progression of the disease (Figure S1M, Supporting Information).

To investigate the potential prognostic impact of GSDMC overexpression in PDAC, we next performed survival analyses. Results from the TCGA datasets show that, in PDAC, high expression of *GSDMC* tends to negatively correlate with overall survival (OS) and is significantly linked to a decrease in recurrence‐free survival (RFS) (Figure 1J). Interestingly, none of the other gasdermin family members displayed a significant correlation with OS or RFS for PDAC (Figure S1N, Supporting Information), whereas GSDMC expression was also correlated with outcome for several other cancers (Figure S1O, Supporting Information). Utilizing the H‐score of our own set of clinical PDAC specimens (n = 54) to dichotomize patients for GSDMC expression, we could further validate a significant association between GSDMC protein levels and adverse outcome in PDAC (Figure 1K).

### 2.3 GSDMC Regulates Tumor‐Initiation and Metastasis in PDAC

**Figure 2:**
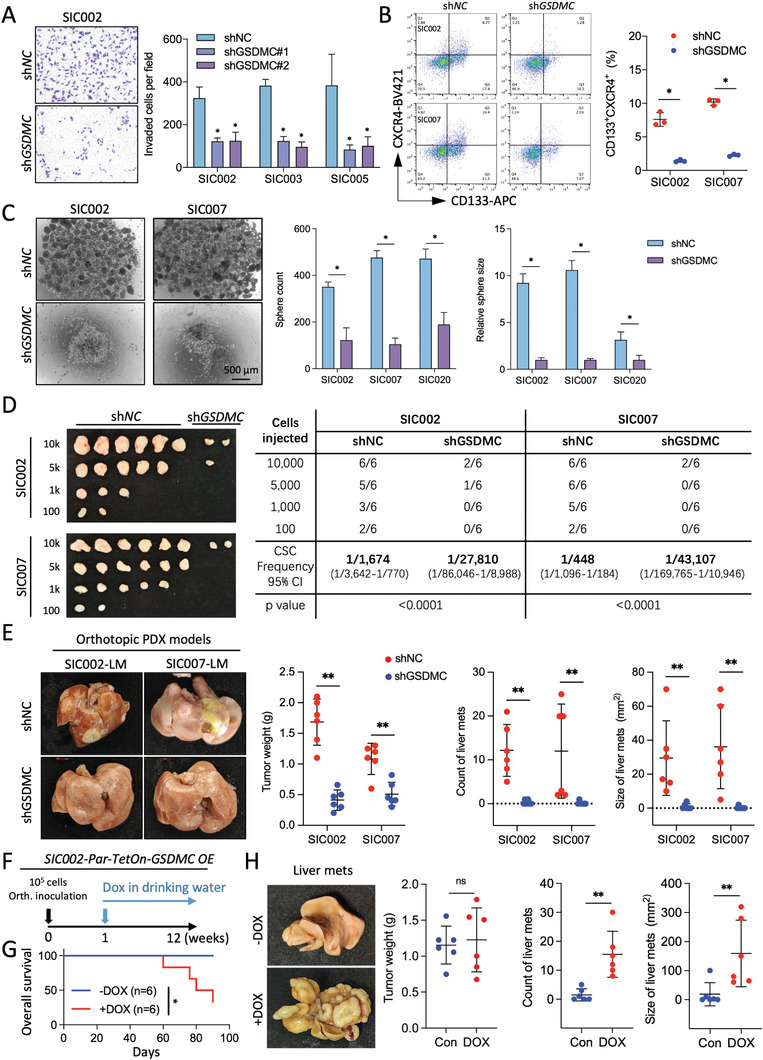
GSDMC is linked to invasion and stemness phenotypes. A) Invasion assay was performed in three different human primary PDAC cultures after knockdown of GSDMC using two different sh*GSDMCs* compared to sh*NC* control. Representative images show invaded cells stained with crystal violet (left panel), and quantification of invaded cells is shown on the right panel (n = 3 independent samples). B) Flow cytometry analysis for the content of CD133^+^ CXCR4^+^ cancer stem cells (CSCs) in two different human primary PDAC cultures following genetic targeting of *GSDMC*. Representative flow cytometric analyses (left), quantification (right) (n = 3 independent samples). C) Sphere formation capacity expressed as number of formed spheres per 10,000 cells in 1 mL, following knockdown of *GSDMC* in three different human primary PDAC cultures. Representative photographs (left), quantification of sphere counts (middle), and sphere size (right) (n = 3 independent samples). D) In vivo tumorigenicity of decreasing numbers of two different primary PDAC cultures following genetic targeting of *GSDMC*. N = 6 for both groups. Gross morphology of the explanted tumors (left), tumor take rate and CSC frequency (right). E) Liver metastases (LM) developed in an orthotopic PDAC model after intrapancreatic injection of human primary PDAC cultures with knockdown of *GSDMC* (sh*GSDMC*) or control (sh*NC*). Representative photographs of macroscopic liver metastases (left), assessment of liver metastases (right), and weight of the primary pancreatic tumors (middle). N = 6 for both groups. F) Schematic of the experimental design. G) Kaplan Meier survival analysis of mice following orthotopic injection of human primary PDAC cells into the pancreas followed by doxycycline (DOX)‐inducible overexpression (OE) of *GSDMC*. N = 6 for both groups. Statistical analysis was performed using the log‐rank test. H) Macroscopic liver metastases (LM) developed in (G) following doxycycline (DOX)‐induced *GSDMC* overexpression or control (Con). Representative photographs of macroscopic liver metastases (left), assessment of liver metastases (right), and weight of the primary pancreatic tumors (middle). *p < 0.05 and **p < 0.01; Mann–Whitney U test, two‐tailed, unless stated otherwise. Please also see Figure S2 (Supporting Information).

Immunohistochemistry of tumor specimens shows enhanced GSDMC protein expression for invading vimentin^+^ PDAC cells in vivo (Figure S2A, Supporting Information) and ex vivo spheroids (Figure S2B, Supporting Information). Consistently, immunofluorescence revealed enhanced GSDMC expression for the migrating front in scratch wound assays (Figure S2C, Supporting Information). These data further supported our hypothesis that GSDMC could be an important regulator of invasiveness in PDAC. Therefore, we knocked down GSDMC in four different human primary PDAC cultures and validated the strong knockdown efficiency at the mRNA (Figure S2D, Supporting Information) and protein (Figure S2E, Supporting Information) levels. Notably, inhibition of GSDMC did not alter the proliferative capacity of the cells in vitro (Figure S2F, Supporting Information) and in vivo (Figure S2G, Supporting Information) but abolished the MCM‐induced morphological changes (Figure S2H, Supporting Information). As such, our data imply that the observed reduced invasion of sh*GSDMC* PDAC cells can be solely attributed to their diminished invasive capacity both in human (**Figure** **2**A) and murine PDAC cells (Figure S2I–K, Supporting Information).

RNAseq analysis of sh*GSDMC* versus sh*NC* PDAC cells demonstrated downregulation of various genes that were not only restricted to EMT, but also related to stemness and cell survival (Figure S2L–N, Supporting Information). These data could be validated by qPCR in murine (Figure S2O, Supporting Information) and human PDAC cells (Figure S2P, Supporting Information). Consistently, flow cytometry confirmed that targeting GSDMC also led to a decrease in the content of CSCs detected as ALDH fluor^+^ (Figure S2Q, Supporting Information), CD133^+^CD44^+^ (Figure S2R, Supporting Information) or CD133^+^CXCR4^+^ (Figure 2B) cells.^[^ ^8^ ^]^ These changes translated into a significantly reduced sphere formation capacity for human and murine PDAC cells (Figure 2C; Figure S2S, Supporting Information).

For the limiting dilution assay (LDA) to assess in vivo tumorigenicity, we subcutaneously injected diminishing numbers of primary human PDAC cells with or without sh*GSDMC* into nude mice. These experiments revealed consistently reduced in vivo tumorigenicity for sh*GSDMC* cells compared to sh*NC* PDAC cells (Figure 2D), despite their similar proliferative capacity (Figure S2G, Supporting Information). Next, to study the metastatic capacity of human PDAC cells in the absence or presence of *GSDMC* knockdown, we orthotopically implanted a relatively large number (1 × 10^5^) of highly metastatic PDAC cells, which were previously harvested from liver metastases (SIC002‐LM and SIC007‐LM). Upon injection of these PDAC cells with sh*NC* versus sh*GSDMC*, consistent tumor formation was observed for both groups, although the tumor weight was reduced in the sh*GSDMC* group (Figure 2E). Mice receiving sh*NC* cells exhibited the expected high incidence of liver metastases, while no liver metastases were detected in mice injected with sh*GSDMC* cells (Figures 2E; Figure S2T, Supporting Information).

To directly study the impact of GSDMC on the in vivo metastatic capacity of PDAC cells, we instead injected the corresponding parental PDAC cells that bear very low metastatic capacity (SIC002‐Par) into the pancreas. These cells were lentivirally transduced with a construct for DOX‐inducible overexpression of *GSDMC* (Figure S2U, Supporting Information). Notably, neither forced overexpression of *GSDMC*, treatment with TNFα as a common inducer of pyroptosis,^[^ ^17^ ^]^ or a combination of both, induced pyroptosis in PDAC cells (Figure S2V, Supporting Information). For the in vivo experiment, *GSDMC* overexpression was induced by DOX administration one week after implantation of the PDAC cells (Figure 2F). While tumor formation was comparable for both groups, we still found abundant liver metastases in *GSDMC* overexpressing tumors (DOX) compared to no detectable metastases for the control group (Figure 2H). This translated into diminished overall survival for the DOX group (Figure 2G). Together, these results conclusively demonstrate that GSDMC regulates stemness/tumorigenicity and invasion/metastasis in PDAC.

### 2.4 In Vivo Phenotype of GSDMC Effects is Related to Enhanced Immune Evasion

**Figure 3:**
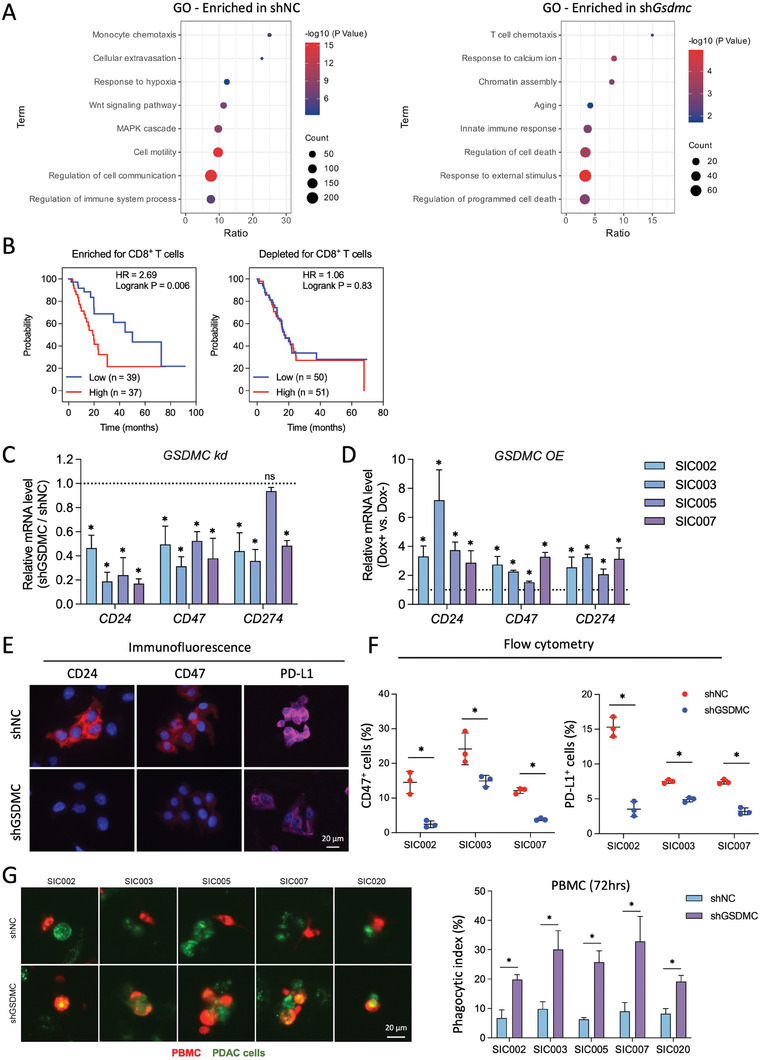
GSDMC expression is linked to immune evasion signals. A) Enriched Gene Ontology (GO) pathway analysis in PDAC primary cultures with *GSDMC* knockdown (sh*GSDMC*) compared to control (sh*NC*). B) Prognostic value of GSDMC expression in PDAC patients for overall survival (OS) using datasets enriched or depleted for CD8^+^ T cells, analyzed through Kaplan‐Meier Plotter. C) qPCR analysis of RNA levels for the immune evasion molecules *CD24*, *CD47*, and *CD274* (encoding PD‐L1) following knockdown (*kd*) of *GSDMC* in four different PDAC cultures. The dotted line indicates control treatment (sh*NC*) (n = 3 independent samples). D) qPCR analysis of RNA levels for the immune evasion molecules *CD24*, *CD47*, and *CD274* following DOX‐inducible overexpression (*OE*) of *GSDMC* (n = 3 independent samples). E) Immunofluorescence for CD24, CD47, and PD‐L1 expression (red) after *GSDMC* knockdown (sh*GSDMC*) versus control (sh*NC*). Nuclei were stained with DAPI (blue). F) Flow cytometry analysis for content of CD47^+^ and PD‐L1^+^ cells following genetic targeting of *GSDMC* (sh*GSDMC*: blue) versus control treatment (sh*NC*: red) (n = 3 independent samples). G) Phagocytic capacity of PBMCs (cell tracker, red) in the presence of PDAC cultures (expressing GFP, green) with genetic targeting of *GSDMC* (sh*GSDMC*) versus control (sh*NC*). Representative immunofluorescence (left), quantification of the phagocytic index (right) (n = 3 independent samples). *p < 0.05; Mann–Whitney U test, two‐tailed. Please also see Figure S3 (Supporting Information).

In a first attempt to further our knowledge about the downstream mechanism of action for GSDMC in PDAC, we performed RNAseq for sh*NC* versus sh*Gsdmc* PDAC cells. The Kyoto Encyclopedia of Genes and Genomes (KEGG) pathway analysis highlighted stronger expression of oncogenic cascades such as PI3K‐Akt, Ras, and Jak‐STAT signaling in shNC cells, while the tumor‐suppressing P53 pathway was enriched following *Gsdmc* knockdown (Figure S3A, Supporting Information). Interestingly, Gene Ontology (GO) enrichment analysis showed T cell chemotaxis as most prominently upregulated upon *GSDMC* knockdown, whereas monocyte chemotaxis was enriched in sh*NC* PDAC cells (**Figure** **3**A).

These data suggested that *GSDMC* might also have immune modulatory functions in PDAC. To explore this hypothesis, we conducted further subgroup analyses of the patient survival data presented in Figure 1J. We stratified the data according to intratumoral content of CD8^+^ T cells. Interestingly, patients with enriched CD8^+^ T cells showed a much more pronounced difference in outcome for GSDMC positive versus negative patients whereas patients with tumors depleted of CD8^+^ T cells showed virtually no difference (Figure 3B).

To further explore this hypothesis, we next analyzed the expression of several key checkpoint molecules in human PDAC cells and found that *CD24*, *CD47* and PD‐L1 (*CD274*) were significantly downregulated following GSDMC knockdown (Figure 3C) and upregulated upon DOX‐inducible GSDMC overexpression (Figure 3D). These data could be validated at the protein level using immunofluorescence (Figure 3E) and flow cytometry (Figure 3F). Therefore, we next assessed the phagocytic capacity of human peripheral blood mononuclear cells (PBMCs) co‐cultured with PDAC cells in the absence or presence of genetic targeting of GSDMC. We found that phagocytosis was significantly increased by knockdown of GSDMC (Figure 3G). Consistent results were obtained using immortalized murine Bone Marrow‐Derived Macrophages (iBMDM) and THP‐1‐derived macrophages (Figure S3B,C, Supporting Information). In addition, we investigated the functional relevance of the modulation of the “Don't eat me signal” CD47 by GSDMC. Together, these results suggest a possible association between GSDMC expression and the immunosuppressive tumor microenvironment in PDAC.

To conclusively validate the hypothesis that GSDMC promotes in vivo progression of PDAC by modulating the immunosuppressive tumor microenvironment, we utilized a murine PDAC model based on PDAC cells derived from KPC mice (LSL‐Kras^G12D/+^; LSL‐Trp53^R172H/+^; Pdx‐1‐Cre) combined with genetic targeting of murine *Gsdmc* (sh*Gsdmc*) versus no targeting (sh*NC*). First, we confirmed that our observations in human PDAC cells also apply to murine PDAC cells. Indeed, knockdown of *Gsdmc* also resulted in a robust down‐regulation of genes related to stemness and immune evasion (Figure S4A, Supporting Information), while in vitro cell proliferation remained unchanged (Figure S4B, Supporting Information). Co‐culture of murine PDAC cells with iBMDM cells resulted in diminished confluency upon *Gsdmc* knockdown (Figure S4C, Supporting Information), which could be attributed to enhanced phagocytosis upon downregulation of the “don't eat me” signal CD47. Indeed, the enhanced phagocytosis in the presence of *Gsdmc* knockdown could be rescued by neutralizing antibodies against CD47 (Figure S4D, Supporting Information). This finding highlights the critical role of CD47 modulation by Gsdmc in mediating the observed enhancement in phagocytosis.

**Figure 4:**
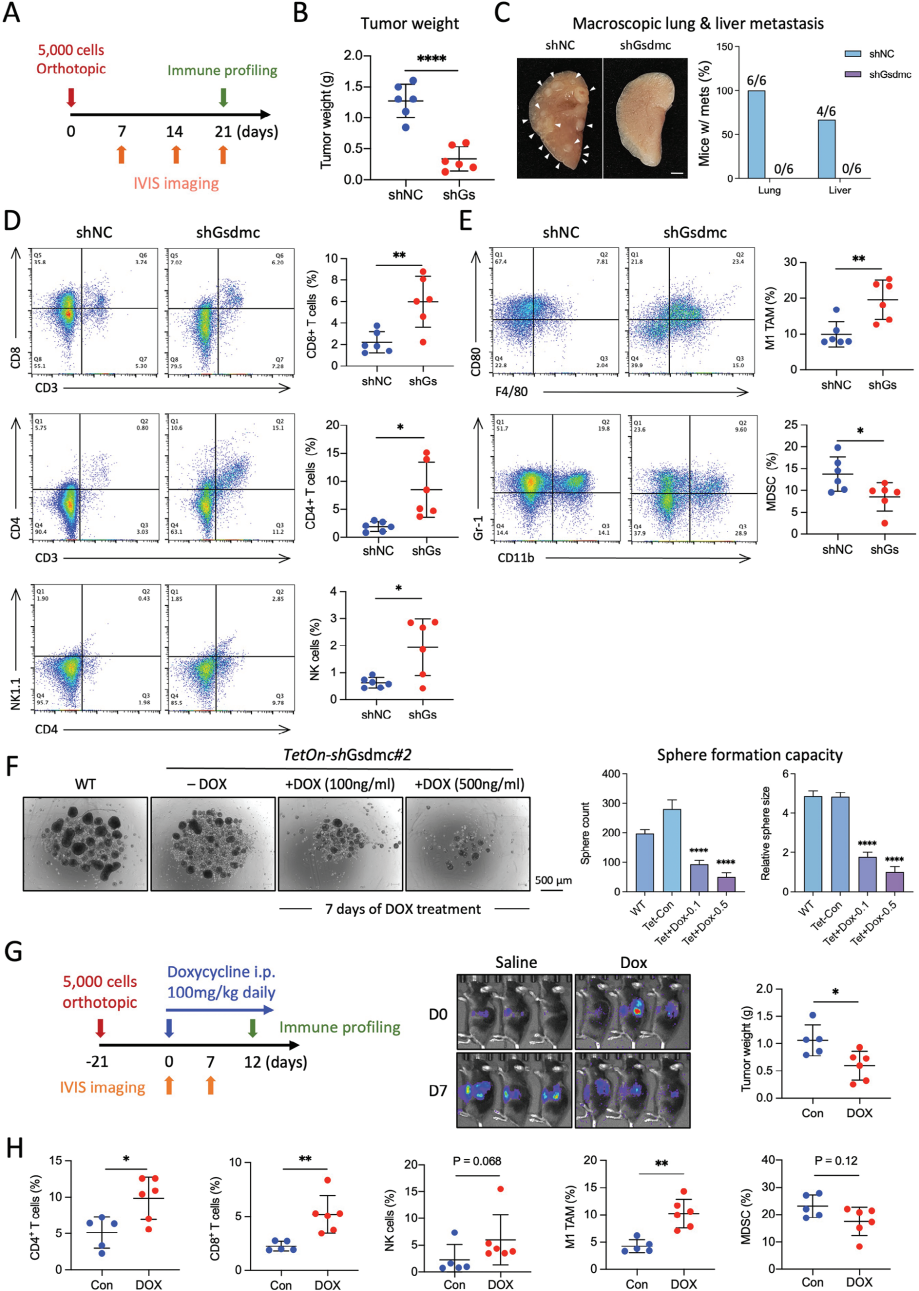
Gsdmc promotes immune evasion in PDAC. A) Schematic illustration of the treatment schedule. B) Tumor weight of primary pancreatic tumors in orthotopic syngeneic KPC models (CHX2000) with genetic targeting of murine *Gsdmc* (sh*Gsdmc*) versus control (sh*NC*). N = 6 for both groups (two‐tailed t‐test). C) Macroscopic lung metastases (left), quantification of liver and lung metastases (right). D) Immune profiling of sh*Gsdmc* tumors compared to control (sh*NC*) tumors. N = 6 for both groups. Representative flow cytometry dot plots showing CD8^+^ T cells, CD4^+^ T cells, and NK cells (left panel), with quantification in the right panel. The indicated percentage represents the proportion of live cells. E) Representative flow cytometry dot plots illustrating M1 TAM and MDSCs (left panel), with quantification in the right panel. The indicated percentage represents the proportion of live cells. N = 6 for both groups (two‐tailed t‐test). F) Sphere formation capacity of CHX2000 KPC cells expressed as number of formed spheres per 10,000 cells in 1 mL for DOX‐induced sh*Gsdmc* for 7 days versus no Dox (control) and WT KPC cells. Representative bright field images of sphere cultures (left panel) and quantification of sphere counts and size (right panel) (n = 3 independent samples). One‐way ANOVA analysis with Tukey's post‐hoc test comparing Tet‐DOX versus Tet‐Con. G) Schematic illustration of the treatment schedule of in vivo DOX‐induced *Gsdmc* knockdown in mice with orthotopic CHX2000 KPC tumors (left panel). Representative IVIS images for baseline and day 7 (middle panel). Tumor weight on day 12 (right panel). H) Immune cell content in the tumors on day 12 as assessed by flow cytometry. N = 5 for Control group, N = 6 for DOX group. *p < 0.05, **p < 0.01, and ****p < 0.0001; Mann–Whitney U test, two‐tailed, unless stated otherwise. Please also see Figure S4 (Supporting Information).

Next, we orthotopically implanted sh*Gsdmc* versus sh*NC* PDAC cells to create syngeneic and fully immunocompetent murine PDAC models (**Figure** **4**A). The weight of the forming primary pancreatic tumors was significantly decreased for the sh*Gsdmc* group (Figure 4B), despite similar proliferation rates for sh*Gsdmc* PDAC cells compared to sh*NC* PDAC cells in vivo (Figure S4E, Supporting Information). Macroscopic lung metastases (Figure 4C; Figure S4F, Supporting Information) were completely abolished in the sh*Gsdmc* group. To directly validate the diminished metastatic potential of sh*Gsdmc* PDAC cells, we next employed intrasplenic injections (Figure S4G, Supporting Information). We observed a significant reduction in total counts and sizes of liver metastases, underscoring the pivotal role of *Gsdmc* in facilitating the metastatic dissemination of PDAC cells.

The reduced tumor growth in the sh*Gsdmc* group, despite similar proliferation rates compared to sh*NC* PDAC cells, suggested that other mechanisms such as immune evasion may also contribute to this phenotype. Indeed, immune profiling of sh*Gsdmc* versus sh*NC* tumors by flow cytometry demonstrated a strong influx of cytotoxic T cells upon *Gsdmc* knockdown, while immunosuppressive cells were significantly decreased in sh*Gsdmc* mice. Specifically, anti‐tumor immune cells, such as cytotoxic CD8^+^ T cells, T‐helper CD4^+^ cells, NK cells, and M1 TAMs increased, whereas immunosuppressive cells, such as MDSCs decreased (Figure 4D,E). Immunohistochemistry revealed consistent results for sh*Gsdmc* versus sh*NC* mice (Figure S4H, Supporting Information). Notably, in patient samples GSDMC expression positively correlated with the infiltration of M2‐type macrophages, but inversely correlated with the influx of CD8^+^ T cells (Figure S4I, Supporting Information). Intriguingly, while PD‐1 immune checkpoint blockade alone expectedly did not improve overall survival of this aggressive PDAC model, the addition of sh*Gsdmc* resulted in superior outcome, leading to the emergence of several long‐term survivors (Figure S4J, Supporting Information). In line with these findings, GSDMC^+^ PDAC cells were predominantly expressing high levels of PD‐L1 as evidenced by immunofluorescent staining of shNC PDAC tissue (Figure S4K, Supporting Information).

To further consolidate above data, we also used an inducible knockdown system (*TetOn‐shGsdmc*) to efficiently target *Gsdmc* in fully established tumors. For in vitro validation of the inducible knockdown system, *TetOn‐shGsdmc* PDAC cells were treated with increasing doses of doxycycline, which resulted in a significant reduction in *Gsdmc* expression (Figure S4L, Supporting Information) and functionally translated into the expected diminished sphere formation capacity of sh*Gsdmc* PDAC cells compared to sh*NC cells* (Figure 4F). Following in vivo implantation of the cells and formation of tumors on day 21 (Figure 4G), the induction of sh*Gsdmc* resulted in a remarkable remodeling of the immune cell composition within the tumor microenvironment (Figure 4H) and subsequent inhibition of tumor progression (Figure 4G). Above data establish a prominent role for GSDMC in regulating both stemness and immune evasion in PDAC.

### 2.5 Nuclear GSDMC Transcriptionally Drives Aggressiveness of PDAC

**Figure 5:**
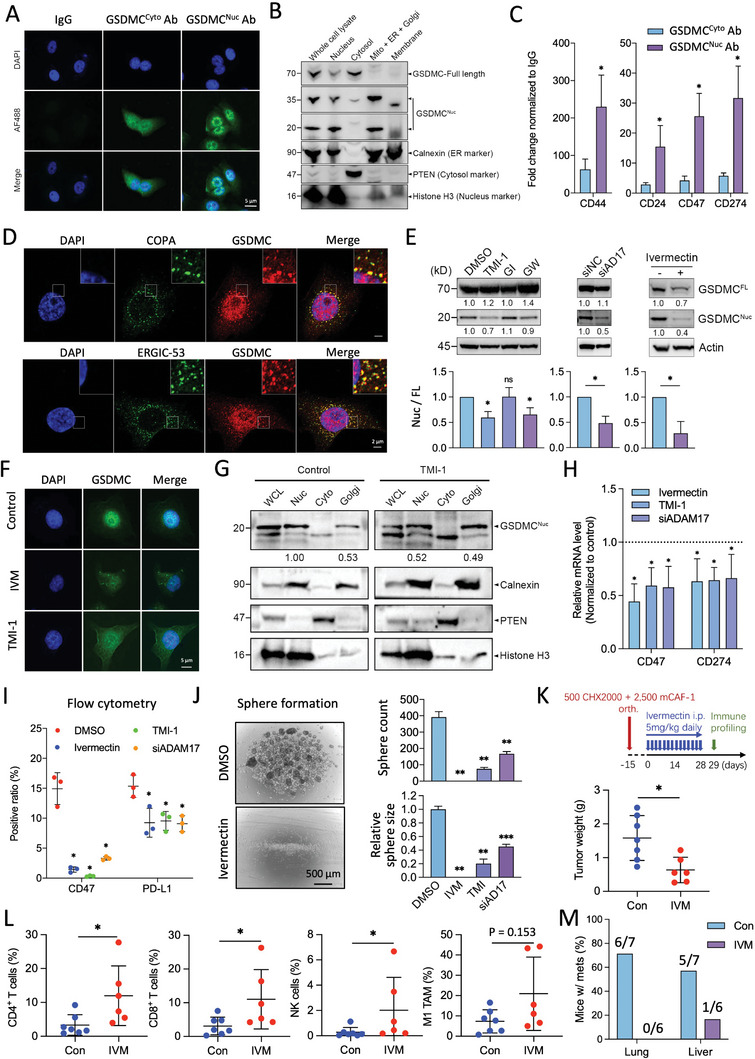
Nuclear GSDMC transcriptionally controls aggressiveness of PDAC. A) Immunofluorescence (green) for GSDMC using the cytoplasmic (GSDMC^Cyto^) and nuclear GSDMC (GSDMC^Nuc^) antibodies (ab) in SIC003 human PDAC cells. IgG is used as a negative control, while DAPI serves as a nuclear stain. B) Western blot analysis of cell fractionation showing the expression of full length (FL) and nuclear GSDMC in various cellular compartments of human SIC003 PDAC cultures. GSDMC‐FL and nuclear GSDMC are detected using the GSDMC^Nuc^ antibody. C) Fold change for ChIP‐qPCR of *CD24*, *CD44*, *CD47*, and *CD274* (PD‐L1) promotor DNA expression in PDAC cells following immunoprecipitation with the GSDMC^Cyto^ and GSDMC^Nuc^ antibodies (ab), or IgG as negative control (n = 3 independent samples). D) Immunofluorescence to co‐localize GSDMC (red) with COPA (green) or ERGIC‐53 (green). DAPI is utilized as a nuclear stain. E) Effects on GSDMC cleavage by inhibition of members of the ADAM family of proteases (ADAM17 inhibitor TMI‐1, ADAM10 inhibitor GI254023X [GI], ADAM10/17 inhibitor GW280264X [GW], si*ADAM17* [si*AD17*], and control siRNA [si*NC*]). Effect on GSDMC content by nuclear import inhibitor ivermectin (IVM; 5 µM). Representative Western blot (upper panel) and quantification (lower panel) (n = 3 independent samples). F) Immunofluorescence for GSDMC (green) in human SIC003 PDAC cultures in the presence of the ADAM17 inhibitor TMI‐1 and ivermectin. DAPI is used as a nuclear stain. G) Nuclear GSDMC protein levels in the whole cell (WCL), nucleus (Nuc), cytoplasm (Cyto), and Golgi, in the presence or absence of the ADAM17 inhibitor TMI‐1. A representative Western blot is displayed. H) qPCR fold change for *CD47* and *CD274* (PD‐L1) mRNA expression using the ADAM17 inhibitor TMI‐1, si*ADAM17*, and ivermectin. The dotted line indicates treatment with DMSO (control) and is set to 1.0 (n = 3 independent samples). I) Flow cytometry for CD47 and PD‐L1 surface expression in the presence or absence of the ADAM17 inhibitor TMI‐1, si*ADAM17*, or ivermectin. DMSO is used as a negative control (n = 3 independent samples). J) Sphere formation capacity of CHX2000 KPC cells expressed as number of formed spheres per 10,000 cells per 1 mL for treatment with ivermectin (IVM), the ADAM17 inhibitor TMI‐1, and si*ADAM17* compared to control (DMSO). Representative images (left panel), quantification of sphere count and size (right panel) (n = 3 independent samples; one‐way ANOVA analysis with Games‐Howell post‐hoc test). K) Schematic illustration of the treatment schedule for ivermectin in an orthotopic (orth.) PDAC model using a mixture of KPC‐derived CHX2000 cells and CAF‐1 cells (upper panel). The lower panel shows the tumor weight of the pancreatic tumors with and without ivermectin (IVM) treatment; n = 6‐7. L) Percentage of tumor‐infiltrating CD4^+^ T helper cells, CD8^+^ cytotoxic T cells, NK cells, and M1 tumor‐associated macrophages (TAM). M) Occurrence of lung and liver metastases, both with and without ivermectin (IVM) treatment, along with the ratio of mice showing metastases. N = 7 for Control group, N = 6 for IVM group. *p < 0.05, **p < 0.01, and ***p < 0.001; Mann–Whitney U test, two‐tailed, unless stated otherwise. Please also see Figure S5 (Supporting Information).

To dissect the downstream mechanism related to the release of nuclear GSDMC, which is detectable in PDAC cells in addition to cytoplasmic GSDMC (**Figure** **5**A), we investigated the subcellular distribution of GSDMC by immunofluorescence. Two types of antibodies were available for this study. The nuclear‐to‐cytoplasmic ratio of GSDMC expression was determined by scoring the staining intensity using the GSDMC^Cyto^ antibody compared to the GSDMC^Nuc^ antibody. Based on the analysis of 100 cells per group we concluded that the GSDMC^Nuc^ antibody identifies GSDMC in the nucleus (Figure S5A, Supporting Information).

To further corroborate these findings, the distribution of full‐length GSDMC and nuclear GSDMC across various cellular compartments was explored by Western blot analysis. The data show that besides being found in the nuclear fraction, nuclear GSDMC can also be detected both in the Mito + ER + Golgi fraction (Figure 5B). ChIP‐qPCR analysis demonstrated that predominantly nuclear GSDMC, rather than cytoplasmic GSDMC, binds to the promoter regions of various genes including those related to stemness (*CD44*) and immune checkpoints (*CD24, CD47*, and *CD274*) (Figure 5C). High‐resolution fluorescence microscopy revealed that GSDMC co‐localizes with the retrograde Golgi to ER marker COPA and the marker ERGIC‐53 that shuttles its cargo between ER and ERGIC^[^ ^18^ ^]^ (Figures 5D; Figure S5B, Supporting Information). This suggests a spatial association of GSDMC with key components of intracellular trafficking pathways. However, the specific role of GSDMC in retrograde protein trafficking between the Golgi and the ER, as mediated by COPA, and its potential involvement in anterograde transport mediated by ERGIC‐53, require further investigation. Notably, this co‐localization may have implications for the cellular functions of GSDMC, including the possibility of facilitating its translocation into the nucleus.

To shed further light on the proteolytic processing of GSDMC, we next tested the cleavage of GSDMC by various enzymes. We screened 120 common endopeptidases to identify enzymes capable of digesting GSDMC. Consulting the OncoDB data base, mRNA levels were increased for 32 out of these 120 enzymes in PDAC compared to normal tissues (data not shown). Kaplan‐Meier plot analysis determined that among these 32 genes, MME, CAPN1, CAPN2, CASP8, and ADAM17 were associated with poor relapse‐free survival (RFS), consistent with the poor RFS observed in GSDMC‐high patients (Figure 1J). Our ChIP‐seq data had revealed that GSDMC exhibited binding peaks on PREP, MBTPS1, LGMN, and CASP2. Finally, consultation of the existing literature suggested a potential role for CASP1, HTRA2, CTRC, and CTSH in cleaving GSDMC. This meticulous selection process provided us with a final list of 14 enzymes for further functional testing to determine their potential role in GSDMC digestion.

Intriguingly, upon inhibition of each of these 14 enzymes by siRNA, a significant reduction in nuclear GSDMC levels is only observed for si*ADAM17*, suggesting that ADAM17 is a key enzyme for cleaving GSDMC into its nuclear fragments (Figure S5C, Supporting Information). Next, we treated PDAC cells with various pharmacological inhibitors, i.e., the ADAM17 inhibitor TMI‐1, the ADAM10 inhibitor GI254023X (GI), and the ADAM10/17 inhibitor GW280264X (GW). As predicted by above siRNA experiments, specific inhibition of ADAM17 by TMI‐1, but not specific inhibition of ADAM10 by GI254023X, resulted in a significant decrease of nuclear GSDMC, which was comparable to the effect observed for genetic targeting of ADAM17 (Figure 5E). We confirmed that si*ADAM17* and TMI‐1 inhibit ADAM17 at the protein level and translated into reduced ADAM17 protease activity (Figure S5D, Supporting Information). Intriguingly, a non‐toxic dose (5 µM, Figure S5E, Supporting Information) of the antiparasitic drug ivermectin also acts as a potent inhibitor of GSDMC functions via inhibition of importin α/β, which plays an important role in the nuclear import of proteins such as nuclear GSDMC (Figure 5E). Upon treatment with the ADAM17 inhibitor TMI‐1 or ivermectin, nuclear GSDMC protein predominantly localized to the Golgi and showed reduced presence in the nucleus, as evidenced by Western blot and immunofluorescence (Figure 5F,G).

Functionally, inhibition of nuclear GSDMC by TMI‐1, si*ADAM17*, or ivermectin reduced mRNA levels for the downstream targets *CD47* and *CD274* (Figure 5H), and also reduced the cell surface expression of the encoded proteins CD47 and PD‐L1 as determined by flow cytometry (Figure 5I). Functionally, inhibition of ADAM17 by TMI‐1 or GW, or treatment with ivermectin resulted in a potent inhibition of 3D sphere formation (Figure 5J; Figure S5F, Supporting Information) and invasion (Figure S5G, Supporting Information) of PDAC cells. The particularly strong effects of GW might be attributed at least in part to the modulation of other pathways relevant for sphere formation in PDAC. After successfully demonstrating in vitro the efficacy of ivermectin as a pharmacological inhibitor for GSDMC (Figure 5E), we proceeded to systemic ivermectin treatment in vivo. An intraperitoneal dose of 5 mg kg^−1^ of ivermectin administered to our tumor‐bearing mice was well‐tolerated, which aligns well with the previously reported LD50 of 30 mg kg^−1^ for intraperitoneal ivermectin administration in healthy mice.^[^ ^19^ ^]^ Consistent with our data for *Gsdmc* silencing (Figure 4G,H), treatment of the orthotopic PDAC model with ivermectin (Figure 5K) also enhanced the influx of CD4^+^ and CD8^+^ T cells, and NK cells (Figure 5L), and subsequently inhibited tumor progression with virtual ablation of the metastases in liver and lung (Figure 5M).

ChIP‐seq analysis using the GSDMC^Nuc^ and GSDMC^Cyto^ antibodies demonstrated binding peaks for the GSDMC^Nuc^ antibody distributed throughout the entire genome, whereas the GSDMC^Cyto^ and IgG antibodies showed no relevant binding (Figure S5H–J, Supporting Information). The upstream region 2 kb before the transcription start site (TSS) showed the most enriched area of binding peaks (Figure S5I, Supporting Information). We then used MEME Suite to identify specific binding motifs and among others “TCTCGCGAGA” ranked highest, accounting for 722 GSDMC binding sites (Figure S5K, Supporting Information). Next, we performed GO and KEGG analyses to gain insight into the functions of the genes bound by GSDMC. The results indicated a wide range of biological processes, including vesicle transportation, mitochondrial function, and chromosome organization. KEGG analysis also highlighted the regulatory role of GSDMC in DNA repair and oncogenic pathways such as HIF‐1, TGFβ, and MAPK signaling pathways (Figure S5L,M, Supporting Information).

To further elucidate the molecular function of GSDMC for transcriptional regulation, we overexpressed various *GSDMC* constructs in PDAC cells (Figure S5N, Supporting Information): (1) *GSDMC* WT, (2) *GSDMC* mutant delta 310–331 (lacking the potential cleavage site for ADAM17), (3) *GSDMC* mutant delta 392–413 (lacking a potential DNA binding domain), and (4) *GSDMC* mutant delta 456–477 (lacking a different potential DNA binding domain). We employed qPCR analysis to assess whether the DNA‐binding ability of GSDMC had been compromised by mutating the zinc finger binding motifs and the potential cleavage site for ADAM17. While overexpression of *GSDMC* WT resulted in the anticipated upregulation of *CD24*, *CD44*, *CD47*, and *CD274* (PD‐L1), such upregulation was significantly attenuated for each of the three *GSDMC* mutants. These findings underscore the crucial role of GSDMC in regulating the expression of these genes and highlight its direct involvement in transcriptional regulation.

**Figure 6:**
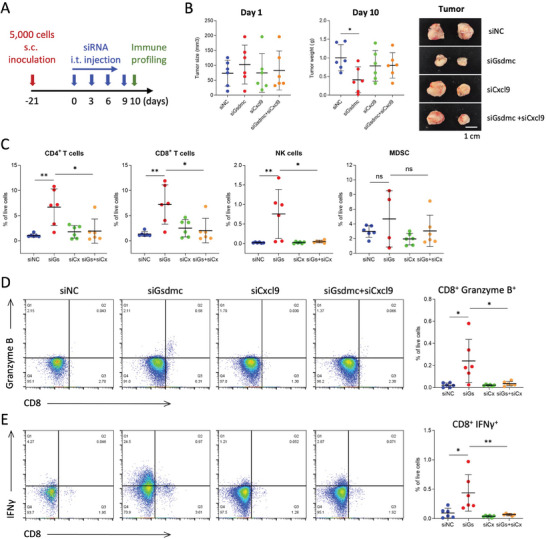
GSDMC inhibition unleashes CXCL9‐mediated influx of T cells. A) Schematic depicting administration of si*Gsdmc*(si*Gs*) and si*Cxcl9*(si*Cx*) as single treatments or in combination, compared with control treatment in a murine PDAC model. B) Tumor size (left), tumor weight (middle), and representative photographs of gross tumor morphology (right) in response to single or combinational treatment; n = 6. C) Tumor immunoprofiling by flow cytometry for CD4^+^ and CD8^+^ T cells, NK cells, and MDSCs following treatment with single or combinational siRNA. D) Infiltrating CD8^+^ cells were functionally characterized by the expression of granzyme B, and E) IFNγ, utilizing intracellular staining for flow cytometry analysis. *p < 0.05 and **p < 0.01; Mann–Whitney U test, two‐tailed. Please also see Figure S6 (Supporting Information).

In addition to transcriptionally controlling above immune checkpoint molecules, our RNA‐seq data also indicate that *Gsdmc* knockdown rescues the expression of multiple genes, including the chemokine Cxcl9 (Figure S2L, Supporting Information), which might contribute to the recruitment of immune cells, particularly cytotoxic T cells.^[^ ^20^ ^]^ We validated the upregulation of *Cxcl9* following *Gsdmc* knockdown in murine PDAC cells by qPCR (Figure S6A, Supporting Information). To formally test our hypothesis that *Cxcl9* upregulation plays an important role in the enhanced infiltration of immune cells, such as CD8 T cells, following *Gsdmc* knockdown, we treated PDAC‐bearing mice with si*Gsdmc*, si*Cxcl9*, or the combination of both (**Figure** **6**A; Figure S6B, Supporting Information). Intriguingly, the si*Gsdmc*‐induced reduction in tumor size was virtually abrogated by co‐treatment with si*Cxcl9* (Figure 6B). Consistently, the si*Gsdmc*‐mediated infiltration of CD4^+^ and CD8^+^ T cells, and NK cells into the tumor was reversed by si*Cxcl9*, while no effect was seen on myeloid cells such as MDSCs (Figure 6C). Importantly, the influx of the functionally most relevant subsets of CD8^+^ cells co‐expressing IFNγ and granzyme B, respectively, was equally suppressed by the combined treatment (Figure 6D,E). In summary, these findings demonstrate that in preclinical PDAC models silencing of Gsdmc unleashes CXCL9 as a key downstream mechanism for the TME‐modulating effects of GSDMC, resulting in an uprise of tumor‐infiltrating immune cells and a subsequent reversal of tumor progression.

### 2.6 Identification of Upstream Regulators of GSDMC Expression

**Figure 7:**
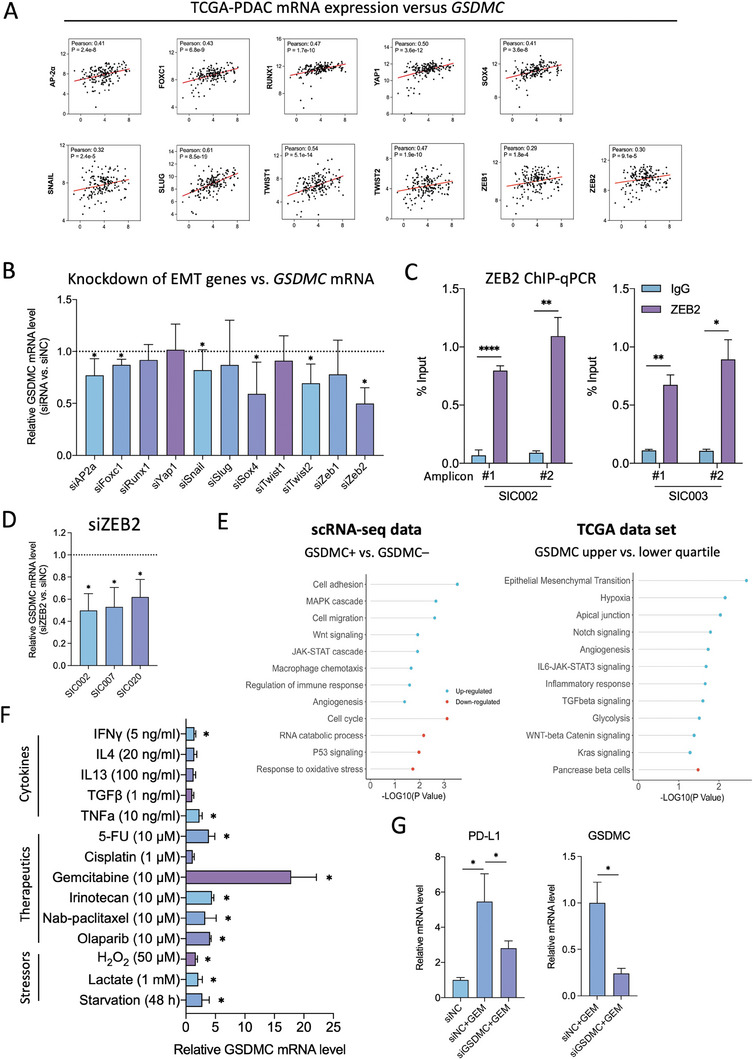
Identification of upstream modulators of GSDMC expression. A) Correlation analysis between mRNA expression for *GSDMC* versus several EMT‐related genes using TCGA datasets. B) qPCR fold change showing *GSDMC* mRNA expression following knockdown of EMT‐related genes using siRNA in SIC002 PDAC cells. The expression level observed with control siRNA (si*NC*) is represented by the dotted line (n = 3 independent samples). C) *ZEB2* ChIP‐qPCR analysis following pull‐down with antibodies for ZEB2 or IgG as a control for two different PDAC cultures. Results are shown as input DNA in % for amplicon #1 and #2 within the *GSDMC* promoter (n = 3 independent samples; two‐tailed t‐test). D) qPCR fold change of *GSDMC* in three different human primary PDAC cell cultures following pre‐treatment with si*ZEB2*. The dotted line represents control treatment with si*NC* (n = 3 independent samples). E) Pathway enrichment analysis for GSDMC^+^ versus GSDMC^–^ PDAC cells using our single‐cell RNA sequencing (scRNA‐seq) data (left panel). Pathway enrichment analysis for patients in the TCGA dataset stratified for tumor *GSDMC* expression in the upper versus lower quartile (right panel). Up‐regulated pathways are denoted in blue, while down‐regulated pathways are depicted in red. F) Relative mRNA expression levels for *GSDMC* in response to a diverse range of upstream activators for 48 hours (n = 3 independent samples). G) qPCR fold change of mRNA levels for *CD274* (left) and *GSDMC* (right) in PDAC cells treated with gemcitabine (GEM: 10  µM) in the presence of si*NC* or si*GSDMC* (n = 3 independent samples). *p < 0.05, **p < 0.01, and ****p < 0.0001; Mann–Whitney U test, two‐tailed, unless stated otherwise. Please also see Supplementary Figures S7.

GSDMC expression is induced by MCM and specifically by the TAM‐derived cytokine OSM (Figure 1A,E; Figure S1C, Supporting Information). Analysis of the TCGA data revealed a significant correlation of *GSDMC* with various EMT‐related genes (**Figure** **7**A; Figure S7A, Supporting Information), which encouraged us to further explore the role of EMT in regulating *GSDMC* transcription. Upon systematic knockdown of various EMT‐related genes, inhibition of *ZEB2* resulted in the most pronounced downregulation of *GSDMC*, whereas the effects for *TWIST2* and *SOX4* were slightly less prominent (Figure 7B). ChIP‐qPCR analysis for *ZEB2* validated its binding to the *GSDMC* promoter region (Figure 7C; Figure S7B, Supporting Information), suggesting that *ZEB2* could be an upstream regulator of *GSDMC*. Indeed, RNA interference for *ZEB2* (si*ZEB2*) resulted in a significant decrease in the expression of *GSDMC* compared to control treatment with si*NC* (Figure 7D), indicating that *GSDMC* is modulated by this important EMT regulator in PDAC. Consistent data were also obtained for SOX4 (Figure S7C, Supporting Information).

Moreover, the 10x Genomics single‐cell RNA‐Seq analysis, SMART single‐cell RNA‐Seq analysis and TCGA transcriptomic datasets suggested upregulation of various other pathways in *GSDMC* ^*+*^ PDAC cells (Figure 7E; Figure S7A,D,E, Supporting Information), suggesting that *GSDMC* expression might also be modulated by various other upstream activators. Therefore, we explored the potential of several cytokines (IFNγ, TNFα, IL‐4, IL‐13, and TGFβ), chemotherapeutic drugs (5‐FU, gemcitabine, irinotecan, nab‐paclitaxel, cisplatin), targeted therapies (e.g., the PARP inhibitor olaparib), oxidative stress inducers (e.g., H_2_O_2_), and metabolic modulators (lactate acidification, 48‐hour serum‐free starvation) for inducing *GSDMC* expression. Surprisingly, most upstream activators caused only a moderate induction of *GSDMC* expression, while the standard chemotherapeutic agent gemcitabine induced a very prominent 15‐20‐fold upregulation of *GSDMC* at 10 µM (Figure 7F), a trend that was still evident for low‐dose gemcitabine treatment at 1 µM (Figure S7G, Supporting Information). This was accompanied by a 5‐6‐fold upregulation of the immune checkpoint *CD274* (PD‐L1), which could be rescued by simultaneous knockdown of *GSDMC* (Figure 7G). Notably, gemcitabine simultaneously induced EMT‐related genes, including vimentin, N‐cadherin, and, most prominently, *ZEB2* mRNA (Figure S7F, Supporting Information). These data suggest that gemcitabine most likely induces *GSDMC* via upregulation of ZEB2.

Together these data demonstrate for PDAC highly cell‐specific effects of GSDMC in the context of EMT induction and chemotherapy. Activation of *GSDMC* consistently results in enhanced stemness and immune evasion. Based on these findings, we propose a mechanism by which TAM‐derived cytokines, including OSM, induce EMT genes, such as *ZEB2*, which then results in the upregulation of *GSDMC*. The subsequent cleavage of GSDMC by ADAM17 leads to the release of fragments, which translocate into the nucleus and bind to the promoter regions of genes critically determining PDAC aggressiveness and immune evasion, i.e., *CD24, CD44, CD47*, and *CD274* (PD‐L1). At the same time, GSDMC suppresses the chemokine CXCL9, which acts as a chemoattractant for T cells, NK cells, and other immune cells (Figure S7H, Supporting Information).

### 2.7 Therapeutic Targeting of Gsdmc in Preclinical PDAC Models

To demonstrate the potential translational value of *Gsdmc* targeting in PDAC, we treated established murine PDAC models by injecting siRNA against *Gsdmc*. First, we used intratumoral injections of si*Gsdmc* versus si*NC* every three days as proof‐of‐concept (Figure S8A, Supporting Information). This approach resulted in rapid and strong inhibition of tumor growth on day 21 after initiation of treatment. To ensure the effectiveness of the si*Gsdmc* treatment, we analyzed the siRNA‐treated tumors using Western blot analysis, which confirmed the downregulation of Gsdmc to below 50% (Figure S8B, Supporting Information). Histological analysis of the tumor tissue revealed that si*Gsdmc*‐treated tumors exhibited more cleaved caspase 3 signals as compared with si*NC*‐treated tumors. In contrast, during Ki‐67 staining to assess proliferation, we did not observe any differences. These data suggest that si*Gsdmc*‐mediated knockdown in syngeneic tumors primarily results in increased apoptosis, rather than reduced proliferation (Figure S8C, Supporting Information). Moreover, we found a more pronounced influx of CD3^+^, CD8^+^, and CD4^+^ immune cells (Figure S8D, Supporting Information).

**Figure 8:**
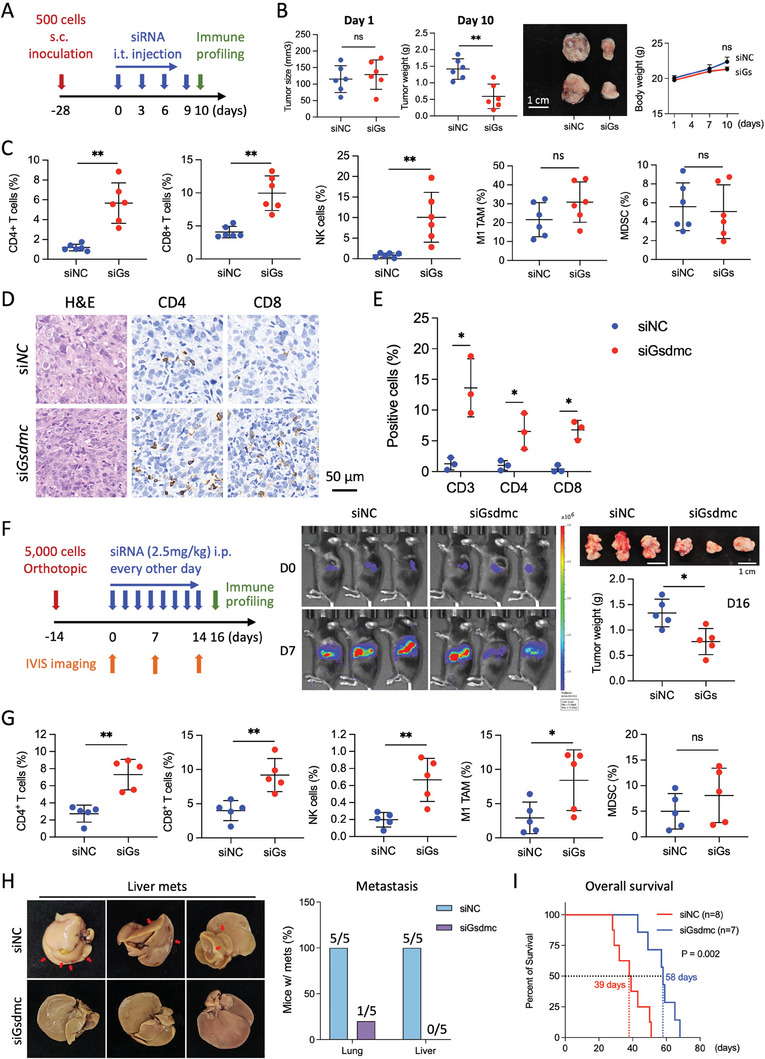
Therapeutic targeting of *Gsdmc* in preclinical PDAC models. A) Schematic illustrating the experimental design for the subcutaneous murine PDAC model treated with intratumoral si*Gsdmc* (si*Gs*) or si*NC* injections. B) Tumor size of si*Gsdmc* or si*NC*‐treated tumors on day 1 (left), tumor weight on day 10 with corresponding photographs depicting the macroscopic morphology of the explanted tumors (middle), and body weight over the course of the experiment (right). N = 6 for both groups. C) Immune profiling of tumor‐infiltrating cells by flow cytometry, depicting the percentages of CD4^+^ cells, CD8^+^ cells, NK cells, M1 TAMs, and MDSCs in si*Gsdmc*‐treated versus si*NC*‐treated tumors. N = 6 for both groups. D) Representative images for H&E staining and immunohistochemistry for CD4 and CD8 in tumor sections derived from si*Gsdmc*‐treated versus si*NC*‐treated mice. E) Quantification of cells that stained positive for the listed T cell markers. N = 3 for both groups. F) Schematic illustrating the experimental design for orthotopic murine PDAC models treated with systemic si*Gsdmc* or si*NC* injections (left panel). Representative IVIS images recorded at baseline and on day 7 (middle panel). Representative photos and weight of tumors harvested on day 16 (right panel). N = 5 for both groups. G) Immune profiling of tumor‐infiltrating cells by flow cytometry, depicting the percentages of CD4^+^ cells, CD8^+^ cells, NK cells, M1 TAMs, and MDSCs in si*Gsdmc*‐treated versus si*NC*‐treated tumors. The indicated percentage represents the proportion of live cells. N = 5 for both groups. H) Representative images of liver metastases and enumeration of lung and liver metastases. I) Kaplan Meier analysis for overall survival of si*Gsdmc*‐treated (n = 7) versus si*NC*‐treated (n = 8) mice. Statistical analysis was performed using the log‐rank test. *p < 0.05 and **p < 0.01; Mann–Whitney U test, two‐tailed. Please also see Figure S8 (Supporting Information).

Next, we treated larger PDAC tumors with the same treatment regimen, but harvested the tissue one day after the last siRNA injection to allow for more extensive immune profiling by flow cytometry (**Figure** **8**A,B). Even after this short period of treatment, tumor growth was already significantly reduced (Figure 8B). Consistent with previous results, we observed a significant increase in CD4^+^ and CD8^+^ T cells, as well as NK cells, as determined by flow cytometry (Figure 8C). We validated these flow cytometry results by immunohistochemical analysis, which demonstrated a consistently augmented infiltration of CD3^+^, CD4^+^, and CD8^+^ T cells within the tumor after si*Gsdmc* treatment (Figure 8D,E). We then incorporated additional functional markers for a more comprehensive characterization of the immune properties and their spatial context. First, we conducted double staining for the pan T cell marker CD3 and the pore‐forming cytotoxic molecule perforin in si*Gsdmc*‐treated murine tumor sections (Figure S8E, Supporting Information).

Next, we used CODEX multiplex imaging to provide a comprehensive understanding of the spatial distribution of the different cell types and their tissue composition. The antibody panel included the T cell markers CD4 and CD8, the NK cell marker NKp46, the M2‐TAM marker arginase 1 (Arg1), the epithelial marker panCK, and the proliferation marker Ki‐67. The results conclusively demonstrate a substantial influx of lymphoid anti‐tumor immune cells, such as CD4, CD8 T cells, and NK cells upon *Gsdmc* inhibition, accompanied by a reduction in M2‐type TAMs (Figure S8F,G, Supporting Information). Due to limitations in available anti‐mouse antibodies suitable for CODEX, we could not stain for M1 TAM. Staining for panCK to identify cancer cells was notably diminished, consistent with the decrease in cancer cell content. Co‐localization of the proliferation marker Ki67 revealed a strong proliferative activity for invading anti‐tumor CD4 T cells, CD8 T cells, and NK cells. Taken together, CODEX imaging indicates that most cancer cells have been replaced by proliferating anti‐tumor immune cells.

Finally, we performed survival experiments and treated PDAC‐bearing mice with systemic injections of siRNA (2.5 mg kg^−1^) using the Entranster in vivo transfection reagent. siRNA typically has a limited lifespan within cells, not exceeding three to five days. Therefore, we decided to inject siRNA every other day. Very effective knockdown of GSDMC in murine orthotopic tumors following four consecutive siRNA intraperitoneal injections could be validated by Western blot (Figure S8B, Supporting Information). A first set of mice was harvested on day 16 and showed the expected reduced tumor growth (Figure 8F). Flow cytometry revealed a consistent remodeling of the tumor microenvironment (Figure 8G) and virtually erased the metastatic activity of the PDAC cells (Figure 8H). A second set of mice was continuously treated until humane endpoints were reached to assess the overall survival. Median survival for this very aggressive murine PDAC model increased from 39 days for si*NC* to 58 days for si*Gsdmc* (Figure 8I; Figure S8H, Supporting Information). Together our data represent compelling evidence that systemic targeting of *Gsdmc* by siRNA is feasible, well‐tolerated, and drastically reduces key components for the aggressiveness of PDAC, namely stemness and immune evasion.

As tumor size was already altered very early after initiation of treatment, we investigated whether changes in the immune profile might be confounded by the differences in tumor sizes. First, when comparing tumors of equal size for the two groups shown in Figure 8A, we found consistent differences in the composition of the immune cells for si*Gsdmc* versus si*NC* (Figure S8I, Supporting Information), as noted for the comparison of the entire groups (Figure 8C). Second, inhibition of *Gsdmc* did not alter the in vivo proliferative capacity of PDAC cells (Figure S4E,S8C, Supporting Information). Together these data strongly suggest that the observed changes in the composition of the TME of si*Gsdmc*‐treated versus si*NC*‐treated mice are at least in part due to reprogramming of the TME following downregulation of *Gsdmc*.

**Figure 9:**
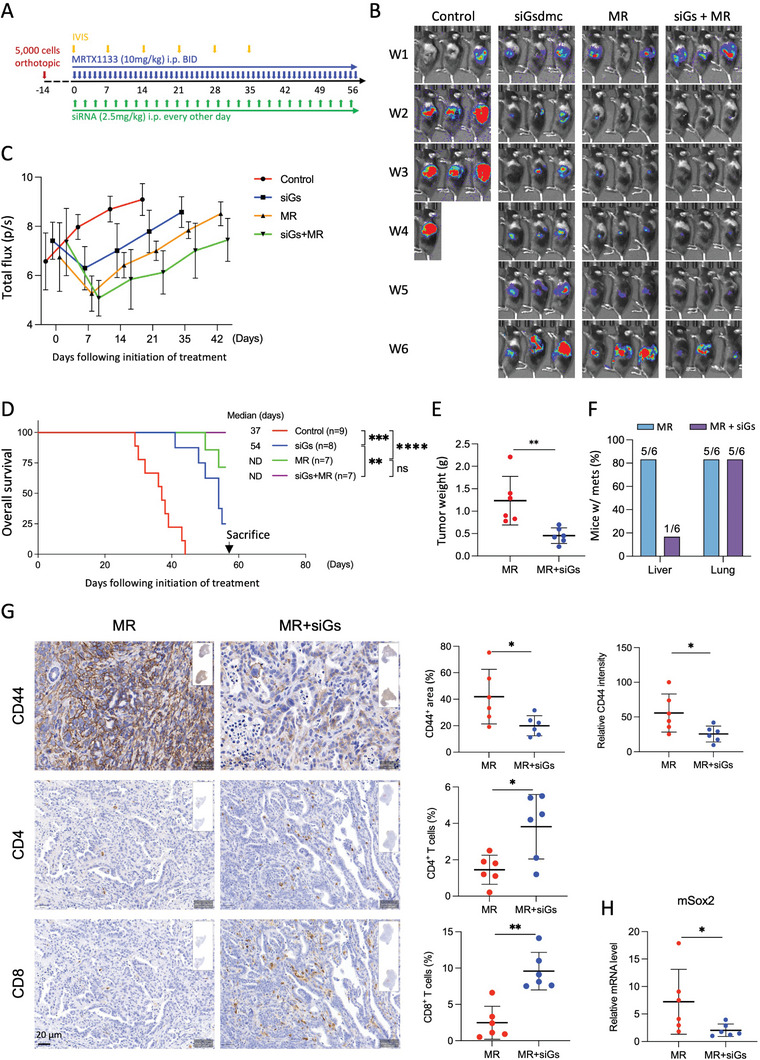
Combined inhibition of *Gsdmc* and KRAS delays PDAC progression. A) Schematic illustrating the treatment strategy for si*Gsdmc* and KRAS^G12D^ protein inhibition using MRTX1133 in an orthotopic murine PDAC model, either as single treatment or in combination. MRTX1133 was administered twice daily, while si*Gsdmc* was injected every other day. B) Representative bioluminescence analysis comparing mice treated with control, si*Gsdmc* (siGs) alone, MRTX1133 (MR) alone, or their combination over 6 weeks (W1 to W6). C) Follow‐up of tumor progression by IVIS as an indicator of tumor load. D) Survival analysis, E) tumor weight, and F) metastatic burden according to allocated treatment regimens. G) Top panel shows representative immunohistochemistry for the expression of the cancer stem cell marker CD44 (upper left), along with quantification of CD44^+^ area and intensity (right) in tumor tissue of mice treated with the combination of si*Gsdmc* and MRTX1133 versus MRTX1133 alone. Middle and lower panels show representative immunohistochemistry for tumor‐infiltrating CD4^+^ and CD8^+^ T cells (left), with corresponding quantification (right) by the combination treatment versus MRTX1133 alone; n = 6. H) Expression levels of the stemness gene *Sox2* according to allocated treatment. *p < 0.05, **p < 0.01, and ***p < 0.001; Mann–Whitney U test, two‐tailed, unless stated otherwise.

Although Gsdmc targeting significantly extended the median survival of murine PDAC models, eventually all mice succumbed from disease progression. Therefore, we next investigated the potential treatment benefits of combining *Gsdmc* targeting with KRAS^G12D^ inhibition using MRTX1133, a new small molecule inhibitor that is currently under clinical investigation (NCT05737706) (**Figure** **9**A–C). Notably, previous preclinical studies showed that KRAS^G12D^ inhibition alone, while effective initially, eventually also resulted in tumor relapse of highly aggressive murine PDAC models.^[^ ^21^ ^]^ Our studies now show a significant improvement in overall survival for the addition of MRTX1133 compared to si*Gsdmc* alone (Figure 9D). Unfortunately, the difference in overall survival for the combination therapy compared to MRTX1133 alone did not reach statistical significance at the prespecified time of conclusion of the experiment (day 56). The duration of the experiment was restricted due to limited availability of MRTX1133 and prioritization on molecular endpoints such as flow cytometry, histology, and RNA analyses of the tumor tissue.

Indeed, several significant improvements were noted for the combination treatment compared to MRTX1133 alone. First, we observed significantly lower tumor weights for the combination treatment group compared to MRTX1133 alone (Figure 9E). Second, the combinational treatment led to virtual ablation of macroscopic liver metastases, although interestingly lung metastases remained unaffected (Figure 9F). Immunostaining for the stemness marker CD44 revealed a reduction in CD44^+^ area and signal intensity for tumors treated with the combination therapy compared to single MRTX1133 treatment (Figure 9G). Consistently, decreased mRNA levels of the stemness gene *Sox2* were recorded for the combined treatment (Figure 9H), suggesting an attenuation of stemness features for these PDAC tumors. Finally, the combination therapy further and markedly increased tumor‐infiltrating CD8 and CD4 cells (Figure 9G), indicating a strong translational potential for this new treatment strategy in PDAC.

## 3 Discussion

We have made a significant discovery about GSDMC's role in PDAC aggressiveness and immune evasion, independent of its known pyroptosis effects. We have linked EMT‐related factors to GSDMC's regulation and explored its impact on stemness and immune evasion. Our study provides compelling evidence for GSDMC's control over these crucial features in PDAC, shedding new light on its role in disease progression. Unlike prior bioinformatics‐focused work,^[^ ^22^, ^23^ ^]^ we integrated genetic and pharmacological interventions, identifying ADAM17 as a key GSDMC regulator, offering crucial mechanistic insights.

GSDMC belongs to the gasdermin family, comprising six related proteins sharing about 45% sequence homology. These proteins feature a cytotoxic N‐terminal domain and a C‐terminal repressor domain. The N‐terminal domain forms pores in the cell membrane, potentially inducing pyroptosis, an inflammation‐induced cell death form. Prior research has mainly focused on GSDMC's role in pyroptosis, triggered by the metabolite α‐ketoglutarate (α‐KG), leading to caspase‐8‐mediated cleavage of GSDMC.^[^ ^24^ ^]^

However, our understanding of the involvement of GSDMC in cancer, particularly pancreatic cancer, remained limited. Miguchi et al. reported that the depletion of Apc and Tgfbr2 led to adenocarcinoma formation in the colon, accompanied by a significant increase in *Gsdmc* expression.^[^ ^25^ ^]^ Silencing *Gsdmc* led to decreased proliferation and tumorigenesis of colorectal cancer cell lines, while its overexpression enhanced their proliferation. Interestingly, our data reveal a positive association between Tgfb2 and Gsdmc in pancreatic cancer, contrasting with the inverse relationship in colorectal cancer. Studies by Cui et al. and Sun et al. linked high *GSDMC* expression to poor survival in kidney renal clear cell carcinoma and breast invasive carcinoma, respectively.^[^ ^26^, ^27^ ^]^ In lung adenocarcinoma, elevated GSDMC expression was also associated with a negative outcome.^[^ ^28^ ^]^ A recent systems analysis for pancreatic cancer suggested GSDMC's potential role in regulating PDAC cell proliferation.^[^ ^29^ ^]^ These findings consistently indicate that *GSDMC* overexpression is linked to poor outcomes in various cancer types, but the functional implications and underlying mechanisms remained elusive.

Our studies, using human and mouse models of pancreatic cancer, challenged the bioinformatic prediction of GSDMC's role in PDAC cell proliferation. Instead, we revealed its pivotal role in driving PDAC progression, with significant upregulation in PDAC tumors compared to normal tissue. Low *GSDMC* expression correlated with improved survival, particularly in CD8^+^ T cell‐enriched tumors of PDAC patients. Under EMT‐inducing conditions, *GSDMC* expression was consistently upregulated in primary PDAC cells, associated with EMT‐related pathways in GSDMC^+^ PDAC datasets. Silencing *GSDMC* altered cell morphology, reduced invasion, and decreased cancer stem cell numbers. In vivo, *GSDMC* knockdown in PDAC reduces tumorigenicity, and liver and lung metastases.

Prompted by reported correlations between GSDMC expression and immune parameters in breast cancer,^[^ ^27^ ^]^ we focused our investigation on the role of GSDMC in fully immunocompetent PDAC models. *Gsdmc* knockdown resulted in a remarkable reduction in tumor growth, liver, and lung metastases, with increased survival rates in sh*Gsdmc* mice. This contrasted with unaffected in vitro and in vivo proliferative capacity in sh*Gsdmc* PDAC cells. Immune profiling of sh*Gsdmc* tumors revealed comprehensive remodeling of the tumor microenvironment, characterized by decreased immunosuppressive cells like MDSCs and M2 TAMs, alongside increased anti‐tumor immune cells, such as M1 TAMs, cytotoxic CD8^+^ cells, and T‐helper CD4^+^ cells.

Our subsequent mechanistic investigations reveal the functional significance of GSDMC in immune evasion in PDAC. We identified critical immune checkpoints regulated by GSDMC on PDAC cells, including CD47, CD24, CD44, and PD‐L1. Specifically, CD47 interacts with SIRPα on tumor‐associated macrophages, inhibiting macrophage‐mediated phagocytosis and promoting immune evasion. CD24 serves as another “don't eat me” signal,^[^ ^30^ ^]^ interacting with the inhibitory receptor Siglec‐10 on tumor‐associated macrophages. Additionally, CD24 is implicated in cancer stem cell maintenance and tumor progression.^[^ ^9^ ^]^ CD44, a cell surface glycoprotein, plays roles in cell adhesion, migration, and cancer stemness.^[^ ^9^ ^]^ PD‐L1, expressed on the surface of cancer cells, inhibits T cell activation via interaction with PD‐1, facilitating immune evasion.^[^ ^31^ ^]^

Moreover, in our investigation into Gsdmc downregulation, we discovered a notable increase in *Cxcl9* expression, crucial for recruiting anti‐tumor immune cells like cytotoxic T cells and NK cells via the CXCL9/CXCR3 axis. Inhibiting Cxcl9 suppressed the influx of functionally relevant subsets of CD8^+^ cells co‐expressing IFNγ and granzyme B, highlighting CXCL9's role in mediating the effects of Gsdmc silencing in PDAC models.^[^ ^20^ ^]^ Our findings are consistent with studies linking CXCL9 to anti‐PD(L)−1‐mediated CD8 T cell‐dependent immunity and improved outcomes in advanced PDAC patients undergoing chemotherapy.^[^ ^32^, ^33^ ^]^ Overall, our study sheds light on the intricate interplay between GSDMC, CXCL9 expression, and immune cell infiltration in PDAC, highlighting Gsdmc's central role in disease progression.

Our study investigated the subcellular localization and proteolytic processing of GSDMC. Gasdermins undergo cleavage by various proteases, including caspases (caspase‐1, caspase‐4/5/11, and caspase‐8),^[^ ^34^ ^]^ granzyme B, and neutrophil elastase.^[^ ^35^ ^]^ In PDAC, we found ADAM17 to play a central role in GSDMC cleavage, consistent with its association with PDAC progression.^[^ ^36^ ^]^ Pharmacological inhibition of ADAM17^[^ ^37^ ^]^ reduced GSDMC cleavage, leading to diminished nuclear accumulation of its fragments and the elimination of stemness‐associated phenotypes in PDAC cells. ChIP qPCR confirmed the binding of nuclear GSDMC to downstream target promoters (CD24, CD44, CD47, and CD274), supported by overexpression of a mutated GSDMC lacking the ADAM17 binding motif, which reversed target upregulation.

ER‐Golgi transport and nuclear import are closely linked processes. Newly synthesized proteins undergo post‐translational modifications in the ER before being transported to the Golgi for further processing. Some proteins, like transcription factors, are destined for the nucleus and are recognized by nuclear import receptors, such as importin α/β. Ivermectin inhibits importin α/β nuclear import specifically.^[^ ^38^ ^]^ Discovered in the 1970s, ivermectin is a potent antiparasitic drug derived from soil bacteria, recognized for its effectiveness against river blindness and lymphatic filariasis.^[^ ^39^ ^]^ More recently, Hou et al. found that ivermectin inhibits hypoxia‐induced nuclear translocation of PD‐L1 and GSDMC in breast cancer cells, blocking non‐canonical pyroptosis.^[^ ^40^ ^]^ Intriguingly, our study showed that ivermectin suppresses nuclear accumulation of GSDMC in PDAC, reducing sphere formation and invasiveness in vitro, akin to ADAM17 inhibition. In vivo, ivermectin treatment increased CD8^+^ T cell infiltration, reduced metastasis, and hindered tumor progression in PDAC.

Finally, we identified potential upstream regulators that govern the expression of GSDMC in PDAC. We discovered ZEB2 as a strong driver of GSDMC expression in PDAC. ZEB2, a transcription factor associated with EMT and invasiveness,^[^ ^41^ ^]^ directly binds to the promoter region of GSDMC. While other factors modestly upregulated GSDMC, gemcitabine notably increased its expression by 15‐20‐fold and induced PD‐L1 expression, partially rescued by GSDMC knockdown. These findings are in line with previous reports indicating that non‐toxic doses of gemcitabine can induce an EMT‐like phenotype^[^ ^1^, ^2^ ^]^ and subsequently result in the upregulation of various genes related to PDAC aggressiveness, e.g., the stemness markers CD44 and CD133. Further research is needed to explore the effects of GSDMC inhibition on modulating these undesired responses to gemcitabine.

Importantly, as our previous studies have shown, cancer stem cells are essential for tumor initiation and metastasis, but once solid tumors have established, cancer stem cells appear to become mostly dispensable for primary tumor growth that is driven by the expansion of their highly proliferative progenies.^[^ ^8^, ^10^, ^42^ ^]^ Therefore, GSDMC inhibition alone primarily prevents tumor initiation and metastasis, when immune surveillance is also particularly relevant, leading to increased median survival. However, tumor growth eventually progresses despite GSDMC inhibition, as differentiated PDAC cells continue to proliferate and immune surveillance wanes. Hence, the integration of GSDMC inhibition to enhance immune response, as shown in our study, with PD‐1/PD‐L1 inhibitors, or in conjunction with an anti‐proliferative treatment strategy like targeted therapy with KRAS^G12D^ inhibition, presents a promising new therapeutic paradigm. Expanding our exploration of *Gsdmc* silencing effects, we evaluated whether combining si*Gsdmc* with the novel KRAS^G12D^ inhibitor MRTX1133 may improve outcomes.^[^ ^21^, ^43^, ^44^ ^]^ In our study, combining siGsdmc with MRTX1133 improved overall survival compared to siGsdmc alone. Moreover, we observed notable reductions in tumor weight and the incidence of macroscopic liver metastases with the combinational treatment. Additionally, the combination therapy substantially downregulated the expression of the stemness gene *Sox2* and the cancer stem cell marker CD44, contrasting with the effects of single MRTX1133 treatment. Remarkably, the combination therapy significantly increased the infiltration of tumor‐infiltrating CD8 and CD4 cells, indicative of an enhanced immune response against the tumor. Further research is ongoing to refine this therapeutic strategy.

In summary, we show that GSDMC is consistently overexpressed in invasive PDAC cells, promoting disease progression by enhancing stemness, invasiveness, and immune evasion through upregulating immune checkpoint proteins. Targeting GSDMC genetically or pharmacologically in murine models reduces tumor growth and metastasis, partly by reprogramming the immunosuppressive tumor microenvironment. These findings align with previous reports describing pyroptosis‐independent effects of other gasdermin family members.^[^ ^15^ ^]^ Mechanistically, ADAM17 cleaves GSDMC, releasing its C‐terminal fragment, which then translocates into the nucleus to regulate genes associated with stemness and immune evasion. Thus, GSDMC emerges as a key regulator of PDAC aggressiveness, suggesting its potential as a therapeutic target to enhance susceptibility to immunotherapies, warranting further investigation.

## 4 Experimental Section

### Culture of Primary Human PDAC Cells

Primary tumors (SIC020, 021, and 032) and CTCs (SIC001, 002, 003, 005, and 007) from PDAC patient samples were collected following patients’ informed consent and propagated at Shanghai Jiaotong University School of Medicine (reference 2013‐0905‐70).^[^ ^45^ ^]^ Briefly, CTCs were isolated by VAR2‐coated magnetic beads (Dynabeads, ThermoFisher, 11533D) followed by organoid‐like cell culture. Cells were cultured in RPMI media supplemented with 10% FBS (#10270106, Gibco) and penicillin/streptomycin (#15140122, Invitrogen). The inhibitors GW280264X (#AOB3632, Aobious, 10 µM), GI254023X (#S8660, Selleck Chemicals, 20 µM), TMI‐1 (#HY101448, MedChem, 10 µM), ivermectin (#A2813, ApexBio, 5 µM) were dissolved in DMSO and applied to cell cultures at the indicated final concentrations.

### Proliferation

Cells were seeded into 96‐well plates (#353072, Corning) and monitored using the Incucyte Live‐Cell Analysis System (Sartoris) for one week.

### Sphere Formation Assay

Primary cell suspensions were cultured for seven days in an Ultra‐Low Attachment plate (#3471, Corning) at a density of 10,000 cells mL^−1^ in DMEM‐F12 supplemented with 1X B‐27, 20 ng mL^−1^ bFGF, penicillin/streptomycin, and amphotericin B (#15290026, Thermo).

### Spheroid Invasion Assay

Appropriate numbers of spheres (diameter >40 µm) formed by primary PDAC cells and murine KPC cells were suspended in 300 µL RPMI (+20% FBS). The cell suspension was then mixed with 200 µL Type I collagen (#354236, Corning), resulting in a final concentration of 1.5 mg mL^−1^. The mixture was transferred into an Ultra‐Low Attachment plate (#3473, Corning). Prior to this step, the collagen was neutralized with 3 uL of 1 M NaOH. The mixture was allowed to solidify for 30 minutes at RT. Then, 500 µL RPMI (+10% FBS) was added on top of the sphere‐collagen gel, the plate was incubated for 24 hours and the percentage of spheres that have invaded the surrounding matrix was determined.

### Transwell Invasion Assay

Transwell inserts (#3422, Corning) were coated with 100 µL Growth Factor Reduced Matrigel (#354230, Corning) for 2 hours at 37 °C. The supernatant was then aspirated before cells were seeded into insert, then the entire chamber was incubated at 37 °C for the indicated period. Different primary human PDAC cells and murine KPC cells were subjected to diverse initial seeding densities and incubation times: SIC002 and SIC005 (2.5 × 10^4^ for 48 hours), SIC003 and CHX2000 (2.5 × 10^4^ for 24 hours). After incubation, the remaining cells in the insert were removed using a cotton swab, and the insert was fixed with 4% paraformaldehyde for 10 minutes at RT. Subsequently, the insert membrane was stained with 0.1% crystal violet (#C6158, Sigma). Images of the insert membrane were captured using the EVOS Imaging System (Thermo Fisher).

### PBMC Isolation and MCM Preparation

Human peripheral blood‐derived mononuclear cells were obtained from healthy donors with informed consent. Mononuclear cells were isolated by density gradient centrifugation using Ficoll‐Paque Plus media (#17144003, Cytiva) and cultured in IMDM (#12440061, Invitrogen) supplemented with 10% human AB serum (#6914, Sigma) in Nunc EasYFlask (#156499, Thermo Fisher). Monocytes were obtained as adherent cells after 24 hours, following washes with PBS to remove non‐adherent cells. M2 monocyte‐derived macrophages were generated by treating cells with 0.5 ng mL^−1^ M‐CSF (#30025100, PeproTech) for three days, with the culture medium changed daily. From day 4 onwards, when monocytes had differentiated into macrophages, the media were replaced with conditioned media (DMEM/F12 plus 20 ng mL^−1^ bFGF [#10018C, PeproTech] plus 0.1X B‐27 [#17504044, Thermo]), and cultured for additional two days. The conditioned media were collected, centrifuged, and the supernatant was used as MCM.

### In Vitro Macrophage Phagocytosis Assay

PBMC, immortalized Bone Marrow‐Derived Macrophage (iBMDM) cells, or THP‐1 cells activated with PMA (#S1819, Beyotime, 100 ng mL^−1^ for three days) were labeled with PKH26 (#MX4021, MaokangBio), while cancer cells were labeled with PKH67 (#MX4023, MaokangBio), following the manufacturer's instructions. The macrophages were then mixed with the cancer cells at a ratio of 2:1 and seeded onto a 6‐well plate. Images were taken at the indicated time points, and the phagocytic index was calculated as the number of phagocytosed cancer cells per 100 macrophages. For anti‐CD47 antibody treatment, 10 µg mL^−1^ anti‐CD47 antibody or isotype IgG was added to the co‐culture medium of CHX2000 cells and iBMDM cells at a 1:1 cell ratio. Cancer cell confluence was determined by fluorescent imaging and quantification.

### Fluorescence‐Activated Cell Sorting for RNA‐seq Analysis

Primary human PDAC cells (1 million mL^−1^) were dissociated using TrypLE and fixed/permeabilized with 0.1% formaldehyde (#28906, Thermo Fisher) and 0.1 U/µL Recombinant RNase Inhibitor (#2313, Takara) for 5 minutes. The fixation was stopped by adding 56.1 µL of 2.5 M glycine, 50 µL of 1 M Tris‐HCl (pH 8.0), and 13.3 µL of 7.5% BSA per 1 mL cells on ice. After two washes, cells were stained with the anti‐GSDMC antibody for 15 minutes and counterstained with DAPI. Cells were sorted by using MoFlo Astrios (Beckman Coulter Life Sciences) for GSDMC‐positive and ‐negative cells (at least 1 million cells for each population). Cells were lysed in TRIzol and sent to BaiMu Bio (Shanghai, China) for RNA extraction, library preparation, sequencing, and analysis. Differentially expressed pathways were analyzed by using GSEA.

### Flow Cytometry Analysis

Primary human PDAC cells or murine KPC cells were dissociated using TrypLE (#12604013, Thermo Fisher) and incubated with FcR blocking reagent (Miltenyi). The cells were then stained with the indicated antibodies and appropriate isotype‐matched control antibodies, as listed in Table S1 (Supporting Information), following the manufacturer's instructions. DAPI (eBiosciences) was used for exclusion of dead cells. Samples were processed using the Attune NxT Flow Cytometer (Invitrogen) and analyzed using FlowJo V10 software.

### In Vivo Tumorigenicity Assay

PDAC cells were dissociated with trypsin, suspended in 20 µL Matrigel (#356234, Corning), and implanted subcutaneously into female 6–8 weeks old BALB/c nude mice. Tumor growth was monitored for up to 6 weeks after implantation. Procedures were conducted in accordance with the animals in science regulations (Shanghai Jiao Tong University Project Approval A‐2020‐004).

### In Vivo PDAC Models

Human PDAC cells (10^5^ in 20 µL Matrigel) were injected orthotopically into the pancreas of 6‐8‐week‐old BALB/c nude mice, while murine KPC cells (500 or 5,000 in 20 µL Matrigel) were injected orthotopically into the pancreas or subcutaneously into the flanks of 6‐8‐week‐old C57BL/6 mice, as described previously.^[^ ^8^, ^42^, ^46^ ^]^ For the metastasis model, cells were injected into the spleen and allowed to grow for one month before being harvested. All animal procedures were conducted in accordance with the 3Rs and the regulations for animals in science (Shanghai Jiao Tong University Project Approval A‐2020‐004).

### In Vivo Delivery of siRNA

The siRNA was mixed with Entranster in vivo transfection reagent (#18668111, Engreen) in an appropriate volume of saline, following the manufacturer's instructions. The mixture was injected intratumorally into subcutaneous tumors at the indicated dosage and frequency for certain experiments. Alternatively, for other experiments, the mixture was administered intraperitoneally.

### 10 × Genomics Single Cell RNAseq Analysis

Primary PDAC cells were treated with macrophage‐conditioned medium (MCM) for 48 hours. Cells were captured, barcoded and prepared for library according to the manufacturer's instructions (10 × Genomics). The samples were sequenced on NovaSeq 6000 S4 flow cell (Illumina). The Cell Ranger pipeline (v1.3) and Seurat R package were used to analyze sequencing data.^[^ ^45^ ^]^

### SMART Single‐Cell RNA‐Seq Analysis

Primary PDAC cells were treated with macrophage‐conditioned medium (MCM) for 48 hours and harvested by trypsinization. The single cells were sorted using cellenONE (Scienion, Germany) and then barcoded and prepared for library using SMART‐Seq v4 Ultra Low Input RNA Kit for Sequencing (#634891, Takara). The samples were sequenced on MGISEQ‐2000 at BGI (Wuhan, China). Gene expression data were normalized using the Min‐Max scaling method and grouped based on *GSDMC* expression level. Genes with *P* < 0.05 for comparisons between GSDMC^+^ and GSDMC^—^ cells were considered statistically significant. Selected genes were visualized in a heatmap using GraphPad Prism 9.0 software.

### TCGA Dataset and Gene Set Enrichment Analysis (GSEA)

Transcriptomic data for PDAC patients in The Cancer Genome Atlas Program (TCGA) database were downloaded from Genomic Data Commons Data Portal (https://portal.gdc.cancer.gov/). Patients were grouped based on the expression of GSDMC. GSEA tools (java GSEA Desktop Application version 4.2) were used to analyze key functional pathways enriched in different groups. A false discovery rate (FDR) < 0.05 was regarded as statistically significant. Gene Ontology (GO) and The Kyoto Encyclopedia of Genes and Genomes (KEGG) pathway analyses were performed using DAVID online tools (https://david.ncifcrf.gov/). Differentially expressed genes (upregulated or downregulated) were analyzed separately and *P* < 0.05 was considered as statistically significant.

### Bulk RNAseq Analysis

Murine KPC cells were lysed in Trizol (#15596026, Thermo), and the lysates were processed by Genergy BioTech (Shanghai, China) for library preparation and sequencing analysis. Differential expression analysis for genes across the different conditions was performed using DESeq2 with a threshold of |log2FC| ≥ 1 and a P‐value ≤ 0.05.

### Real‐Time Quantitative PCR Analysis

Total RNA was isolated using SteadyPure Quick RNA Extraction Kit (#AG21023, Agbio) according to the manufacturer's instructions. cDNA synthesis was performed using the Primescript RT Reagent Kit with gDNA Eraser (#RR047A, Takara Bio). qPCR analysis was conducted using the SuperReal PreMix Plus (SYBR Green #FP205, Tiangen) and analyzed with the QuantStudio 6 Pro System (Thermo). The change in mRNA was calculated using the ΔΔCt method. Primer sequences were provided in Table S2 (Supporting Information).

### Western blot Analysis

Tumor tissue or cultured cells were lysed in RIPA buffer (#89900, Thermo Fisher) supplemented with protease inhibitors (#11873580001, Sigma). The protein was resolved on 4–20% Surepage Bis‐Tris protein gels (#M00655, GenScript) and transferred to PVDF membranes (#ISEQ00010, Sigma). The membranes were blocked in Protein Free Rapid Blocking Buffer (#PS108, EpiZyme) for 10 minutes at RT, followed by an overnight incubation with primary antibodies at 4 °C. After that, the membranes were incubated with secondary antibodies at RT for 1 hour, and the blots were developed using Clarity Western ECL Substrate (#1605061, Bio‐Rad). Image J was used for band quantification.

### Cell Fractionation

Cells were lysed in fractionation buffer (20 mM HEPES, pH 7.4; 10 mM KCl; 2 mM MgCl2; 1 mM EDTA; 1 mM EGTA; 1 mM DTT; protease inhibitors) and incubated on ice for 15 minutes. The cell suspension was passed through a 27‐gauge needle for 10‐times, followed by centrifugation to pellet the nuclei. The supernatant was then centrifuged at 10,000 g to pellet organelles including the ER, Golgi apparatus, and mitochondria, while the remaining fraction was further centrifuged at 100,000 g to pellet the membrane fraction. All the pellets were washed once and lysed in TBS with 0.1% SDS before being processed for electrophoresis.

### Immunofluorescence

Cells were fixed in 4% paraformaldehyde for 10 minutes at RT, followed by permeabilization with 0.1% Triton X‐100 for 10 minutes at RT. Blocking was performed with 5% BSA for 1 hour at RT. Incubation with the indicated primary antibodies was carried out at 4 °C overnight, followed by incubation with fluorophore‐conjugated secondary antibodies for 1 hour at RT. DAPI (#Abs47047616, Absin) was used as a counterstain for the nuclei. Samples were mounted in Fluoroshield Mounting Medium (#Ab104135, Abcam), and images were captured using a fluorescent microscope or confocal microscope (LSM 880, Zeiss).

### Multiplexed Tissue Staining with CODEX

The process was conducted in accordance with instructions provided by the manufacturer (https://www.akoyabio.com/) and published methods.^[^ ^47^, ^48^ ^]^ A specially designed microfluidic system was employed for automated exchange of CODEX solutions and for capturing images (Akoya Biosciences, Menlo Park, CA, USA). Initially, square glass coverslips (22 × 22 mm, #72204‐01, Electron Microscopy Sciences) underwent a preparation process where they were coated with a poly‐L‐lysine solution (P8920, Sigma Aldrich) and then subjected to seven rinsing steps to eliminate any surplus solution. Subsequently, FFPE blocks were sliced onto these pre‐treated coverslips and kept at 4 °C until staining was performed. To broaden the range of antibodies available for CODEX assays, given the limited selection from Akoya, we conjugated antibodies with CODEX‐specific oligonucleotide barcodes. This was achieved by using third‐party, carrier‐free, and purified antibodies bound to CODEX‐specific oligonucleotide barcodes sourced from Akoya, resulting in antibodies ready for CODEX procedures. The conjugation process utilized the Antibody Conjugation Kit (#232195, Akoya Biosciences), with a minimum of 50 µg of antibody for each reaction and a 2:1 ratio of oligonucleotide to antibody by weight. Gel electrophoresis was conducted to confirm the success of the conjugation process. Among the antibodies conjugated were CD4 (#50134‐R001, monoclonal rabbit IgG clone #1, Sino Biological), CD8 (#ab230156, monoclonal rabbit clone [EPR21769], Abcam), Arginase1 (#NDP1‐32731, polyclonal rabbit, Novus Biologicals), and Nkp46 (#ab267792, monoclonal rabbit clone [EPR23097‐35], Abcam). In addition, commercially available conjugated antibodies such as panCK (#232180, Akoya Biosciences) and Ki67 (#232179, Akoya Biosciences) were also utilized in the assays.

For the staining of CODEX FFPE tissue, the CODEX Staining Kit (#232193, Akoya Biosciences) was used to accurately integrate fluorophores (reporters) into the oligonucleotide‐antibody conjugates. This process included baking the coverslip at 65 °C for 30 minutes, followed by deparaffinization, rehydration, and epitope retrieval under high pressure using a Tris‐EDTA Antigen Retrieval buffer solution with a pH of 9 (#abs9342, Absin). After a blocking step, the coverslip was stained with a panel of six‐marker antibodies overnight at 4 °C. Post staining, the coverslip was fixed using 1.6% paraformaldehyde, 100% methanol, and BS3 (#21580, Thermo Fisher), followed by three washes in 1X PBS. The prepared coverslips were then placed on bespoke CODEX acrylic plates (Bayview Plastic Solutions) for imaging. The imaging of the CODEX multicycle experiment was carried out with an inverted fluorescence microscope (Keyence, Osaka, Japan; Model BZ‐X710) equipped with a CFI Plan Apo λ 20×/0.75 objective (Nikon, Tokyo, Japan), a microfluidics instrument (Akoya Biosciences), and the CODEX driver software (Akoya Biosciences). A nuclear stain (#7000003, Akoya Biosciences) was applied in each cycle using channel 1, with the other antibodies imaged in their respective channels through an automated process using the following optimized exposure times:

**Table:** 

Target	Antibody clone	Supplier	Bar‐code	Fluoro‐phore	Dilu‐tion	Exposure time	Cycle	Channel
Nuclear stain	N/A	Akoya Biosciences	N/A	DAPI	1:300	10 ms	1–5	1
CD4	#1	Sino biological	BX002	ATTO550	1:200	350 ms	2	3
CD8	EPR21769	Abcam	BX015	Cy5	1:200	350 ms	2	4
Arginase1	polyclonal	Novus biologicals	BX027	Cy5	1:200	350 ms	3	4
Nkp46	EPR23097‐35	Abcam	BX030	Cy5	1:200	350 ms	4	4
Ki67	AKYP0052	Akoya Biosciences	BX047	ATTO550	1:200	350 ms	3	3
panCK	AKYP0053	Akoya Biosciences	BX019	AF750	1:200	250 ms	2	2

Subsequent steps, including hybridization, buffer exchange, image capture, and stripping, were executed automatically through the Akoya CODEX instrument manager (version 1.30.0.12). This device enabled the hybridization of fluorescent oligonucleotides within a hybridization buffer, the imaging of tissue in reporter stock solution, and the removal of fluorescent oligonucleotides using a stripping buffer. After completing the multi‐cycle process, hematoxylin and eosin (H&E) staining was applied manually and examined under a brightfield microscope setting.

### Tissue microarray (TMA) and Immunohistochemistry

TMA slides from patients at different PDAC stages (#HPanA060CD02) were purchased from Superchip Company (Shanghai). Other TMA samples were obtained from Ruijin Hospital with ethical approval and following informed consent (reference 2013‐0905‐70). IHC was performed using the SABC‐HRP Kit (#P0615, Beyotime) according to the manufacturer's instructions, and hematoxylin (#C0107, Beyotime) was used for counterstaining. Images were obtained with an Aperio Scanner System (Leica) at a magnification of 400×. The staining intensity in all sections was assessed using the H‐score by an experienced pathologist.

### Chromatin Immunoprecipitation Assay

ChIP was performed using Simplechip Plus Enzymatic Chromatin IP Kit (#9005, Cell Signaling Technology) following the manufacturer's instructions. The chromatin was incubated with the indicated antibodies (see Table S1, Supporting Information) at 4 °C overnight. Two types of antibodies were used to study GSDMC expression. One antibody (ABClonal, #A14550, Ab^Cyto^) primarily identified cytosolic GSDMC, therefore referred to as the GSDMC^Cyto^, representing full‐length GSDMC. A second antibody (ServiceBio, #GB111623, Ab^Nuc^) preferentially detected nuclear GSDMC, therefore referred to as the GSDMC^Nuc^. DNA was then eluted, reverse cross‐linked, and extracted for either DNA sequencing or qPCR analysis. The primer sequences were provided in Table S2 (Supporting Information). Data were available under the GEO Accession Number GSE243462.

### Lentivirus Production and Transduction

The shRNA sequences targeting human *GSDMC* and murine *Gsdmc* were provided in Table S3 (Supporting Information). The vectors for shRNA and inducible overexpression of GSDMC were purchased from VectorBuilder (China). The murine *Gsdmc* inducible knockdown plasmids were synthesized by Weichuang BioTech (China). Third‐generation lentiviruses were generated in 293T cells using the respective lentiviral backbone, psPAX2 packaging plasmid, and pMD2.g with PEI transfection reagent (#23966‐1, Polysciences). The viral particles were incubated with the target cells for 36 hours, then removed from cell culture, and selective antibiotics were added 24 hours later. The knockdown or overexpression efficiency was validated using qPCR or Western blotting.

### Inducible Knockdown and Overexpression

For in vitro analysis, cells were treated with 100 or 500 ng/mL doxycycline (#631311, Clontech) for the indicated times. For the in vivo experiment, doxycycline was added to the drinking water at a concentration of 1 mg/mL supplemented with 1% sucrose.

### In Vitro Transfection of siRNA or Plasmids

Each well of a 24‐well plate was loaded with 5 × 10^4^ cells overnight. Lipofectamine 3000 Transfection Reagent (#L3000015, Thermo Fisher) was used to deliver siRNA at a final concentration of 10 nM. Cells were collected 48 hours post‐transfection for qPCR measurement. Human GSDMC full‐length (508 amino acids) or mutant GSDMC (Δ310‐331, Δ392‐413, Δ456‐477) constructs were synthesized by QEgene (Shanghai, China).

### ALDH Activity Measurement

The ALDH activity of murine KPC cells was measured using the ALDEFLUOR kit (#01700, STEMCELL Tech), and the manufacturer's instructions were strictly followed. Samples were processed using the Attune NxT Flow Cytometer (Invitrogen) and analyzed using FlowJo V10 software.

### Statistical Analyses

The results were presented as means ± standard deviation (SD), unless stated otherwise. Statistical comparisons and differences were primarily assessed using non‐parametric tests, including the Mann‐Whitney U test for pairwise comparisons or the Kruskal‐Wallis test for multiple comparisons. Where normal distribution of the data could be demonstrated by Kolmogorov‐Smirnov and Shapiro‐Wilk tests, parametric tests like the t‐test or ANOVA were applied. Significance was considered at p‐values of *p < 0.05, **p < 0.01, ***p < 0.001, and ****p < 0.0001. All statistical analyses were performed using GraphPad Prism 9.0 software or SPSS version 26.

### Ethics & Inclusion Statement

The research design and execution included the active involvement of local scientists, ensuring that diverse perspectives and expertise were appropriately considered for the study. The conducted research holds significant local relevance, addressing specific challenges within the local context and contributing to advancements in cancer research. Plans were already developed in place to share the benefits of this research, including findings, data, and resources, with the scientific community, healthcare professionals, and the public. While adhering to local regulations, ethical guidelines were ensured and followed throughout the study to uphold the welfare of both animals and human participants involved in the research process.

## Conflict of Interest

The authors declare no conflict of interest.

## Author Contributions

R.W., J.L., and A.A. contributed equally to this work. R.W. developed the study concept, and acquired, analyzed, interpreted in vitro and in vivo data (Figures 1, 2, 3, 4, 5, 6, 7, 8, 9), and prepared the figures. J.L. acquired, analyzed, and interpreted data, performed in vitro and in vivo experiments (Figures 1, 2, 4, 5), and assisted with the bioinformatic analysis. A.A. assisted with the analysis of the data, helped with the study design, and drafted the manuscript. K.J. performed in vivo experiments (Figures 4, 6, 8, 9). S.Tondi acquired and analyzed in vitro data (Figures 4, 6, 9). S.D. conducted the pathological evaluation (Figures. 4, 8). Q.Z. performed in vivo experiments (Figure 9) and assisted with the bioinformatic analysis. S.Tang acquired and analyzed in vitro data (Figures 3 and 5) and helped with the study design. M.C. and Z.G. assisted with the bioinformatic analysis. B.Sabanovic acquired and analyzed in vitro data (Figure 4). P.A. acquired and analyzed in vitro data (Figure 4). L.J. provided valuable clinical samples from patients following informed consent (all experiments). A.S. conducted the pathological evaluation (Figures 1, 4). C.W., D.F., and B.Shen provided valuable clinical samples from patients following informed consent (all experiments). C.H. developed the study concept, prepared the figures, obtained funding, interpreted the data, and wrote the manuscript.

## Supporting information

Supporting Information

## Data Availability

The data that support the findings of this study are available on request from the corresponding author. The data are not publicly available due to privacy or ethical restrictions.
