# Supplementary material for: Gasdermin C promotes Stemness and Immune Evasion in Pancreatic Cancer via Pyroptosis‐Independent Mechanism
Source: Adv Sci (Weinh). 2024 Sep 19;11(42):2308990. doi: 10.1002/advs.202308990 (PMC11558074; doi:10.1002/advs.202308990)
Supplement: Supplementary file 1 — Supporting Information [file ADVS-11-2308990-s001.pdf]

ADVANCED SCIENCE
Open Access

Supporting Information

for *Adv. Sci.*, DOI 10.1002/advs.202308990

Gasdermin C promotes Stemness and Immune Evasion in Pancreatic Cancer via Pyroptosis-Independent Mechanism

*Renfei Wu, Jingwei Li, Alexandra Aicher, Ke Jiang, Serena Tondi, Shuang Dong, Quan Zheng, Siqi Tang, Minchun Chen, Zhenyang Guo, Berina Šabanović, Preeta Ananthanarayanan, Lingxi Jiang, Anna Sapino, Chenlei Wen, Da Fu, Baiyong Shen and Christopher Heeschen**

Gasdermin C promotes stemness and immune evasion in pancreatic cancer via pyroptosis-independent mechanism

Renfei Wu, Jingwei Li, Alexandra Aicher, Ke Jiang, Serena Tondi, Shuang Dong, Quan Zheng, Siqi Tang, Minchun Chen, Zhenyang Guo, Berina Šabanović, Preeta Ananthanarayanan, Lingxi Jiang, Anna Sapino, Chenlei Wen, Da Fu, Baiyong Shen, Christopher Heeschen

Supplementary information

Figure S1 – GSDMC is overexpressed in advanced PDAC

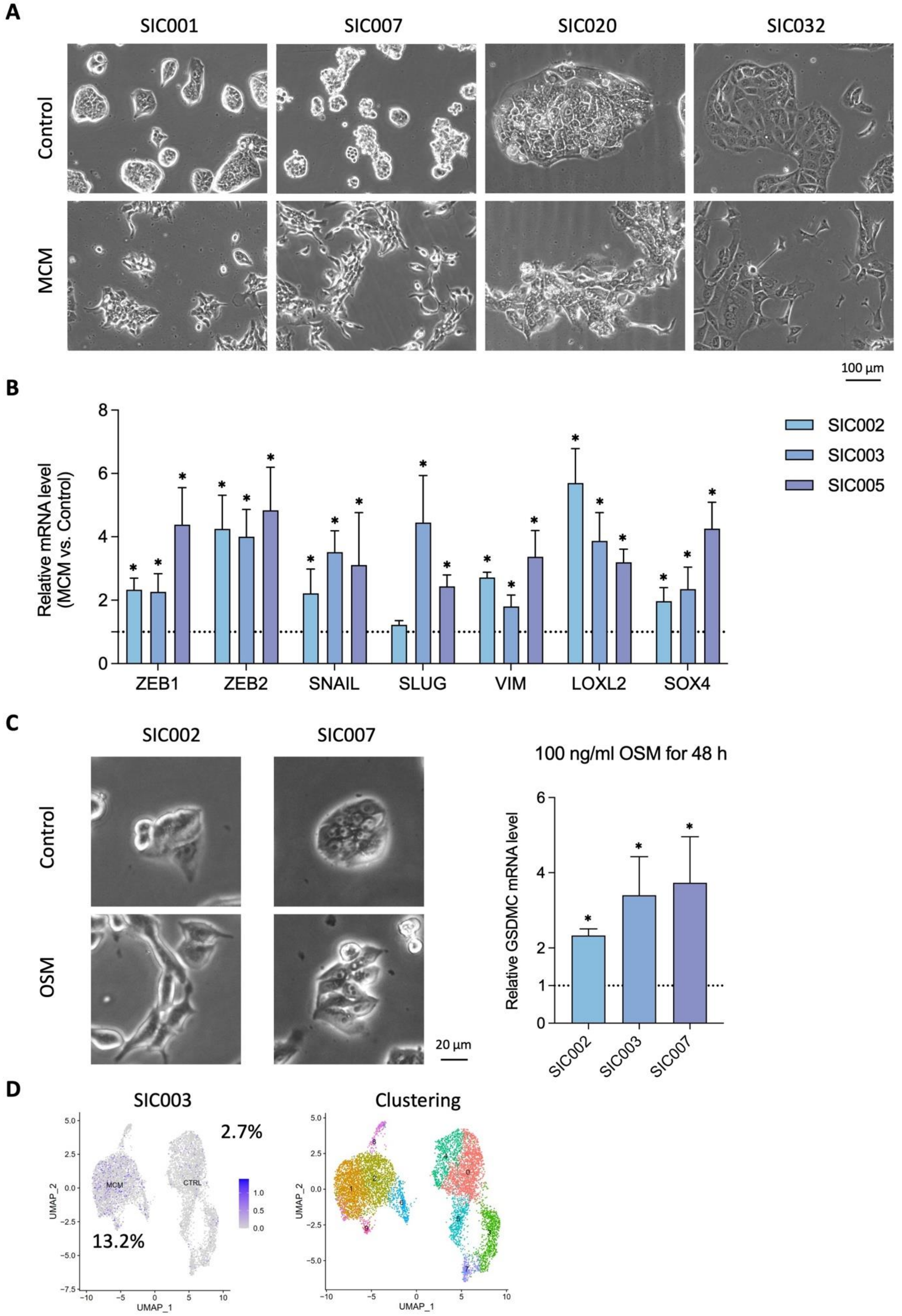

E

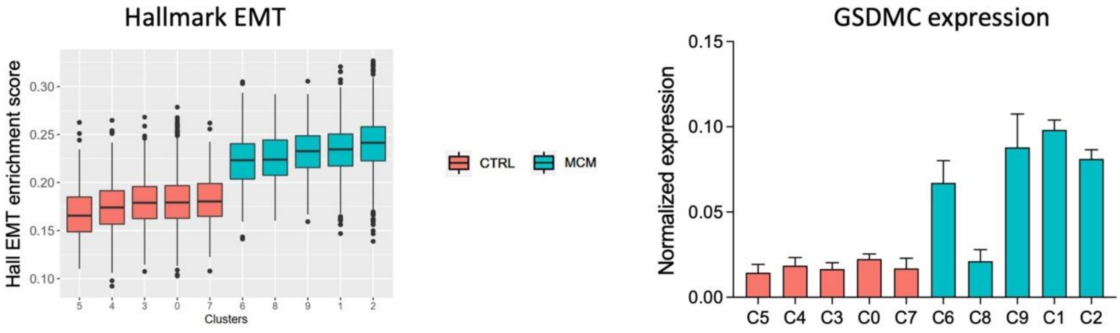
Hallmark EMT
Hall EMT enrichment score
0.30
0.25
0.20
0.15
0.10
5 4 3 0 7 6 8 9 1 2
Clusters
CTRL
MCM
GSDMC expression
Normalized expression
0.15
0.10
0.05
0.00
C5 C4 C3 C0 C7 C6 C8 C9 C1 C2

F

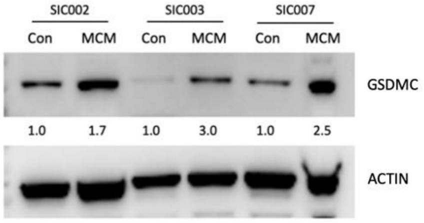
SIC002
SIC003
SIC007
Con MCM Con MCM Con MCM
GSDMC
1.0 1.7 1.0 3.0 1.0 2.5
ACTIN

G

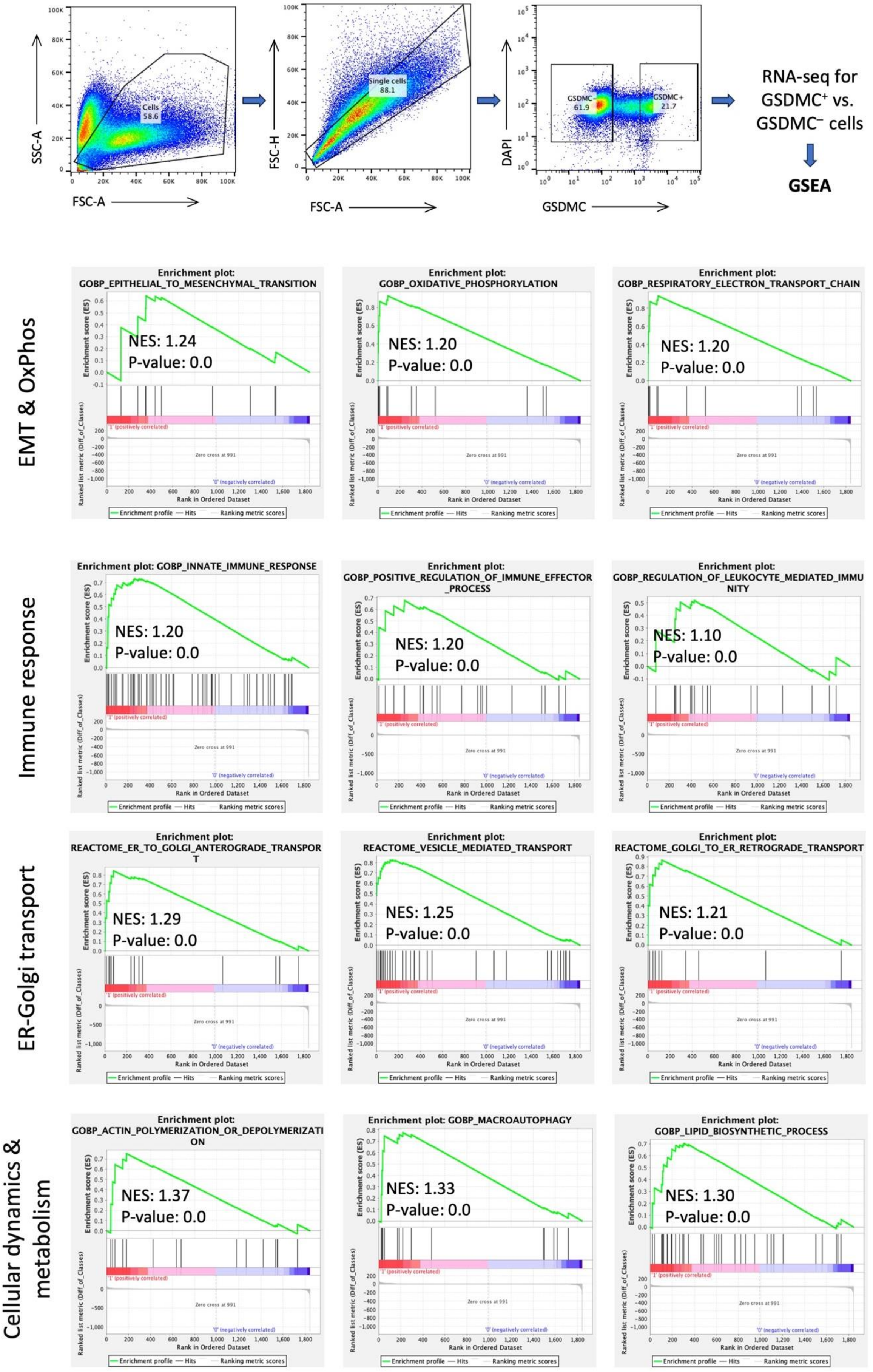
SSC-A
FSC-A
Cells 58.6
FSC-H
FSC-A
Single cells 88.1
DAPI
GSDMC
GSDMC− 61.9
GSDMC+ 21.7
RNA-seq for GSDMC⁺ vs. GSDMC⁻ cells
GSEA
EMT & OxPhos
Enrichment plot: GOBP_EPITHELIAL_TO_MESENCHYMAL_TRANSITION
NES: 1.24
P-value: 0.0
Enrichment plot: GOBP_OXIDATIVE_PHOSPHORYLATION
NES: 1.20
P-value: 0.0
Enrichment plot: GOBP_RESPIRATORY_ELECTRON_TRANSPORT_CHAIN
NES: 1.20
P-value: 0.0
Immune response
Enrichment plot: GOBP_INNATE_IMMUNE_RESPONSE
NES: 1.20
P-value: 0.0
Enrichment plot: GOBP_POSITIVE_REGULATION_OF_IMMUNE_EFFECTOR_PROCESS
NES: 1.20
P-value: 0.0
Enrichment plot: GOBP_REGULATION_OF_LEUKOCYTE_MEDIATED_IMMUNITY
NES: 1.10
P-value: 0.0
ER-Golgi transport
Enrichment plot: REACTOME_ER_TO_GOLGI_ANTEROGRADE_TRANSPORT
NES: 1.29
P-value: 0.0
Enrichment plot: REACTOME_VESICLE_MEDIATED_TRANSPORT
NES: 1.25
P-value: 0.0
Enrichment plot: REACTOME_GOLGI_TO_ER_RETROGRADE_TRANSPORT
NES: 1.21
P-value: 0.0
Cellular dynamics & metabolism
Enrichment plot: GOBP_ACTIN_POLYMERIZATION_OR_DEPOLYMERIZATION
NES: 1.37
P-value: 0.0
Enrichment plot: GOBP_MACROAUTOPHAGY
NES: 1.33
P-value: 0.0
Enrichment plot: GOBP_LIPID_BIOSYNTHETIC_PROCESS
NES: 1.30
P-value: 0.0
Enrichment score (ES)
Ranked list metric (Diff_of_Classes)
Rank in Ordered Dataset
Enrichment profile
Hits
Ranking metric scores

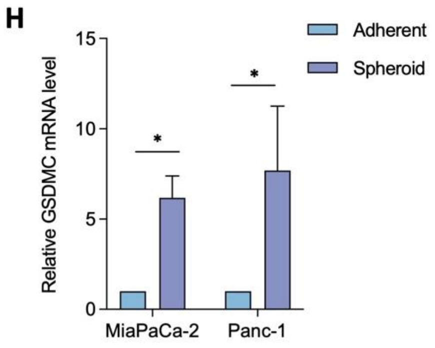
H
Adherent
Spheroid
Relative GSDMC mRNA level
15
10
5
0
*
*
MiaPaCa-2
Panc-1

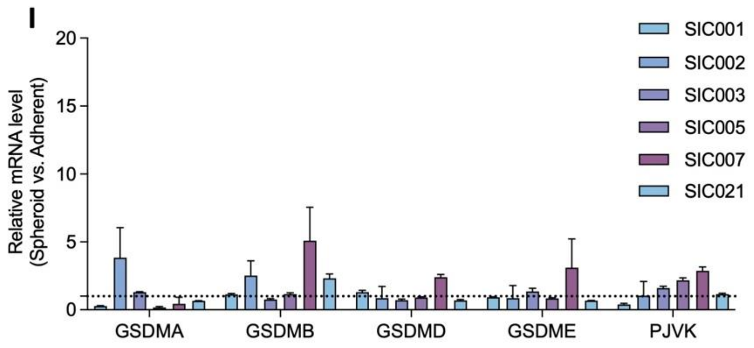
I
SIC001
SIC002
SIC003
SIC005
SIC007
SIC021
Relative mRNA level
(Spheroid vs. Adherent)
20
15
10
5
0
GSDMA
GSDMB
GSDMD
GSDME
PJVK

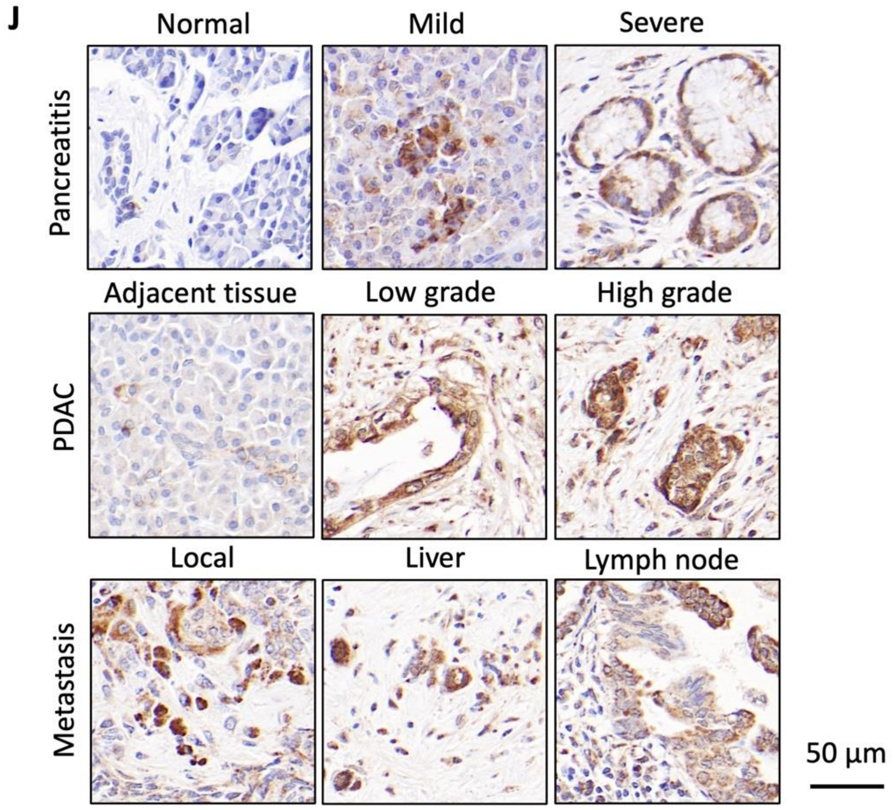
J
Normal
Mild
Severe
Pancreatitis
Adjacent tissue
Low grade
High grade
PDAC
Local
Liver
Lymph node
Metastasis
50 µm

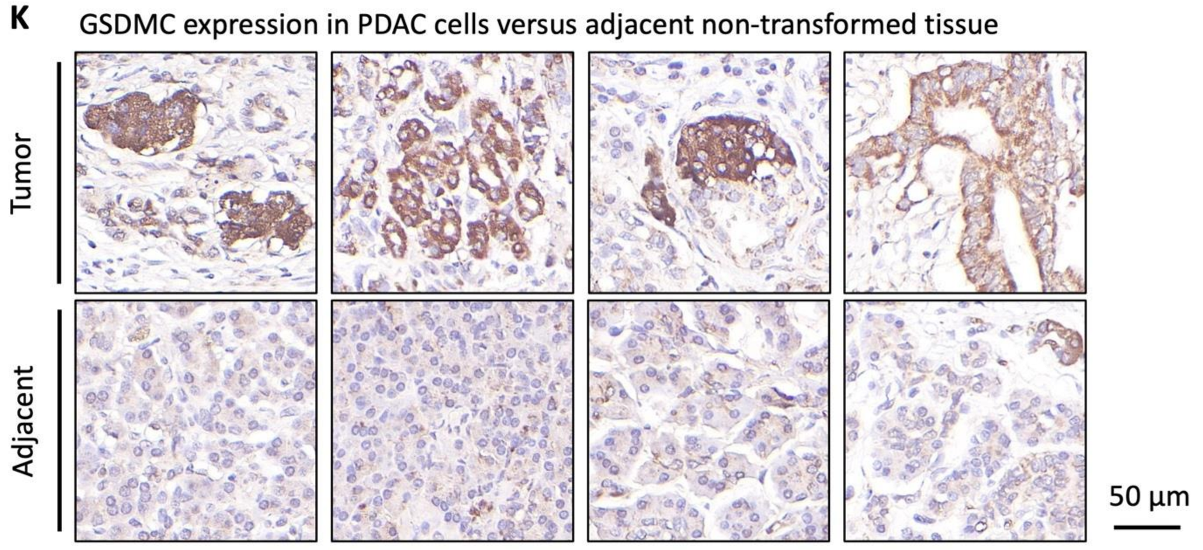
K
GSDMC expression in PDAC cells versus adjacent non-transformed tissue
Tumor
Adjacent
50 µm

L GSDMC expression in PDAC lesions from 14 patients

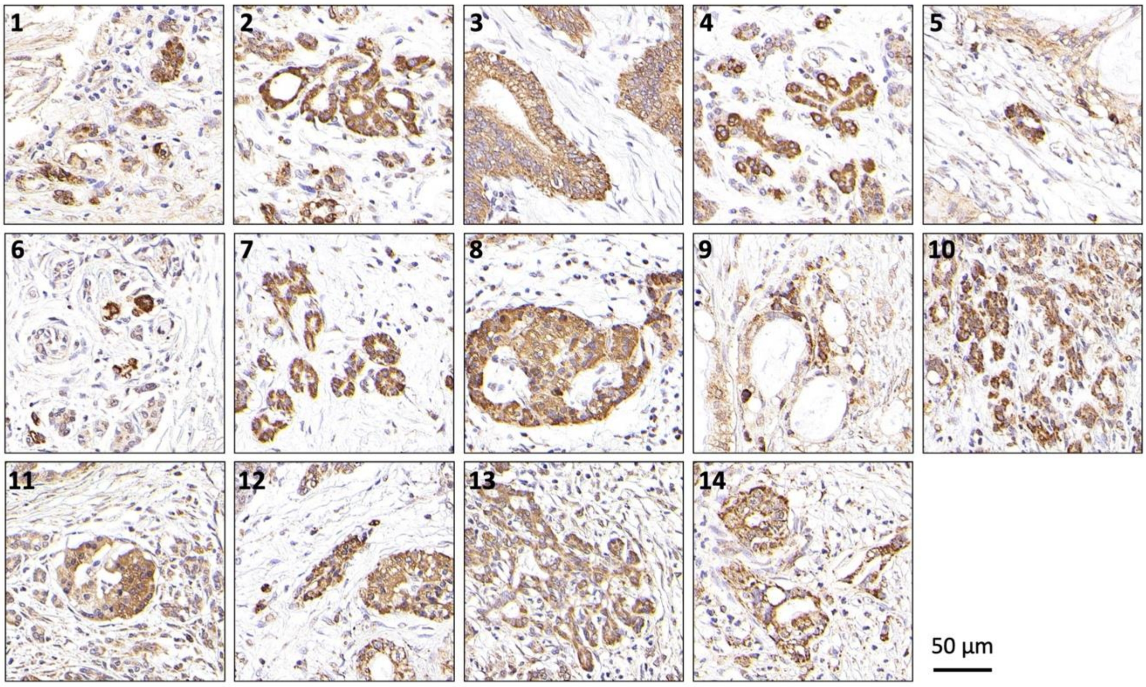

Pathological grade	Patient #	Tumor size (cm)	T stage	N stage	AJCC	# of positive lymph node	Invasion
Moderately differentiated	1	2.2*2*2	T3	N0	IIa	0	Duodenum, adipose
	2	4*2*1.2	T3	N0	IIa	0	Duodenum, bile duct, muscle
	3	3*2*2	T3	N0	IIa	0	Duodenum, adipose
Poorly differentiated	4	3*3*2.5	T3	N0	IIa	0	Duodenum, bile duct, adipose
	5	3*1.8*1.5	T3	N0	IIa	0	Duodenum, adipose
	6	2*2*1	T3	N0	IIa	0	Adipose
	7	2*2*2	T3	N0	IIa	3/28	Adipose
	8	5.5*4*3	T3	N1	IIb	2/37	Duodenum, bile duct, adipose
	9	3*2.8*2.1	T3	N1	IIb	6/40	Adipose
	10	3*2*1	T3	N1	IIb	5/13	Duodenum, adipose
	11	3*2*2	T3	N1	IIb	10/39	Duodenum
	12	3.7*2.8*2	T4	N1	III	1/5	Duodenum, bile duct
Undifferentiated	13	3*2*2	T3	N0	IIa	0	Bile duct
	14	3.5*2.5*2	T3	N1	IIb	6/17	Adipose

M

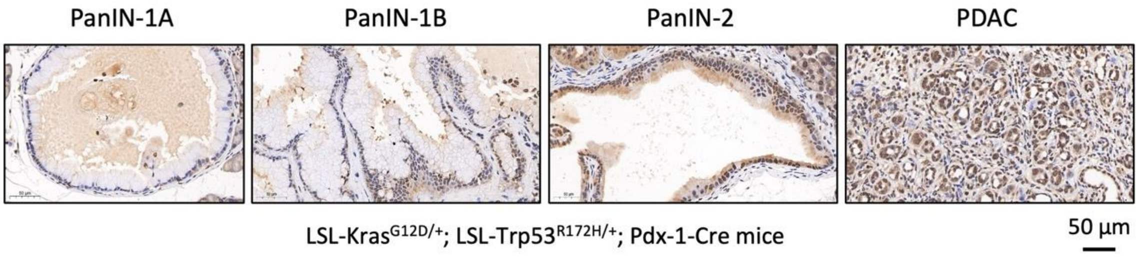

LSL-Kras$^{G12D/+}$; LSL-Trp53$^{R172H/+}$; Pdx-1-Cre mice

N

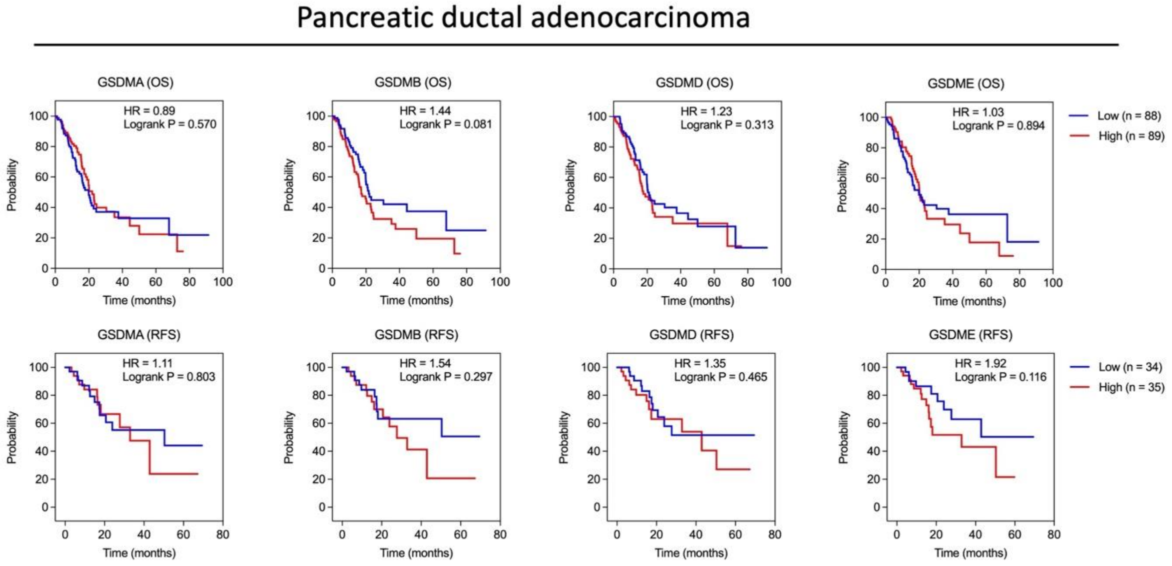

O

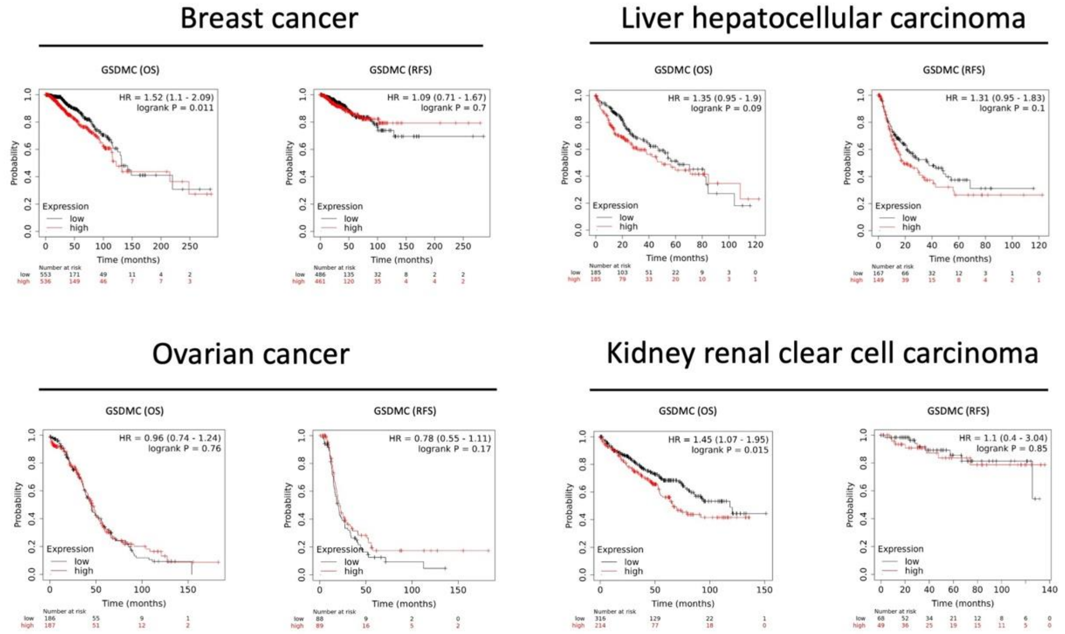

Figure S1 – GSDMC is overexpressed in advanced PDAC. (**A**) Representative images showing the morphology of primary human PDAC cultures SIC001, SIC007, SIC020, and SIC032 following treatment with macrophage-conditioned medium (MCM) versus control medium. (**B**) Fold change of EMT-related genes in three distinct human primary PDAC cell cultures following a 48-hour exposure to EMT-inducing MCM medium. Gene expression without MCM treatment is indicated as a dotted line (n=3 independent samples). (**C**) Representative morphology of the human PDAC primary cultures SIC002 and SIC007 with and without Oncostatin (OSM) treatment (**left panel**), and quantification of *GSDMC* mRNA expression (**right panel**). Gene expression without OSM treatment is indicated as a dotted line (n=3 independent samples). (**D**) UMAP projection of single-cell data illustrating the percentage of GSDMC$^+$ cancer cells within primary PDAC cultures (SIC003) after pre-treatment with EMT-inducing

MCM or control medium (**left panel**). UMAP projection focusing specifically on clusters 0-8 (**right panel**). (**E**) Hallmark EMT enrichment score for clusters 0-8 in PDAC cultures treated with MCM or control medium (**left panel**), and *GSDMC* expression levels for clusters 0-8 (**right panel**). (**F**) GSDMC protein levels in three different PDAC models, following MCM treatment for 48 hours. (**G**) Gene Set Enrichment plots were generated by comparing $GSDMC^{+}$ versus $GSDMC^{-}$ cancer cells. For this purpose, human PDAC cells (SIC003) were fixed, permeabilized, and stained with the GSDMC antibody (ServiceBio GB111623), followed by fluorescence-activated cell sorting. More than a million of $GSDMC^{+}$ and $GSDMC^{-}$ cells were collected, lysed in TRIzol reagent, and prepared for bulk RNA sequencing. Enriched pathways related to the regulation of immune response, ER-Golgi transport, epithelial-mesenchymal transition (EMT), oxidative phosphorylation, cytoskeleton organization, cellular degradation, and lipid metabolism are depicted. The normalized enrichment scores (NES) and corresponding p-values are shown. (**H**) Fold change for *GSDMC* expression in CSC-enriched spheres versus adherent cells, for two human PDAC cell lines (n=3 independent samples). (**I**) Fold change for *GSDMA*, *GSDMB*, *GSDMD*, *GSDME*, and *PJVK* expression in CSC-enriched spheres versus adherent cells (Adh, dotted line) for six human primary PDAC cell cultures (n=3 independent samples). (**J**) Immunohistochemistry (IHC) depicting the expression of GSDMC in the following human tissue samples: pancreatitis of varying severity levels, local pancreatic cancer (low or high grade), and advanced pancreatic cancer, including cases where the cancer has spread to lymph nodes and the liver. (**K**) IHC illustrating the expression of GSDMC in human PDAC cells compared to the surrounding non-transformed tissue. (**L)** IHC for GSDMC expression in human tumor tissue samples obtained from a diverse set of 14 PDAC patients. The table below shows pathological grades and staging for each patient's tumor. (**M)** Expression of GSDMC in the pancreas of KPC mice (LSL-*Kras*$^{G12D/+}$; LSL-*Trp53*$^{R172H/+}$; Pdx-1-Cre). GSDMC staining in low-grade pancreatic lesions (PanIN-1A, Pan-IN-1B, and PanIN-2) towards fully developed PDAC is shown. (**N**) Analysis for overall survival (OS) and relapse-free survival (RFS) in PDAC patients based on the expression of *GSDMA*, *GSDMB*, *GSDMD*, and *GSDME* using the Kaplan-Meier Plotter (https://kmplot.com/analysis/). Patient samples were dichotomized at the median value of each target gene. (**O**) Overall survival (**left panels**) and relapse-free survival (**right panels**) for various types of cancer patients, dichotomized at the median value of *GSDMC* expression. * $p<0.05$; Mann-Whitney U test, two-tailed, unless otherwise stated.

Figure S2 – GSDMC is linked to invasion and stemness phenotypes

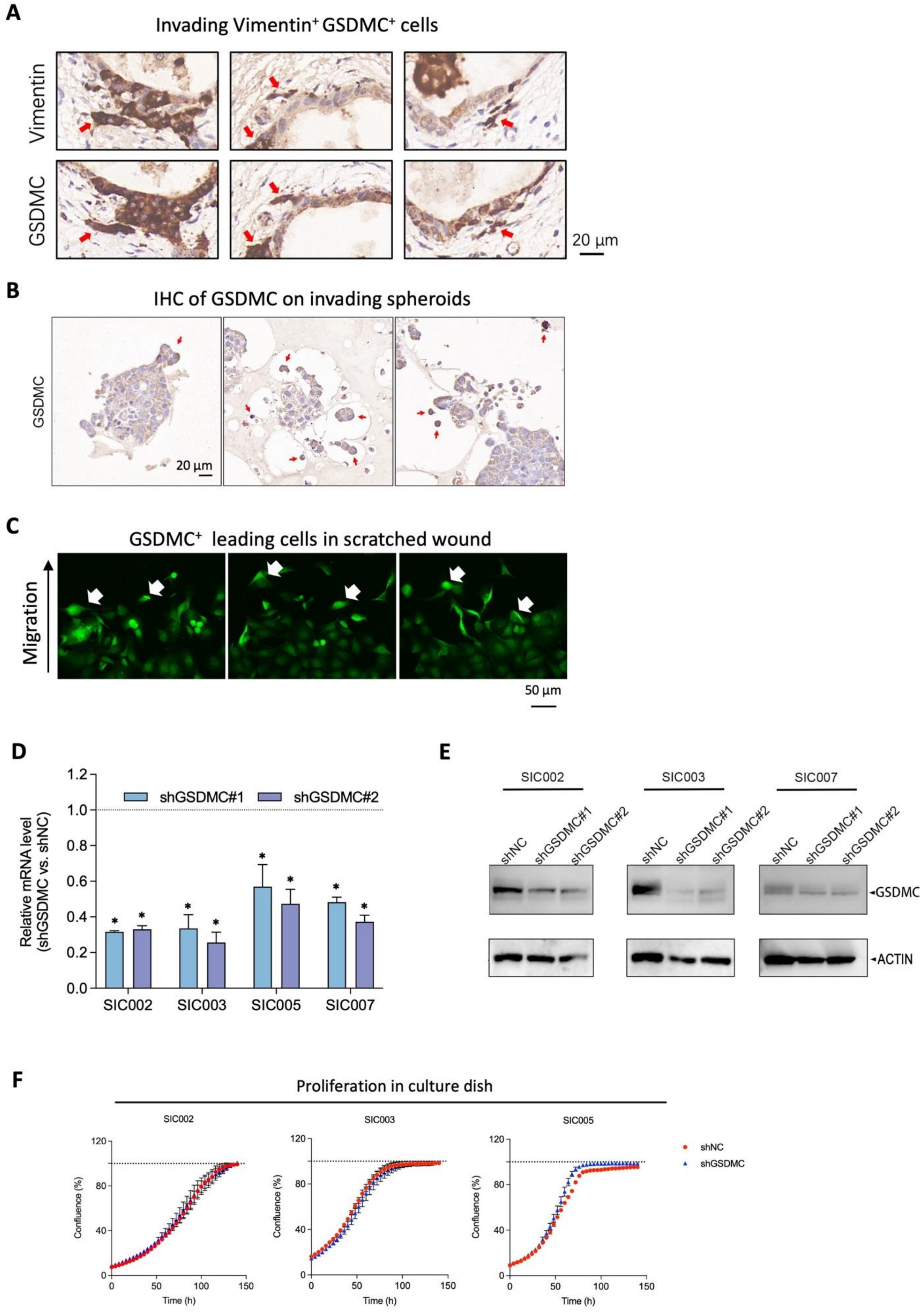

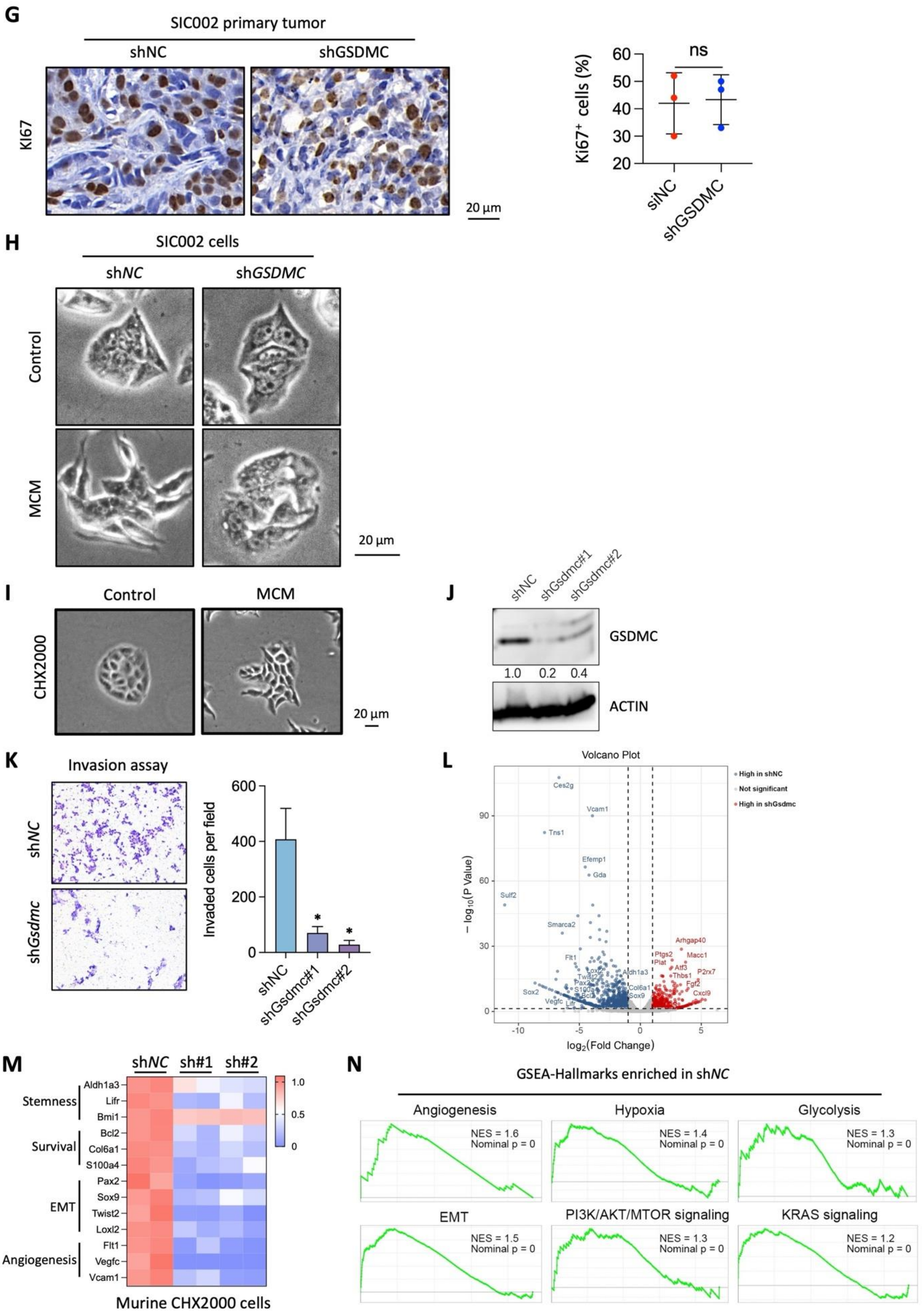

G
SIC002 primary tumor
shNC
shGSDMC
Ki67
20 μm
ns
Ki67+ cells (%)
siNC
shGSDMC
H
SIC002 cells
shNC
shGSDMC
Control
MCM
20 μm
I
Control
MCM
CHX2000
20 μm
J
shNC
shGsdmc#1
shGsdmc#2
GSDMC
1.0 0.2 0.4
ACTIN
K
Invasion assay
shNC
shGsdmc
Invaded cells per field
shNC
shGsdmc#1
shGsdmc#2
L
Volcano Plot
High in shNC
Not significant
High in shGsdmc
− log10(P Value)
log2(Fold Change)
M
shNC
sh#1
sh#2
Stemness
Survival
EMT
Angiogenesis
Aldh1a3
Lifr
Bmi1
Bcl2
Col6a1
S100a4
Pax2
Sox9
Twist2
Loxl2
Flt1
Vegfc
Vcam1
Murine CHX2000 cells
N
GSEA-Hallmarks enriched in shNC
Angiogenesis
NES = 1.6
Nominal p = 0
Hypoxia
NES = 1.4
Nominal p = 0
Glycolysis
NES = 1.3
Nominal p = 0
EMT
NES = 1.5
Nominal p = 0
PI3K/AKT/MTOR signaling
NES = 1.3
Nominal p = 0
KRAS signaling
NES = 1.2
Nominal p = 0

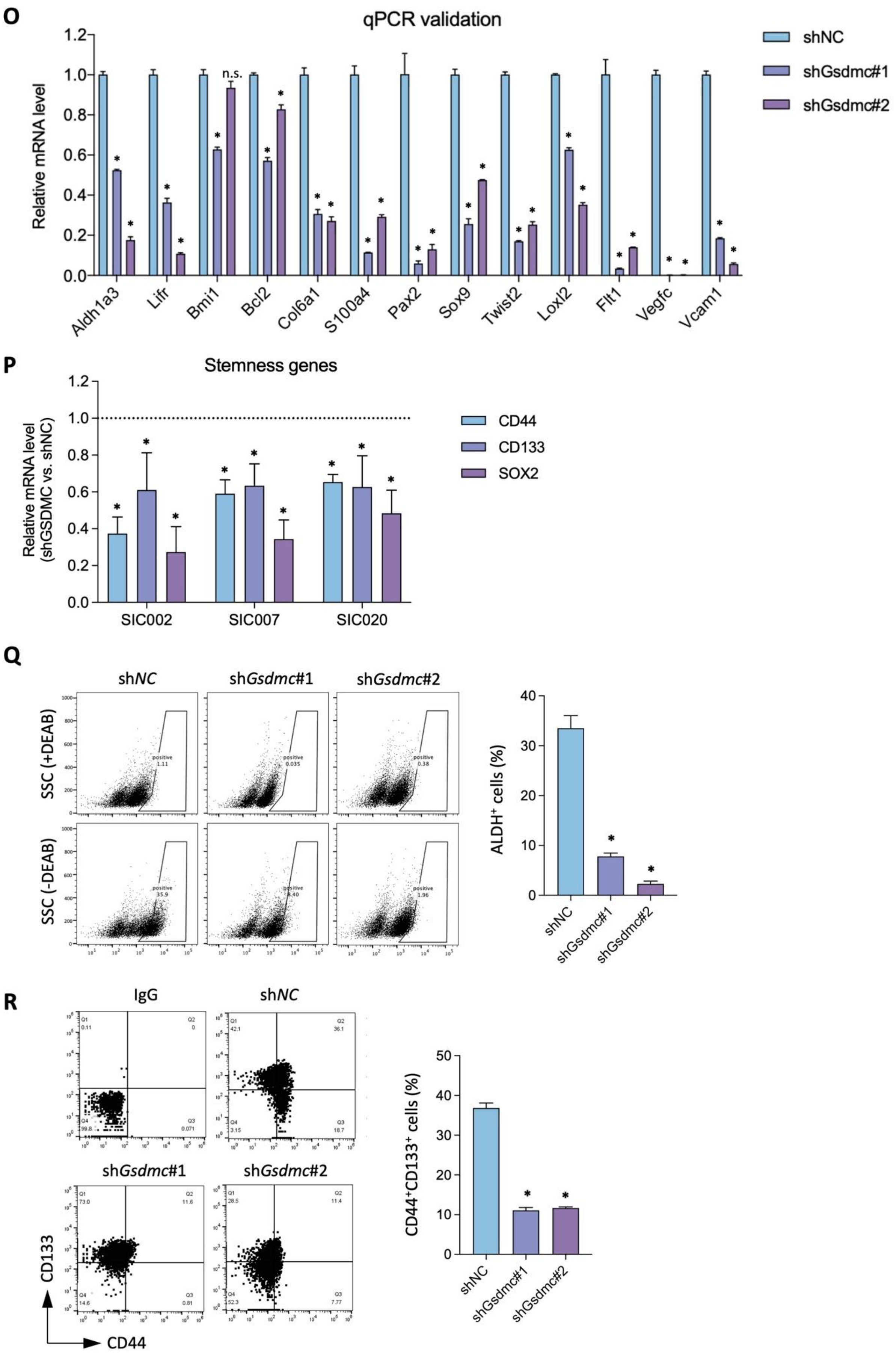
O
qPCR validation
Relative mRNA level
shNC
shGsdmc#1
shGsdmc#2
n.s.
Aldh1a3
Lifr
Bmi1
Bcl2
Col6a1
S100a4
Pax2
Sox9
Twist2
Loxl2
Flt1
Vegfc
Vcam1
P
Stemness genes
Relative mRNA level (shGSDMC vs. shNC)
CD44
CD133
SOX2
SIC002
SIC007
SIC020
Q
shNC
shGsdmc#1
shGsdmc#2
SSC (+DEAB)
SSC (-DEAB)
positive 1.11
positive 0.035
positive 0.38
positive 35.9
positive 1.96
ALDH+ cells (%)
R
IgG
shNC
shGsdmc#1
shGsdmc#2
CD133
CD44
CD44+CD133+ cells (%)

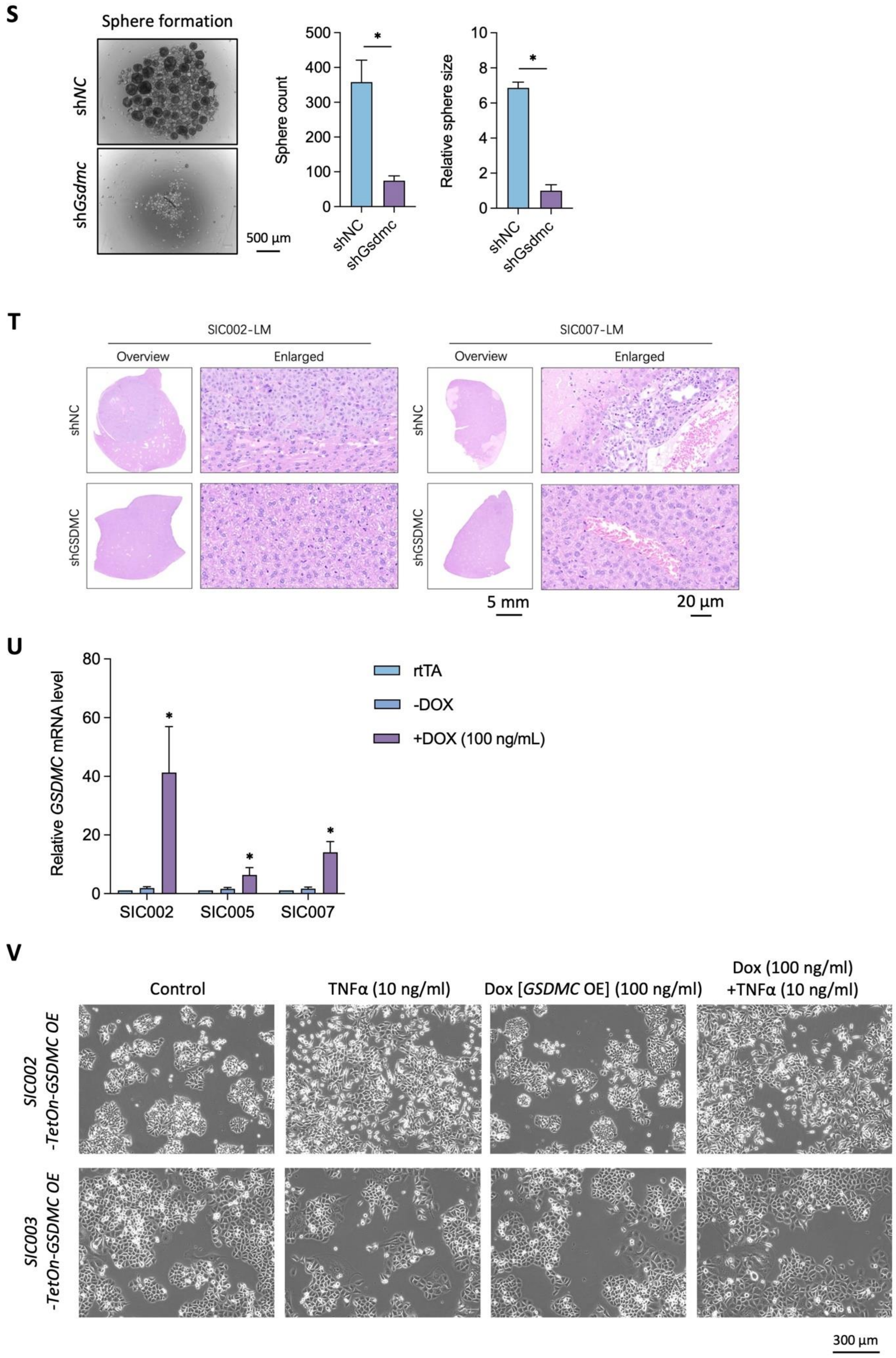
S
Sphere formation
shNC
shGsdmc
500 µm
Sphere count
*
500
400
300
200
100
0
shNC
shGsdmc
Relative sphere size
*
10
8
6
4
2
0
shNC
shGsdmc
T
SIC002-LM
SIC007-LM
Overview
Enlarged
Overview
Enlarged
shNC
shGSDMC
shNC
shGSDMC
5 mm
20 µm
U
Relative GSDMC mRNA level
80
60
40
20
0
SIC002
SIC005
SIC007
rtTA
-DOX
+DOX (100 ng/mL)
*
*
*
V
Control
TNFα (10 ng/ml)
Dox [GSDMC OE] (100 ng/ml)
Dox (100 ng/ml)
+TNFα (10 ng/ml)
SIC002
-TetOn-GSDMC OE
SIC003
-TetOn-GSDMC OE
300 µm

Figure S2 – GSDMC is linked to invasion and stemness phenotypes. (**A**) Immunohistochemistry (IHC) for GSDMC and VIMENTIN in human PDAC specimens, performed on consecutive sections. Arrows indicate invading cells that are positive for both GSDMC and vimentin. (**B**) IHC for GSDMC on invading spheres (arrows) of human PDAC cultures. (**C**) Immunofluorescence analysis for GSDMC illustrating the presence of GSDMC^{+} cells (arrows) at the leading edge of scratch wounds. (**D**) Fold change in *GSDMC* expression for four distinct PDAC models following *GSDMC* knockdown using two different sh*GSDMC* variants. The dotted line indicates *GSDMC* expression for control sh*NC* (n=3 independent samples). (**E**) GSDMC protein levels in three different PDAC models, following *GSDMC* knockdown using two different sh*GSDMC* variants as shown in (**D**). The ratio of GSDMC to β-ACTIN expression is indicated. (**F**) Expansion of three distinct PDAC cultures assessed as confluency using Incucyte™, a real-time live-cell imaging platform. (**G**) IHC for KI-67 in SIC002 PDAC tumors for sh*GSDMC* versus sh*NC* control. Representative images (**left**), quantification (**right**); n=3. (**H**) Visualization of cell morphology in PDAC cells following knockdown with sh*GSDMC* and sh*NC* control, in the absence or presence of MCM for 48 hours. (**I**) Morphological changes of murine KPC cultures (CHX2000) following MCM treatment for 48 hours. (**J**) Western blot analysis to show the knockdown efficiency of two different variants of sh*Gsdmc* versus sh*NC* in CHX2000 murine KPC-derived PDAC cultures. The ratio of GSDMC to β-ACTIN expression is indicated. (**K**) Invasion of murine primary PDAC cultures following knockdown with sh*Gsdmc* using two different sh*Gsdmc* variants versus sh*NC* control. Representative images showing invaded cells stained with crystal violet (**left panel**), and quantification of the invaded cells (**right panel**) (n=3 independent samples). (**L**) Volcano plot generated with OmicsVolcano V1.1 (https://github.com/IrinaVKuznetsova/OmicsVolcano) illustrating the fold change versus p-value for genes exhibiting elevated expression in murine KPC cultures (CHX2000 cells) upon knockdown of *Gsdmc* (depicted in red) versus overexpressed genes for control sh*NC* (shown in blue). (**M)** Heatmap illustrating the modulation of genes associated with stemness, cell survival, epithelial-mesenchymal transition (EMT), and angiogenesis for murine KPC cultures following *Gsdmc* knockdown using two distinct sh*Gsdmc* variants versus control sh*NC*. (**N**) Gene Set Enrichment Analysis (GSEA) for hallmarks associated with angiogenesis, hypoxia, glycolysis, EMT, PI3K signaling, and KRAS signaling in sh*NC*-treated samples. (**O**) Fold change for expression of genes related to stemness, survival, EMT, and angiogenesis (n=3 independent samples). (**P**) Fold change for the expression of *CD44*, *CD133*, and *SOX2* in three different human PDAC models. The dotted line represents the expression for control sh*NC* (n=3 independent samples). (**Q**) ALDEFLUOR flow cytometry for murine KPC cultures following knockdown of *Gsdmc* using two distinct sh*Gsdmc* variants versus control sh*NC*. Representative flow cytometry dot plots in the presence or absence of the ALDH1A1-specific inhibitor DEAB (N,N-Diethylaminobenzaldehyde) to inhibit ALDH activity (**left panel**). Quantification of ALDH^{+} cells (**right**

panel) (n=3 independent samples). (**R**) Flow cytometry for CD44 and CD133 in murine KPC cultures (CHX2000). Representative flow cytometry dot plots (**left panel**). Quantification of $CD44^{+}CD133^{+}$ cells (**right panel**) (n=3 independent samples). (**S**) Sphere formation capacity expressed as number of formed spheres per 10,000 cells in 1 ml following knockdown of *Gsdmc* compared to control sh*NC* in murine primary KPC cultures. Representative photographs (**left panel**), quantification of sphere counts per 10,000 cells in 1 ml (**middle panel**), and sphere size (**right panel**) (n=3 independent samples). (**T**) H&E staining of liver metastases from two primary pancreatic tumors (SIC002-LM and SIC007-LM) following knockdown for GSDMC (sh*GSDMC*) versus control (sh*NC*) provided as overview and at higher magnification. (**U**) *GSDMC* expression following doxycycline (DOX)-inducible knockdown of *GSDMC* in three different human PDAC models using reverse tetracycline-controlled transactivator (rtTA), a regulatory protein allowing for the control of gene expression in the presence of DOX. DOX treatment (+DOX) is compared with treatment without DOX (-DOX). (**V**) Treatment of PDAC cells with TNFα as a common inducer of pyroptosis did not result in enhanced cell death, even when GSDMC was overexpressed simultaneously. * $p<0.05$; Mann-Whitney U test, two-tailed.

Figure S3 – GSDMC is linked to immune evasion signals

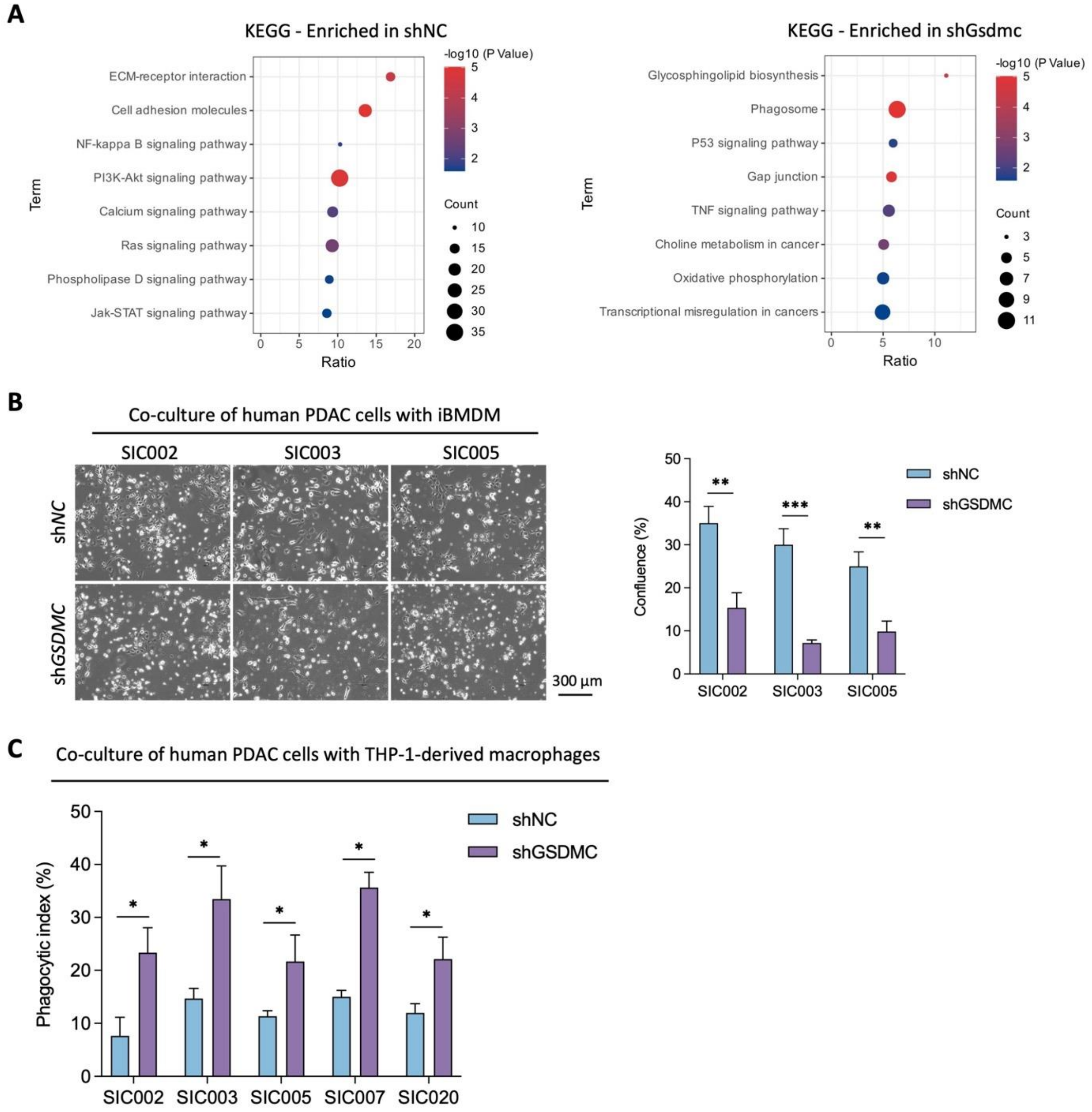

Figure S3 – *GSDMC is linked to immune evasion signals.* (A) Enriched KEGG pathways in PDAC primary cultures following knockdown of *Gsdmc* (sh*Gsdmc*) compared to the control (sh*NC*). (**B**) Co-culture of three distinct human PDAC models following knockdown of *GSDMC* (sh*GSDMC*) or control (sh*NC*) with immortalized Bone Marrow-Derived Macrophage (iBMDM) cells. Representative images demonstrating the co-culture setup (**left panel**) and quantification (**right panel**) (n=3 independent samples; two-tailed t-test). (**C**) Phagocytic index for co-culture of five different human PDAC models with knockdown of *GSDMC* (sh*GSDMC*) or control (sh*NC*) with THP-1-derived macrophages (n=3 independent samples; two-tailed Mann-Whitney U test). * $p<0.05$, ** $p<0.01$, and *** $p<0.001$.

Figure S4 – GSDMC promotes immune evasion in PDAC

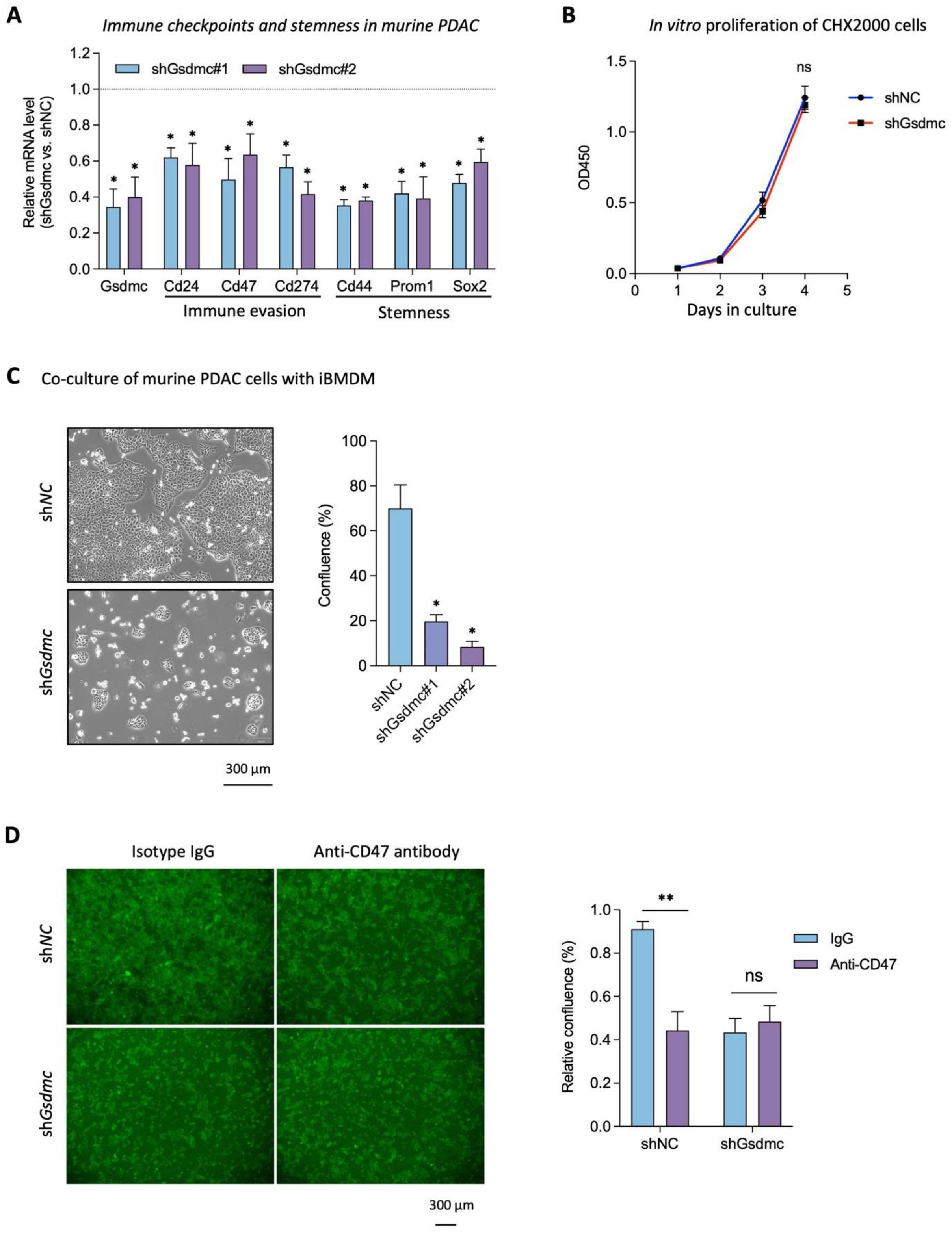

E

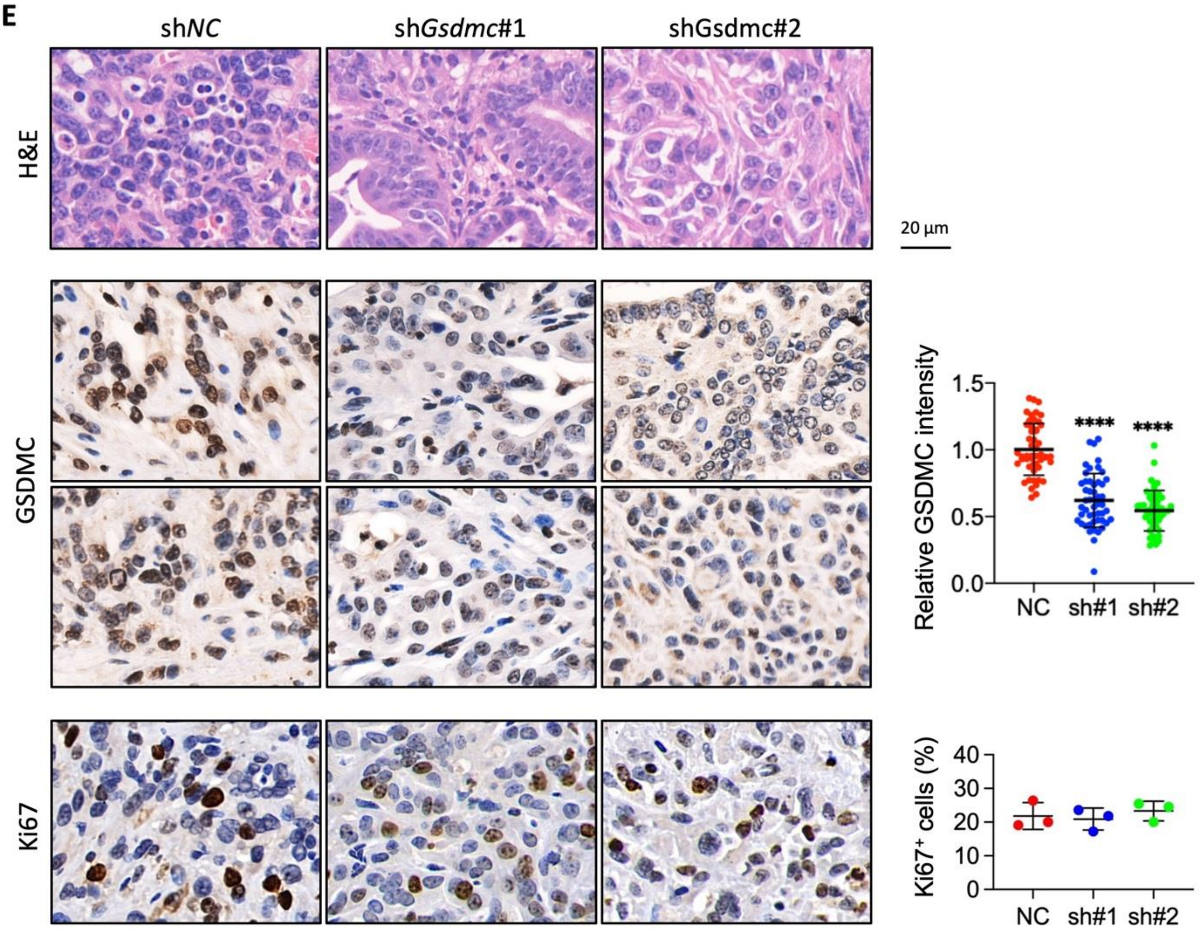

shNC
shGsdmc#1
shGsdmc#2
H&E
20 μm
GSDMC
Relative GSDMC intensity
1.5
1.0
0.5
0.0
****
****
NC
sh#1
sh#2
Ki67
Ki67+ cells (%)
40
30
20
10
0
NC
sh#1
sh#2

F

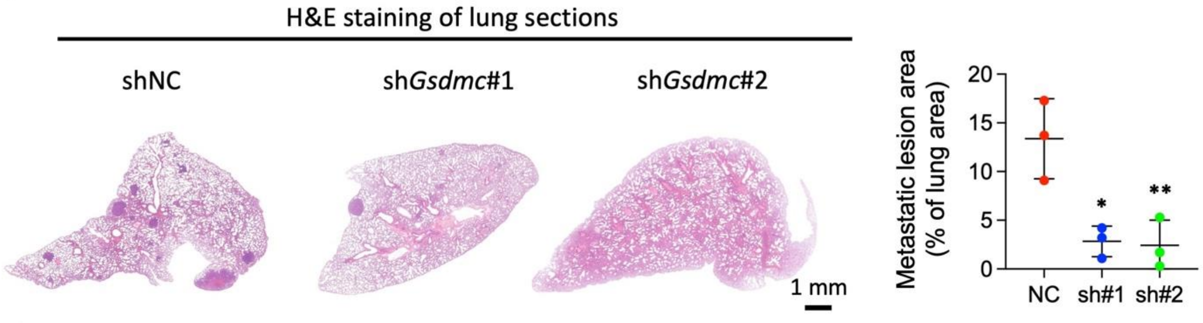

H&E staining of lung sections
shNC
shGsdmc#1
shGsdmc#2
1 mm
Metastatic lesion area
(% of lung area)
20
15
10
5
0
*
**
NC
sh#1
sh#2

G

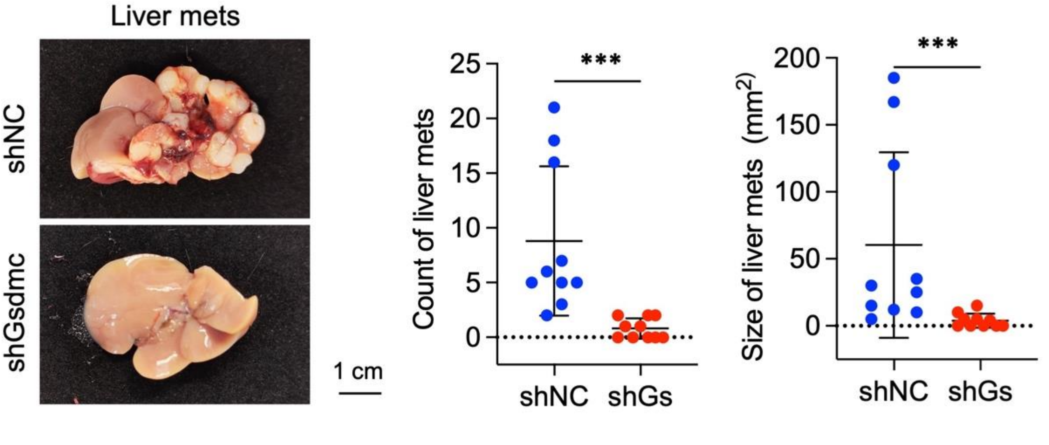

Liver mets
shNC
shGsdmc
1 cm
Count of liver mets
25
20
15
10
5
0
***
shNC
shGs
Size of liver mets (mm2)
200
150
100
50
0
***
shNC
shGs

H

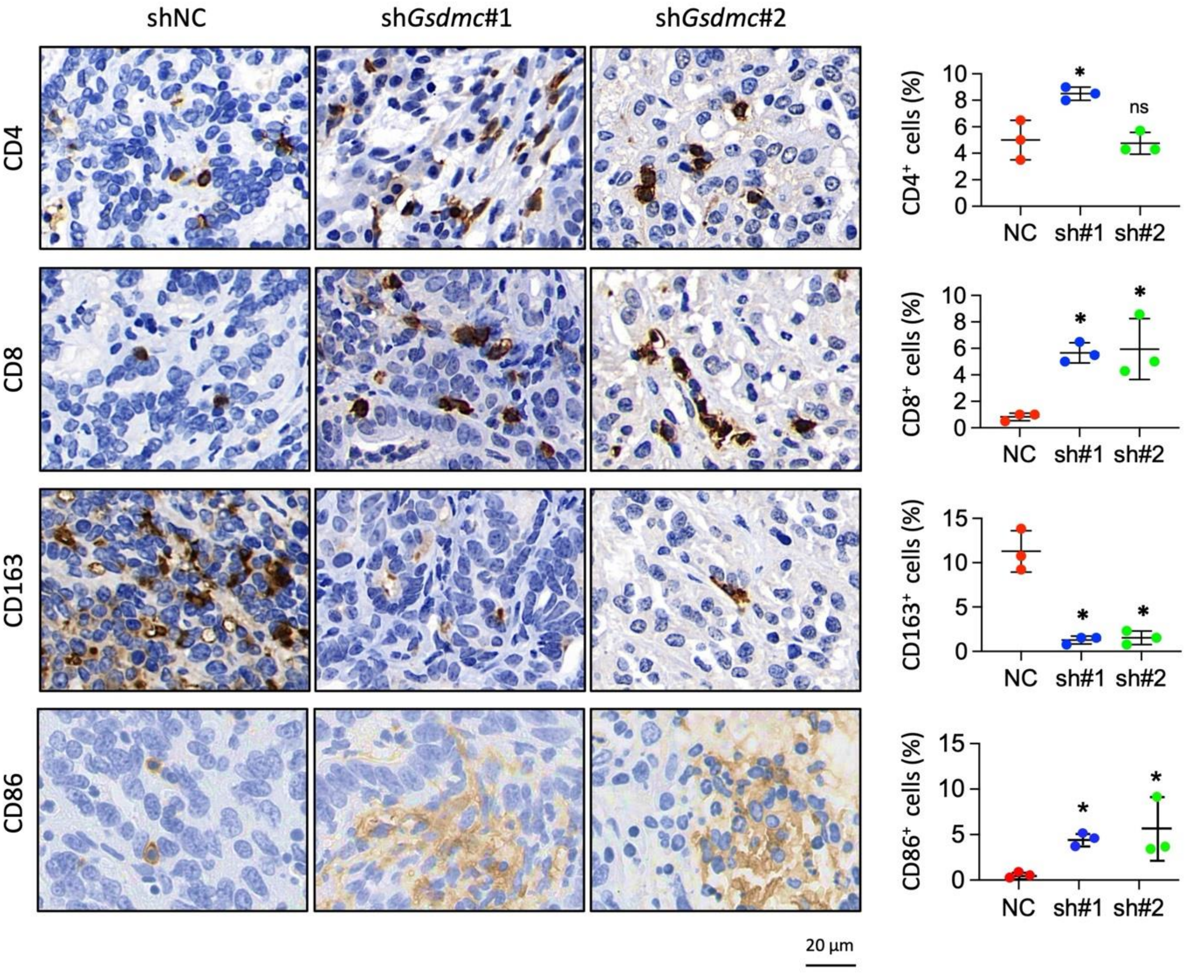

shNC
shGsdmc#1
shGsdmc#2
CD4
CD8
CD163
CD86
CD4+ cells (%)
CD8+ cells (%)
CD163+ cells (%)
CD86+ cells (%)
NC
sh#1
sh#2
ns
*
20 µm

I

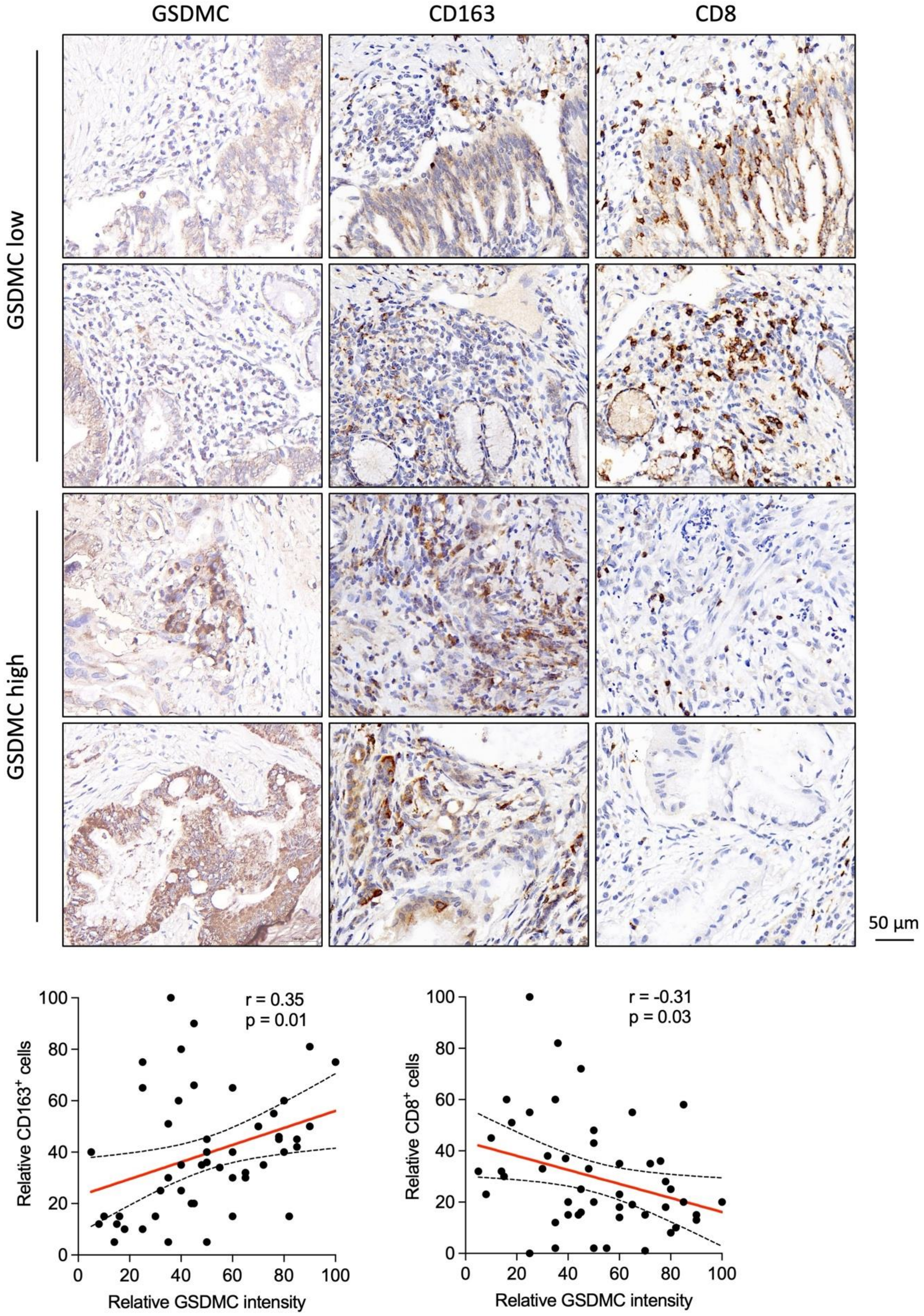
GSDMC
CD163
CD8
GSDMC low
GSDMC high
50 μm
r = 0.35
p = 0.01
Relative CD163$^+$ cells
Relative GSDMC intensity
r = -0.31
p = 0.03
Relative CD8$^+$ cells
Relative GSDMC intensity

J

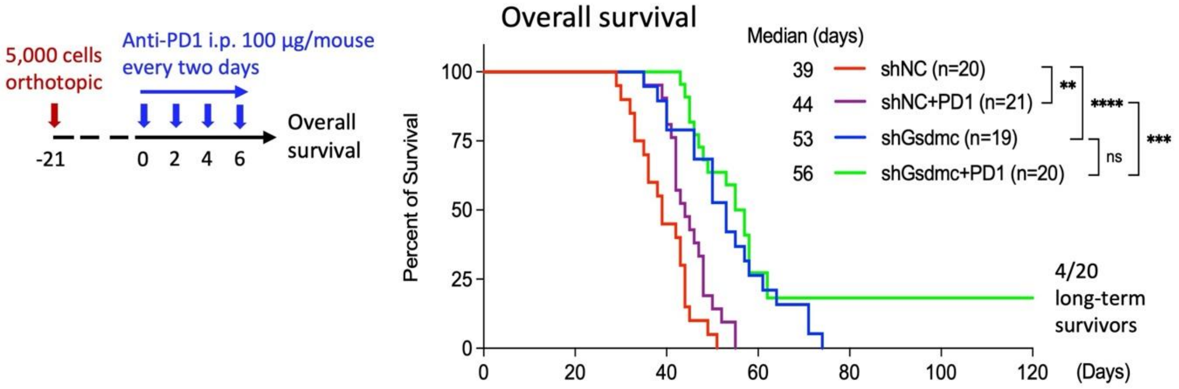
5,000 cells
orthotopic
Anti-PD1 i.p. 100 µg/mouse
every two days
-21
0 2 4 6
Overall
survival
Overall survival
Median (days)
39 shNC (n=20)
44 shNC+PD1 (n=21)
53 shGsdmc (n=19)
56 shGsdmc+PD1 (n=20)
**
****
ns
***
Percent of Survival
100
75
50
25
0
0 20 40 60 80 100 120 (Days)
4/20
long-term
survivors

K

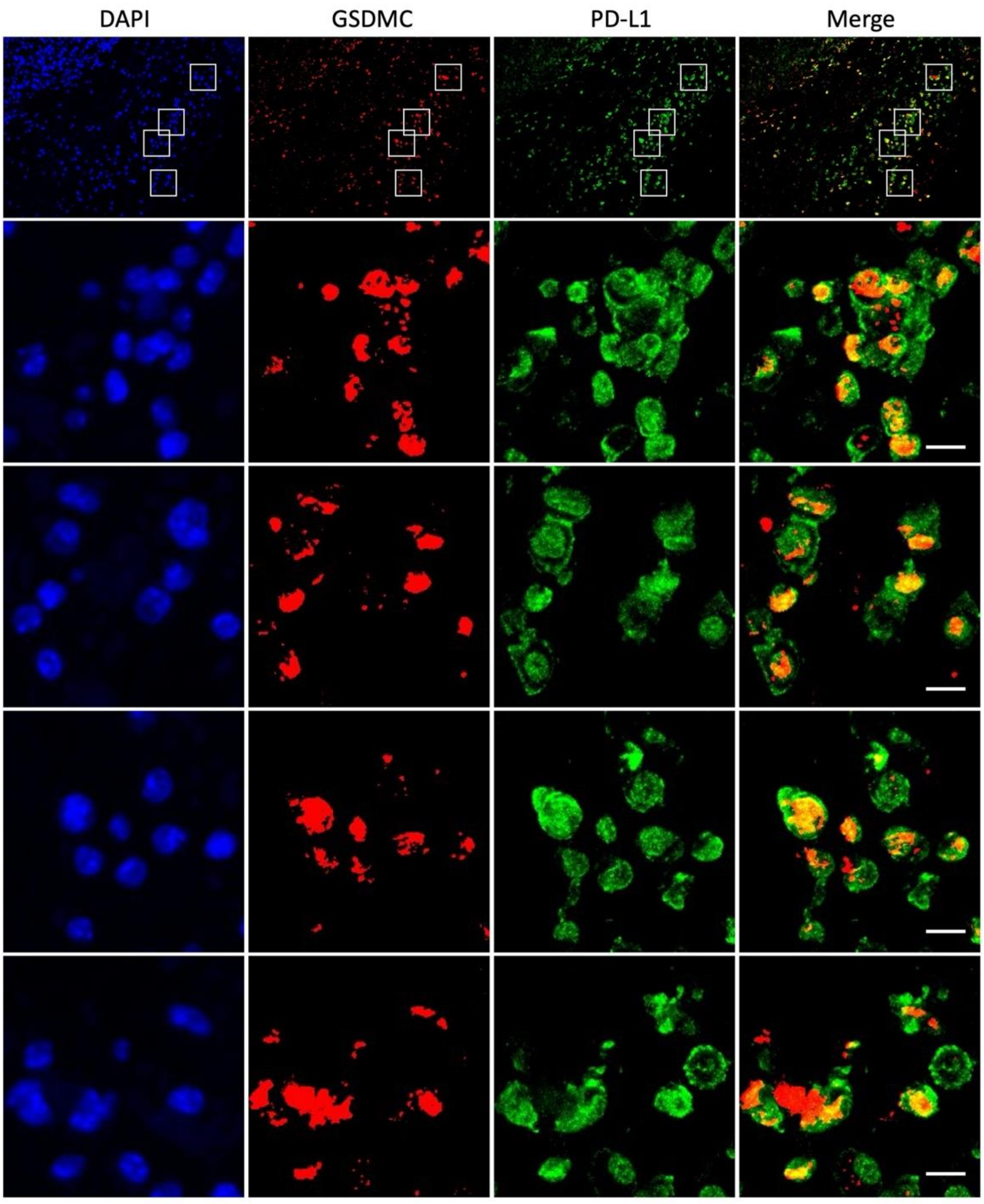
DAPI
GSDMC
PD-L1
Merge

L

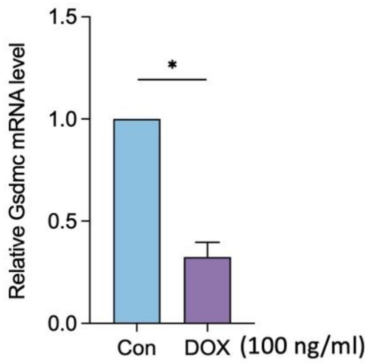

Figure S4 – GSDMC promotes immune evasion in PDAC. (**A**) Fold change in the expression of *Gsdmc*, immune evasion-related genes, and stemness-related genes in primary murine KPC cultures (CHX2000) following knockdown with two distinct sh*Gsdmc* variants. The dotted line represents the expression levels for sh*NC* (n=3 independent samples). (**B**) Proliferation of murine KPC cultures following knockdown of *Gsdmc* compared to control (sh*NC*) (n=3 independent samples). (**C**) Confluency of murine KPC cells (CHX2000) following knockdown of *Gsdmc* using two different sh*Gsdmc* variants or control sh*NC*, co-cultured with immortalized Bone Marrow-Derived Macrophages (iBMDM). Representative photographs (**left panel**) and quantification (**right panel**) (n=3 independent samples). (**D**) Confluency of murine sh*NC* and sh*Gsdmc* CHX2000 cells with treatment of CD47 antibody (10 µg/mL) or isotype IgG control, co-cultured with iBMDM cells. Representative images (**left panel**) and quantification (**right panel**) (n=3 independent samples, two-tailed t-test). (**E**) Representative H&E and IHC staining for the expression of GSDMC protein and the proliferation marker Ki67 (**left panels**). Quantification of staining results (**right panels**) (n=50 cells analyzed for GSDMC intensity from 3 independent samples and n=3 independent samples for Ki67 expression using one-way ANOVA with Games-Howell post-hoc test). **(F**) Representative H&E staining of lung sections to detect metastases in mice injected orthotopically with KPC cells following knockdown of Gsdmc using two sh*Gsdmc* variants or control (sh*NC*) (**left panel**). Quantification of metastatic lesions (**right panel**) (n=3 independent samples, one-way ANOVA analysis with Tukey's post-hoc test). (**G**) Liver metastases developed in murine metastasis PDAC model after intrasplenic injection of murine KPC cells (CHX2000) with knockdown of Gsdmc (sh*Gsdmc*) or control (sh*NC*). Representative photographs of macroscopic liver metastases (**left**) and assessment of liver metastases (**right**). N=10 for both groups. (**H**) Representative IHC staining for the T cell markers CD4 and CD8, the M2 macrophage marker CD163, and the M1 macrophage marker CD86 (n=3 independent samples). (**I**) Consecutive human PDAC TMA sections (sample size: n=50) were stained for GSDMC, CD163, and CD8 respectively. Representative IHC images of GSDMC-low and GSDMC-high samples, with CD163 and CD8 at corresponding sample area are shown (**upper panel**). Correlations of relative GSDMC intensity with

relative $CD163^+$ cell count or $CD8^+$ cell count were plotted. Pearson r and p-value to indicate correlation are shown (**lower panel**). (**J**) Schematic illustration of the treatment schedule for PD-1 inhibition in mice injected orthotopically with KPC cells following knockdown of *Gsdmc* using sh*Gsdmc* or control (sh*NC*) (**left**). Survival analysis (**right**); n=19-21, Log-rank test. (**K**) Co-localization of GSDMC and its downstream target PD-L1 in murine KPC-derived PDAC tumor models (CHX2000). (**L**) Fold change for the expression of *Gsdmc* following treatment with doxycycline (DOX, 100 ng/ml) or vehicle (Con) in murine *TetOn-shGsdmc* PDAC cells (n=3 independent samples). * p<0.05, ** p<0.01, *** p<0.001, and **** p<0.0001; Mann-Whitney U test, two-tailed, unless otherwise stated.

Figure S5 – Nuclear GSDMC transcriptionally regulates PDAC aggressiveness

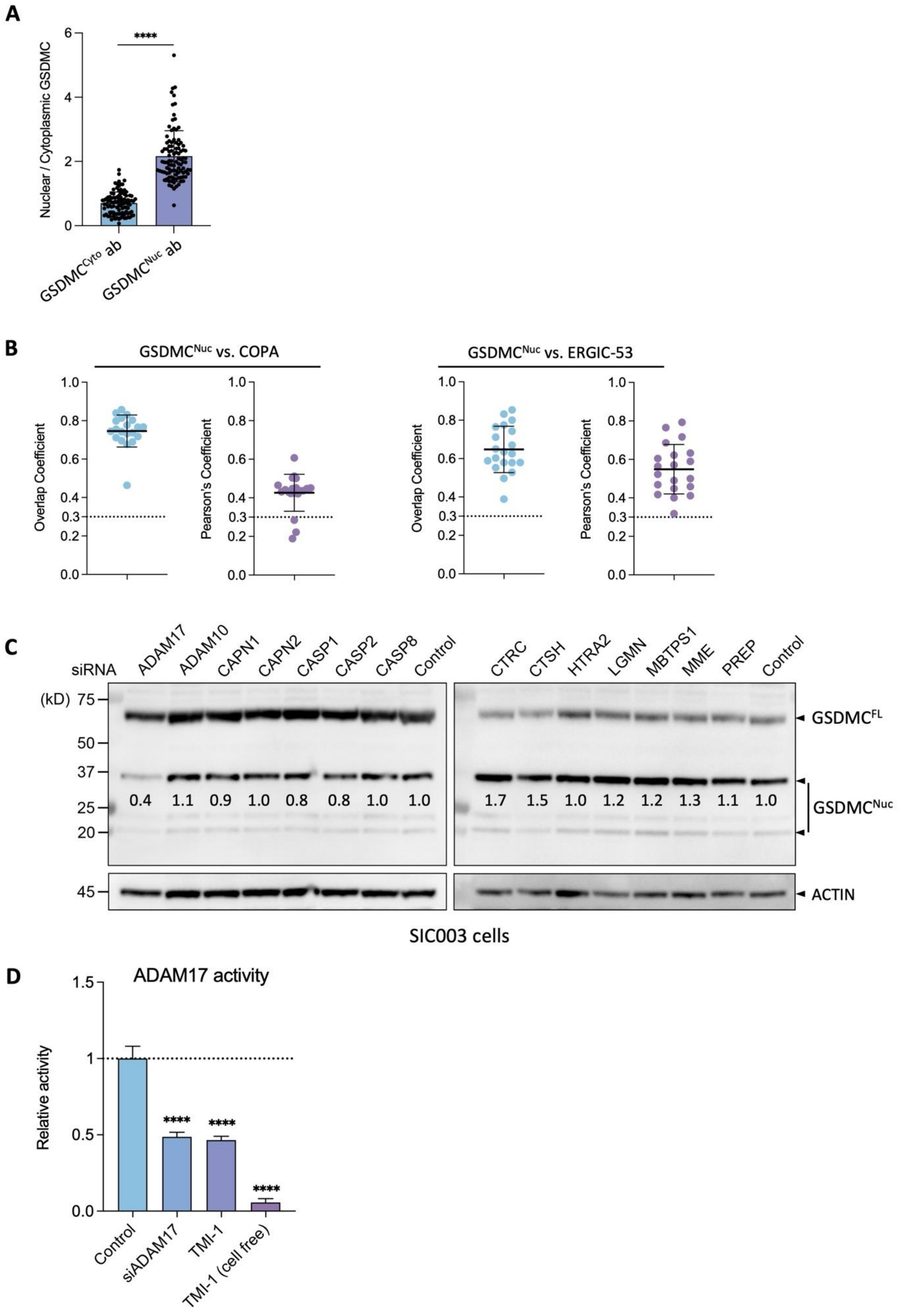

E

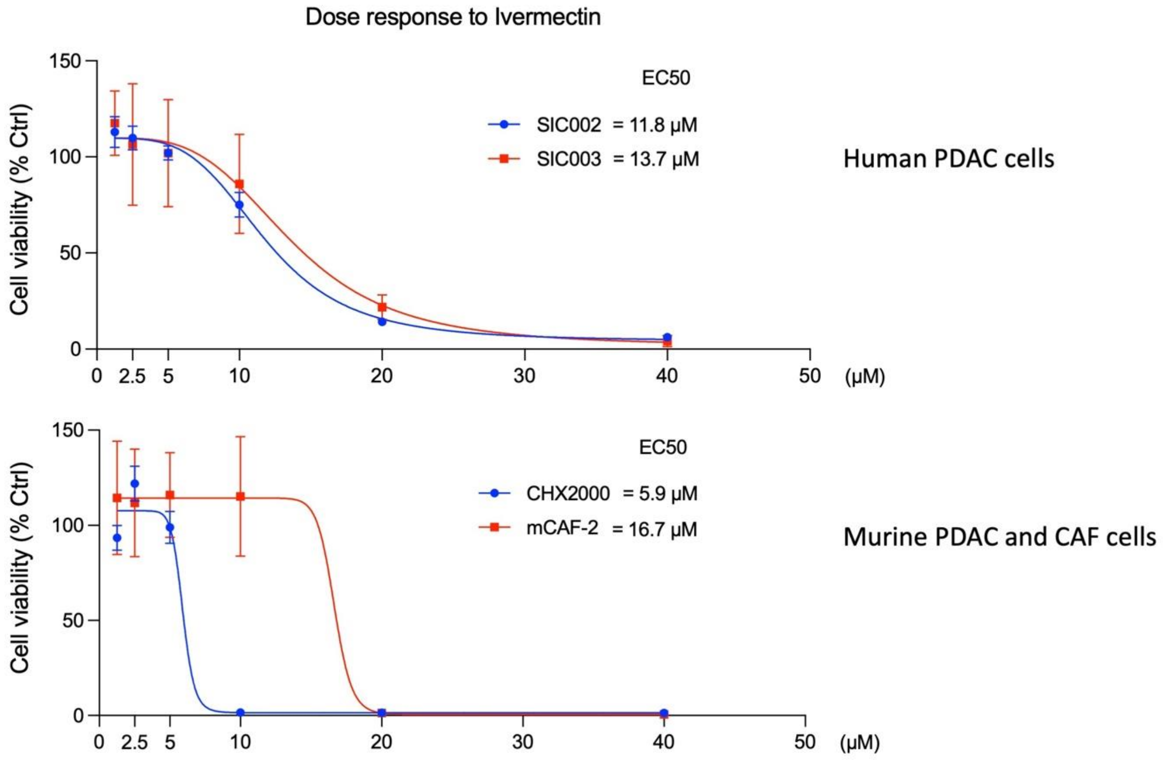

Dose response to Ivermectin
EC50
SIC002 = 11.8 µM
SIC003 = 13.7 µM
Human PDAC cells
Cell viability (% Ctrl)
150
100
50
0
0
2.5
5
10
20
30
40
50
(µM)
EC50
CHX2000 = 5.9 µM
mCAF-2 = 16.7 µM
Murine PDAC and CAF cells
Cell viability (% Ctrl)
150
100
50
0
0
2.5
5
10
20
30
40
50
(µM)

F

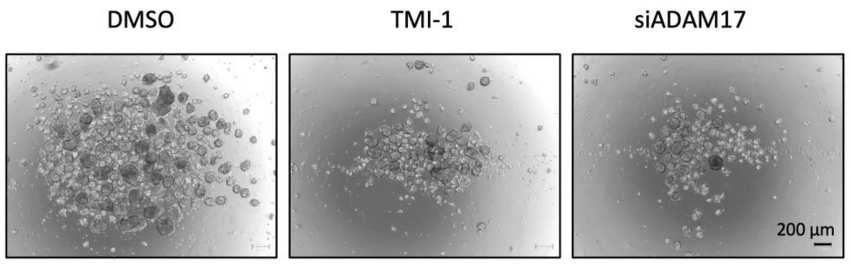

DMSO
TMI-1
siADAM17
200 µm

G

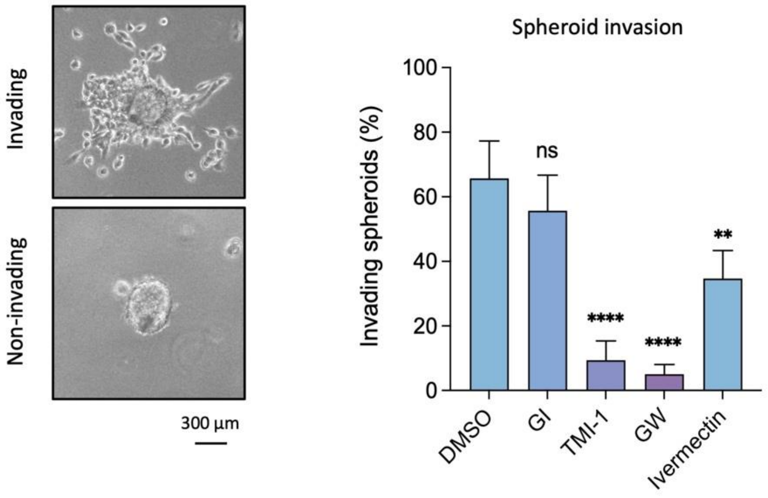

Invading
Non-invading
300 µm
Spheroid invasion
Invading spheroids (%)
100
80
60
40
20
0
ns
****
****
**
DMSO
GI
TMI-1
GW
Ivermectin

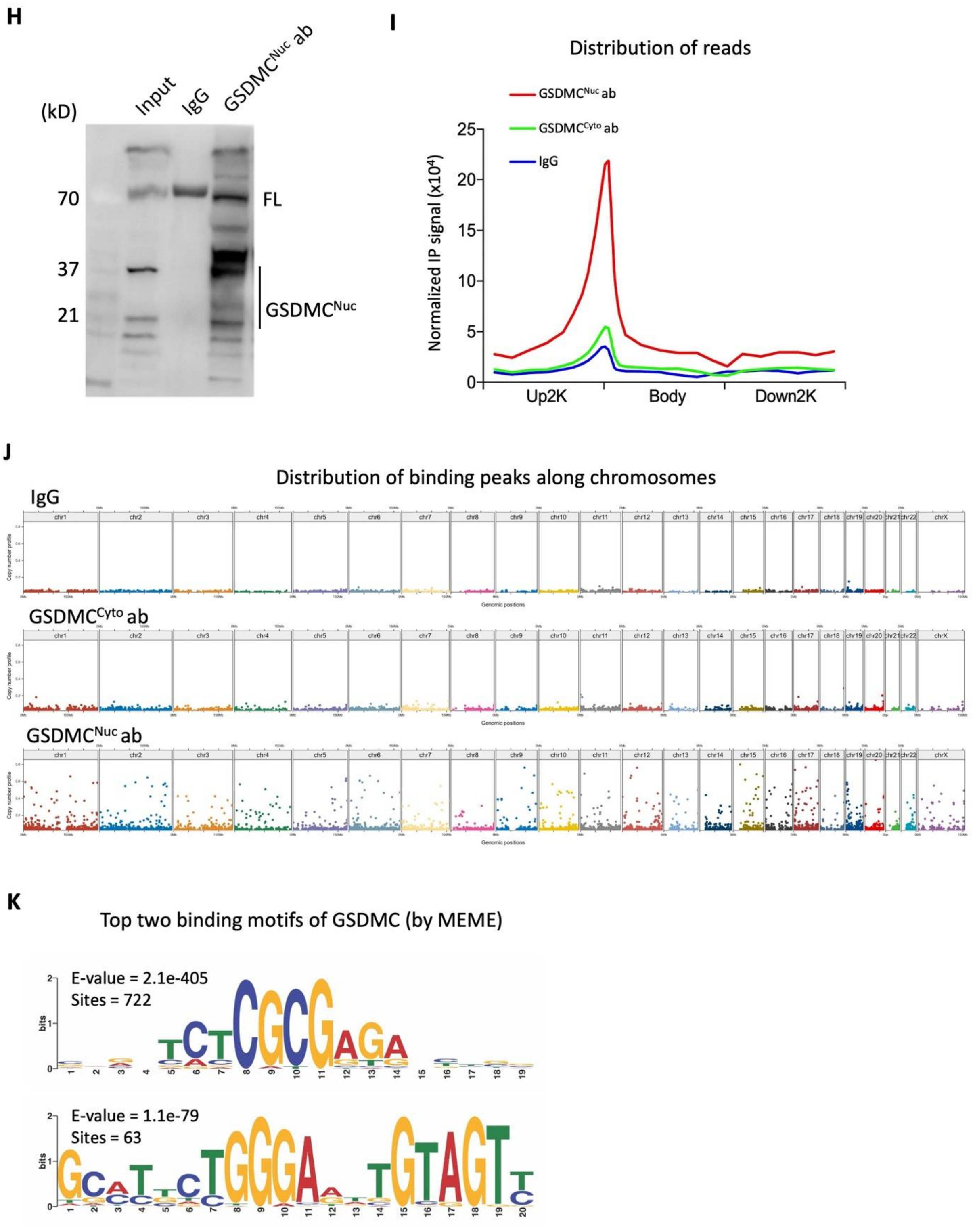

Figure S5 – Nuclear GSDMC transcriptionally regulates PDAC aggressiveness. (**A**) Two distinct antibodies were used for these experiments. One antibody (ABClonal, #A14550, Ab^{Cyto}) primarily identified cytosolic GSDMC, therefore referred to as the GSDMC^{Cyto}, representing full-length GSDMC. A second antibody (ServiceBio, #GB111623, Ab^{Nuc}) preferentially detected nuclear GSDMC, therefore referred to as the GSDMC^{Nuc}. The nuclear-to-cytoplasmic ratio of GSDMC was determined by scoring the

staining intensity using the Ab^{Nuc} compared to Ab^{Cyto}. A total of 100 cells per group were analyzed. (**B**) Colocalization analysis of GSDMC with COPA or ERGIC-53 was conducted using the ImageJ plugin JACoP. Pearson's Coefficient and Overlap Coefficient were calculated from confocal images (n=20). A value of r>0.3 denotes correlated expression of the two markers. (**C**) Western blot analysis was conducted to detect the cleavage of $GSDMC^{Cyto}$ into $GSDMC^{Nuc}$ in human PDAC cells. The cells were treated with siRNA against the following enzymes: *ADAM17*, *ADAM10*, *CALPAIN-1 (CAPN1)*, *CALPAIN-2 (CAPN2)*, *CASPASE-1 (CASP1)*, *CASPASE-2 (CASP2)*, *CASPASE-8 (CASP8)*, *CHYMOTRYPSIN C (CTRC)*, *CATHEPSIN H (CTSH)*, High-Temperature Requirement Protein A2 (*HTRA2*), *LEGUMAIN (LGMN)*, Membrane Bound Transcription Factor Peptidase, Site 1 (*MBTPS1*), Membrane Metalloendopeptidase (*MME*), Prolyl Endopeptidase (*PREP*), and Negative Controls (si*NC*). Quantification was performed as the ratio of $GSDMC^{Cyto}$ versus $GSDMC^{Nuc}$. (**D**) Activity of ADAM17 in PDAC cells following treatment with control, TMI-1 or siADAM17 for 48 hours. For the cell-free group, TMI-1 was mixed directly with the active ADAM17 enzyme (n=3 independent samples, one-way ANOVA analysis with Tukey's post-hoc test). (**E**) Viability test to determine the EC50 for ivermectin *in vitro* for human PDAC cultures (**upper panel**) and murine KPC models (CHX2000) and murine cancer-associated fibroblasts (CAF: here mCAF-2) (**lower panel**) (n=3 independent samples). (**F**) Sphere formation capacity of human PDAC cells following treatment with ivermectin, the ADAM17 inhibitor TMI-1, and si*ADAM17* compared to control (DMSO). Representative images are shown. (**G**) Spheroid invasion assays in the presence of the ADAM17 inhibitor TMI-1, the ADAM10 inhibitor GI254023X (GI), the ADAM10/17 inhibitor GW280264X (GW), and ivermectin in human PDAC cultures. Representative images (**left panel**), quantification (**right panel**). N=3 independent samples, one-way ANOVA analysis with Tukey's post-hoc test. (**H**) Western blot analysis was conducted to detect $GSDMC^{FL}$ and $GSDMC^{Nuc}$ in PDAC cultures after immunoprecipitation with the Ab^{Nuc} antibody or isotype IgG. The Ab^{Nuc} recognized the full-length GSDMC (GSDMC-FL) and $GSDMC^{Nuc}$ fragments. (**I**) Distribution of ChIP-seq reads represented by the Normal IP signal along genomic regions, including Up2K (upstream 2 kilobases), Body (gene body), and Down2K (downstream 2 kilobases). The distribution patterns for antibodies directed against different GSDMC fragments are displayed. The **red** line represents the $GSDMC^{Nuc}$ antibody, whereas the **green** line signifies the $GSDMC^{Cyto}$ antibody. IgG control antibody is shown in **blue**. (**J**) Chromosomal binding distribution as assessed by ChIP-seq using pull-down with the $GSDMC^{Nuc}$ antibody ($GSDMC^{Nuc}$: **lower panel**) or the $GSDMC^{Cyto}$ antibody ($GSDMC^{Cyto}$: **middle panel**). IgG antibody was used as a negative control (**upper panel**). (**K**) The top two binding motifs of GSDMC, identified using MEME analysis for the detection of conserved motifs, are represented as nucleotide sequences, with the motif sequences highlighted in uppercase letters. (**L**) Enriched Gene Ontology (GO) and Kyoto Encyclopedia of

Genes and Genomes (KEGG) pathways (**shown in M**) in SIC003 PDAC primary cultures following immunoprecipitation (IP) using the $GSDMC^{Nuc}$ antibody. IgG antibody was used as a control. (**N**) Overexpression of mutant human GSDMC was performed to assess target gene expression. Wildtype (WT) and three plasmids with deletions of specific amino acid regions (310-331, 392-413, 456-477) were constructed and transfected into SiC002 cells for 48 hours. The expression levels of the target genes CD24, CD44, CD47, and CD274 were measured (n=3 independent samples). * $p<0.05$, ** $p<0.01$, and **** $p<0.0001$; Mann Whitney test, two-tailed, unless specified otherwise.

Figure S6 – GSDMC inhibition unleashes CXCL9-mediated influx of T cells

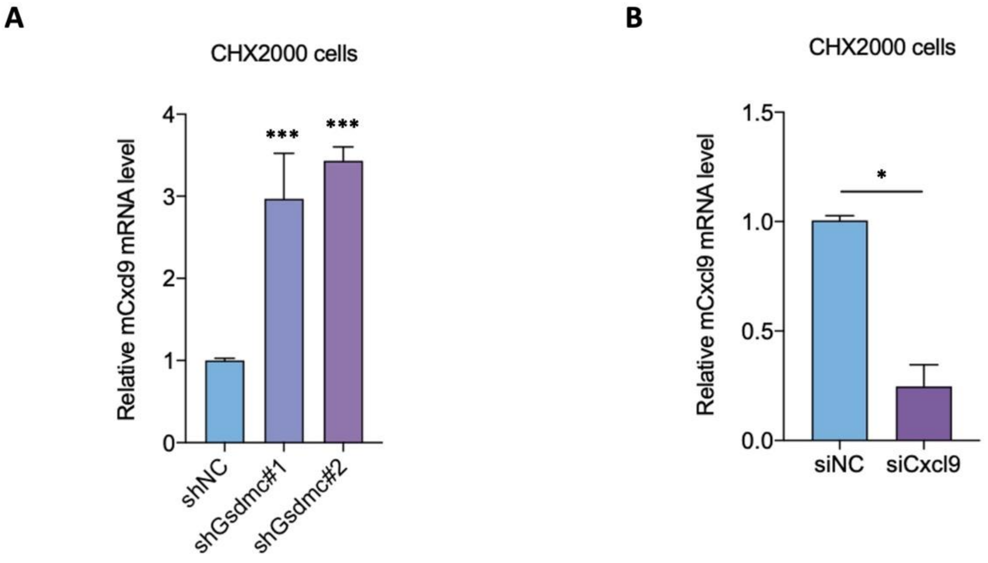

Figure S6 – GSDMC inhibition unleashes CXCL9-mediated influx of T cells. (**A**) Fold change in mRNA expression for *Cxcl9* in murine PDAC cells in response to transduction with two different sh*Gsdmc* variants compared to control sh*NC* (n=3 independent samples, one-way ANOVA analysis with Tukey's post-hoc test). (**B**) Fold change of *Cxcl9* expression in murine primary PDAC cell cultures following pre-treatment with si*Cxcl9* (n=3 independent samples, two-tailed Mann-Whitney U test). * p<0.05, and *** p<0.001.

Figure S7 – Identification of upstream modulators of GSDMC expression

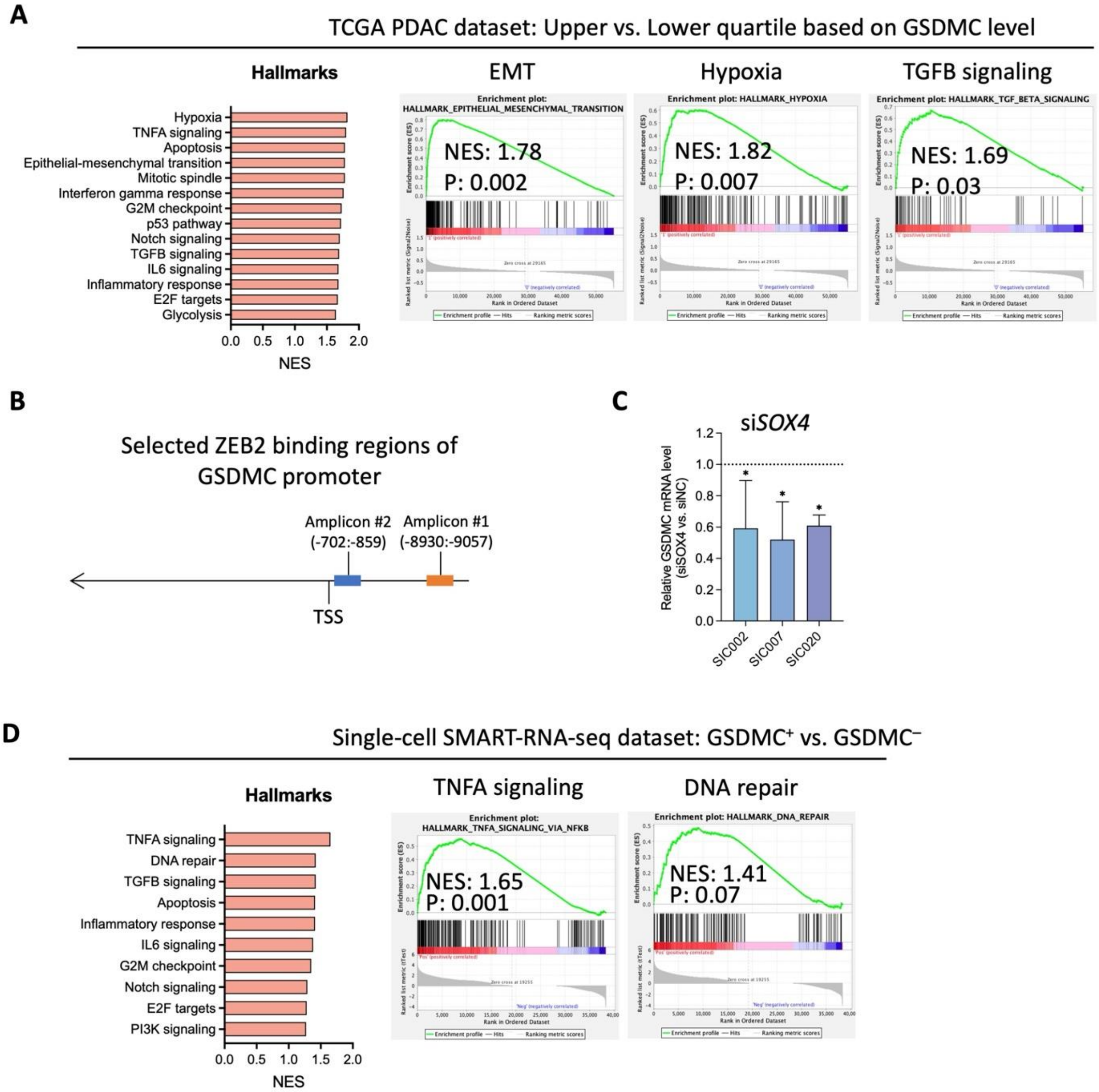

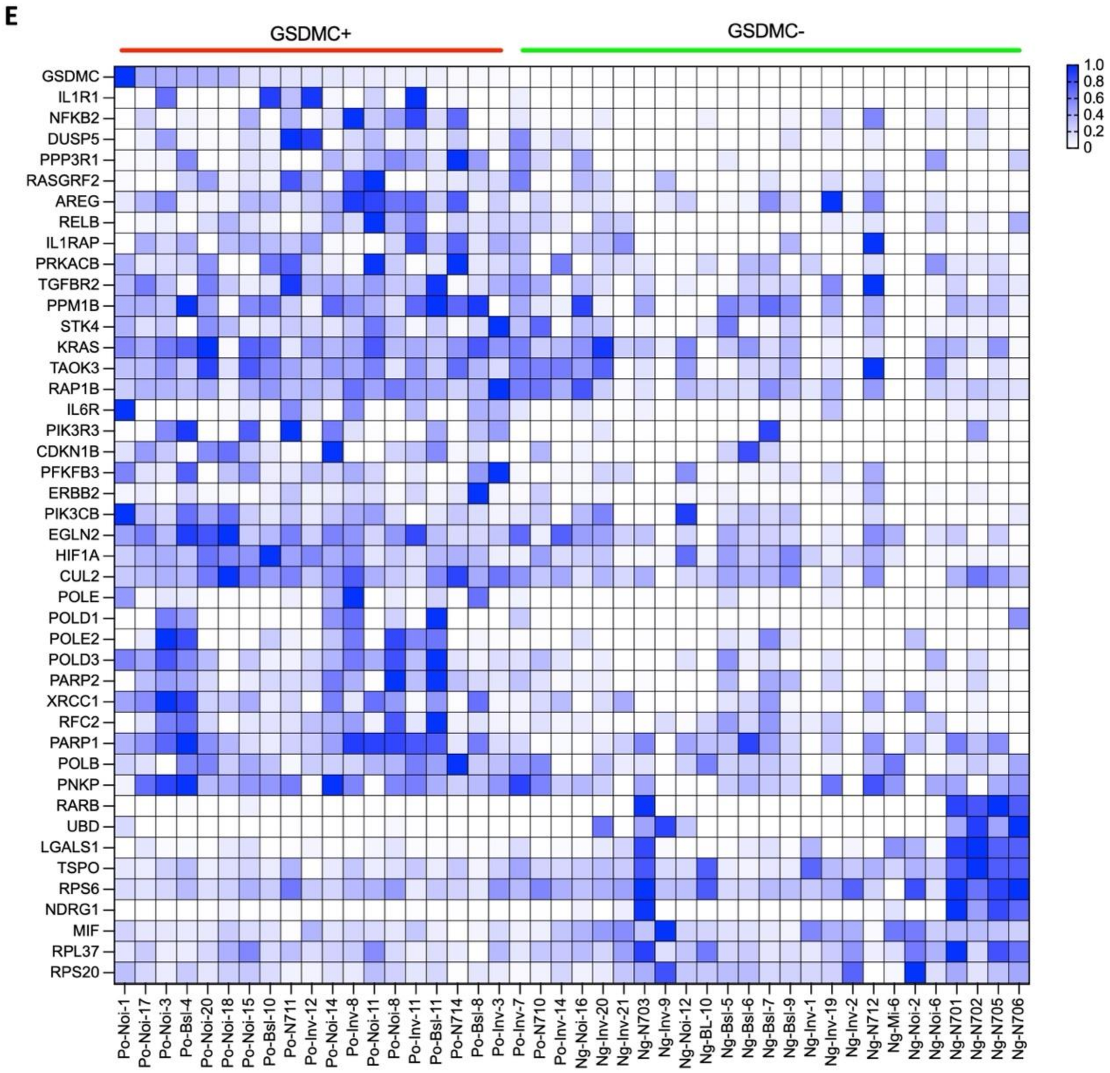
E
GSDMC+
GSDMC-
1.0
0.8
0.6
0.4
0.2
0
GSDMC
IL1R1
NFKB2
DUSP5
PPP3R1
RASGRF2
AREG
RELB
IL1RAP
PRKACB
TGFBR2
PPM1B
STK4
KRAS
TAOK3
RAP1B
IL6R
PIK3R3
CDKN1B
PFKFB3
ERBB2
PIK3CB
EGLN2
HIF1A
CUL2
POLE
POLD1
POLE2
POLD3
PARP2
XRCC1
RFC2
PARP1
POLB
PNKP
RARB
UBD
LGALS1
TSPO
RPS6
NDRG1
MIF
RPL37
RPS20
Po-Noi-1
Po-Noi-17
Po-Noi-3
Po-Bsl-4
Po-Noi-20
Po-Noi-18
Po-Noi-15
Po-Bsl-10
Po-N711
Po-Inv-12
Po-Noi-14
Po-Inv-8
Po-Noi-11
Po-Noi-8
Po-Inv-11
Po-Bsl-11
Po-N714
Po-Bsl-8
Po-Inv-3
Po-Inv-7
Po-N710
Po-Inv-14
Ng-Noi-16
Ng-Inv-20
Ng-Inv-21
Ng-N703
Ng-Inv-9
Ng-Noi-12
Ng-BL-10
Ng-Bsl-5
Ng-Bsl-6
Ng-Bsl-7
Ng-Bsl-9
Ng-Inv-1
Ng-Inv-19
Ng-Inv-2
Ng-N712
Ng-Mi-6
Ng-Noi-2
Ng-Noi-6
Ng-N701
Ng-N702
Ng-N705
Ng-N706

F

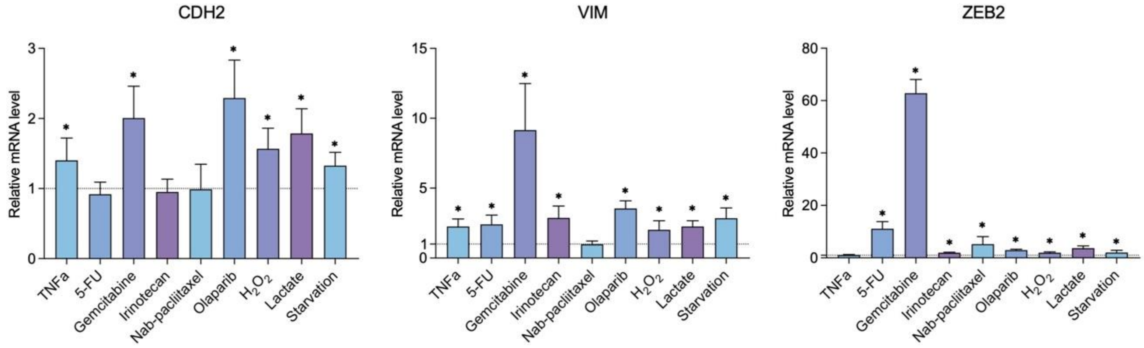
CDH2
VIM
ZEB2
Relative mRNA level
TNFa
5-FU
Gemcitabine
Irinotecan
Nab-paclitaxel
Olaparib
H2O2
Lactate
Starvation

G

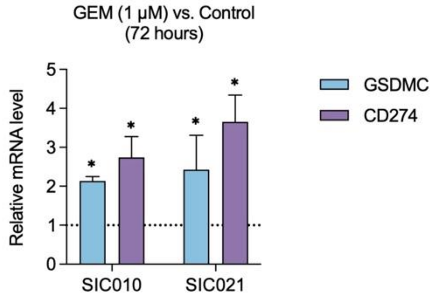
GEM (1 μM) vs. Control
(72 hours)
Relative mRNA level
GSDMC
CD274
SIC010
SIC021

H

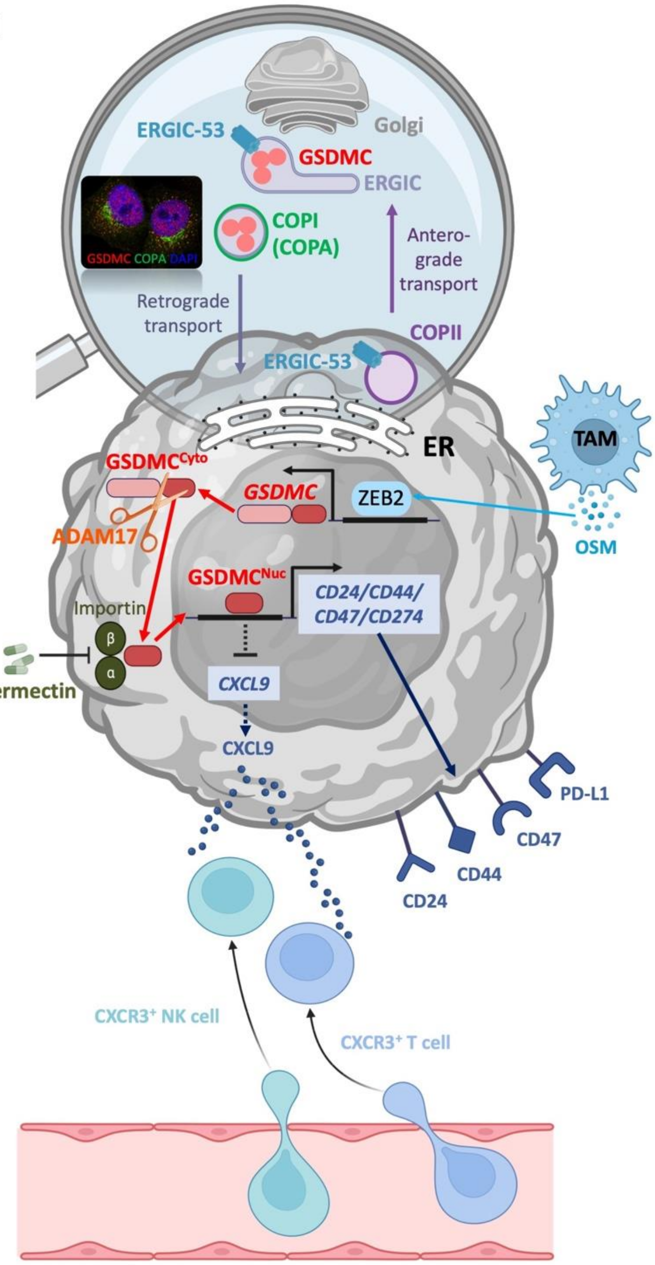
Golgi
ERGIC-53
GSDMC
ERGIC
COPI
(COPA)
GSDMC COPA DAPI
Antero-
grade
transport
Retrograde
transport
COPII
ERGIC-53
ER
TAM
GSDMC^Cyto
GSDMC
ZEB2
OSM
ADAM17
GSDMC^Nuc
CD24/CD44/
CD47/CD274
Importin
β
α
Ivermectin
CXCL9
CXCL9
PD-L1
CD47
CD44
CD24
CXCR3⁺ NK cell
CXCR3⁺ T cell

Figure S7 – Identification of upstream modulators of GSDMC expression*. (**A**) Hallmark enrichment plots generated using TCGA PDAC datasets, dividing the samples into upper and lower quartiles based on *GSDMC* expression levels. The **left** panel displays the normalized enrichment scores (NES) for various hallmark pathways, indicating their response to GSDMC levels. The **right** panel shows enrichment plots specifically for pathways related to epithelial-mesenchymal transition (EMT), hypoxia, and TGFβ signaling. The normalized enrichment scores (NES) and corresponding p-values are provided to assess the significance of enrichment. (**B**) Schematic illustration for selected ZEB2 binding regions within the promoter of *GSDMC*. Two amplicons located at the transcription start site (TSS) are highlighted, indicating specific regions of ZEB2 binding. (**C**) Fold change of *GSDMC* expression in three different human primary PDAC cell cultures following pre-treatment with si*SOX4*. The dotted line represents control treatment with si*NC* (n=3 independent samples). (**D**) Hallmark enrichment plots generated using single-cell SMART-RNA-seq datasets, dividing the samples into $GSDMC^{+}$ and $GSDMC^{-}$ expression levels. The **left** panel displays the normalized enrichment scores (NES) for various hallmark pathways, indicating their correlation to *GSDMC* expression levels. The **middle and right** panel show enrichment plots specifically for pathways related to TNFα signaling and DNA repair. The normalized enrichment scores (NES) and corresponding p-values are provided to assess the significance of enrichment. (**E**) Heatmap analysis for $GSDMC^{+}$ and $GSDMC^{-}$ PDAC cells, focusing on the expression of *GSDMC* and its related genes. The heatmap provides an overview of the expression patterns and potential correlations between these genes. (**F**) Expression of the EMT marker genes *CDH2*, *VIM*, and *ZEB2* in human primary PDAC cells after treatment with various stressors for 48 hours. Cells treated with DMSO are used for normalization (n=3 independent samples). (**G**) Expression of *GSDMC* and *CD274* in the gemcitabine-resistant primary PDAC cells SIC010 and SIC021 after treatment with low-dose gemcitabine (1 µM) for 72 hours (n=3 independent samples). (**H**) The schematic illustrates the subcellular compartments and their specific markers involved in intracellular trafficking. COPA, a subunit of the coat protein complex I (COPI), facilitates retrograde intracellular trafficking of cargo proteins from the Golgi to the ER, while COPII is responsible for anterograde transport from the ER to the Golgi. The ERGIC, or ER-Golgi intermediate compartment, plays a role in protein trafficking. ERGIC-53, an ER export receptor, shuttles cargo between the ER and the ERGIC. The magnifying glass reveals the intracellular trafficking of GSDMC. The schematic depicts the co-localization of GSDMC with the retrograde Golgi to ER marker COPA and the ERGIC-53 marker, which mediates anterograde cargo transport between the ER and ERGIC. This suggests a spatial association of GSDMC with key components of intracellular trafficking pathways. The schematic also portrays the molecular mechanism underlying the activation of the EMT-related transcription factor ZEB2 by cytokines released by tumor-associated macrophages (TAMs), including Oncostatin M (OSM), and others. This

activation leads to the transcriptional initiation of GSDMC. Subsequently, GSDMC is cleaved by ADAM17 releasing $GSDMC^{Nuc}$ fragments. Importantly, $GSDMC^{Nuc}$ is transported into the nucleus, which can be inhibited by the importin α/β inhibitor ivermectin. Once in the nucleus, transcription is initiated for genes such as *CD24, CD44, CD47*, and *CD274 (PD-L1*), resulting in the expression of corresponding proteins on the cell surface, and promotion of stemness, invasion, metastasis, and immune evasion. Moreover, the expression of the chemokine CXCL9, which recruits $CXCR3^+$ anti-tumor immune cells, such as $CD8^+$ cytotoxic T cells and NK cells, is inhibited,. The image for our working model was created with BioRender.com. * $p<0.05$; Mann-Whitney U test, two-tailed.

Figure S8 – Therapeutic targeting of GSDMC in preclinical PDAC models

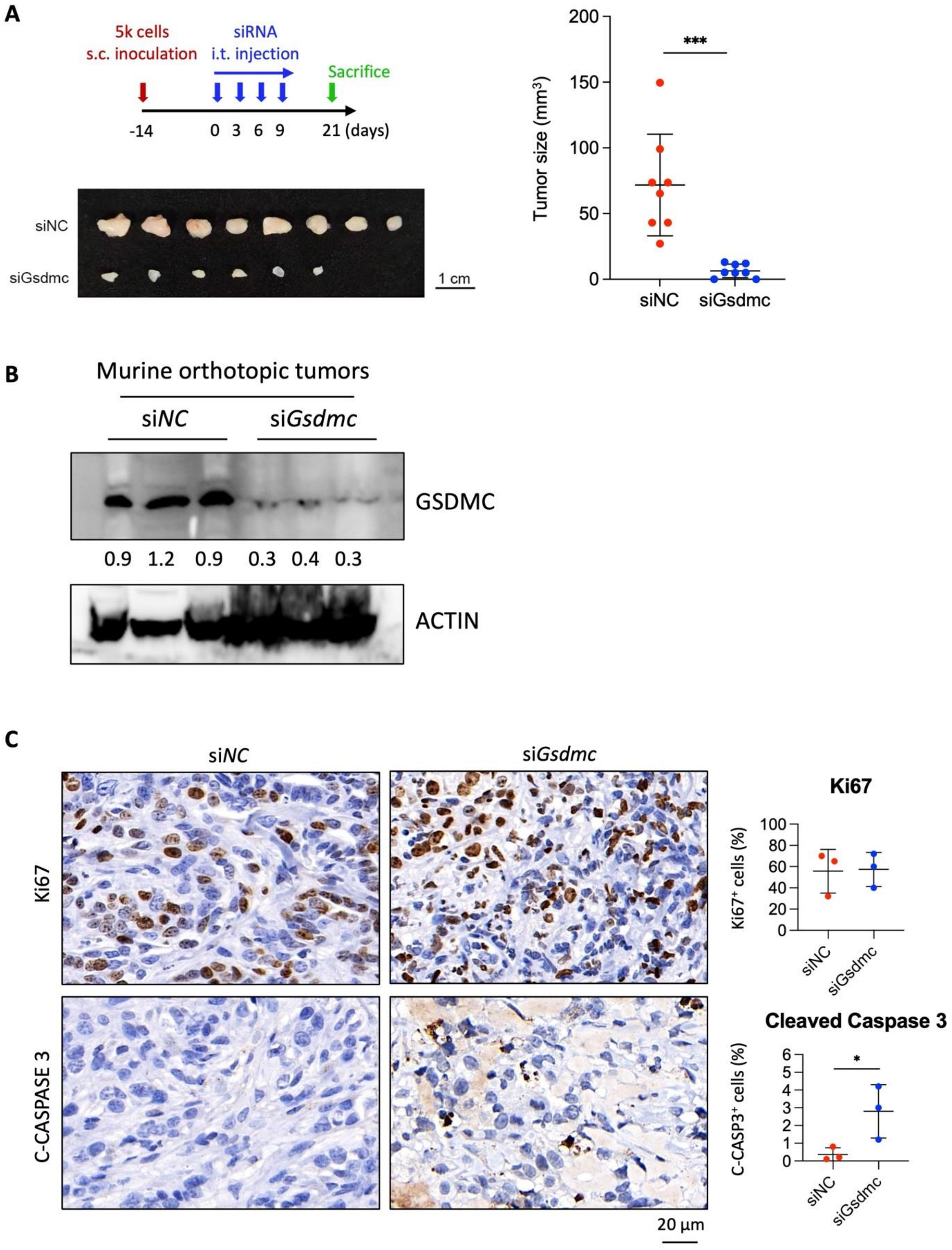

D

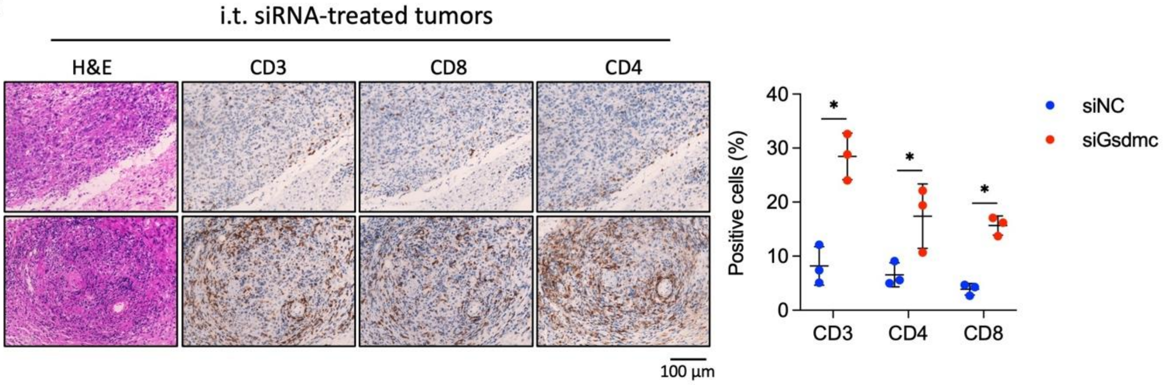

E

Immunofluorescence of siRNA-treated tumors

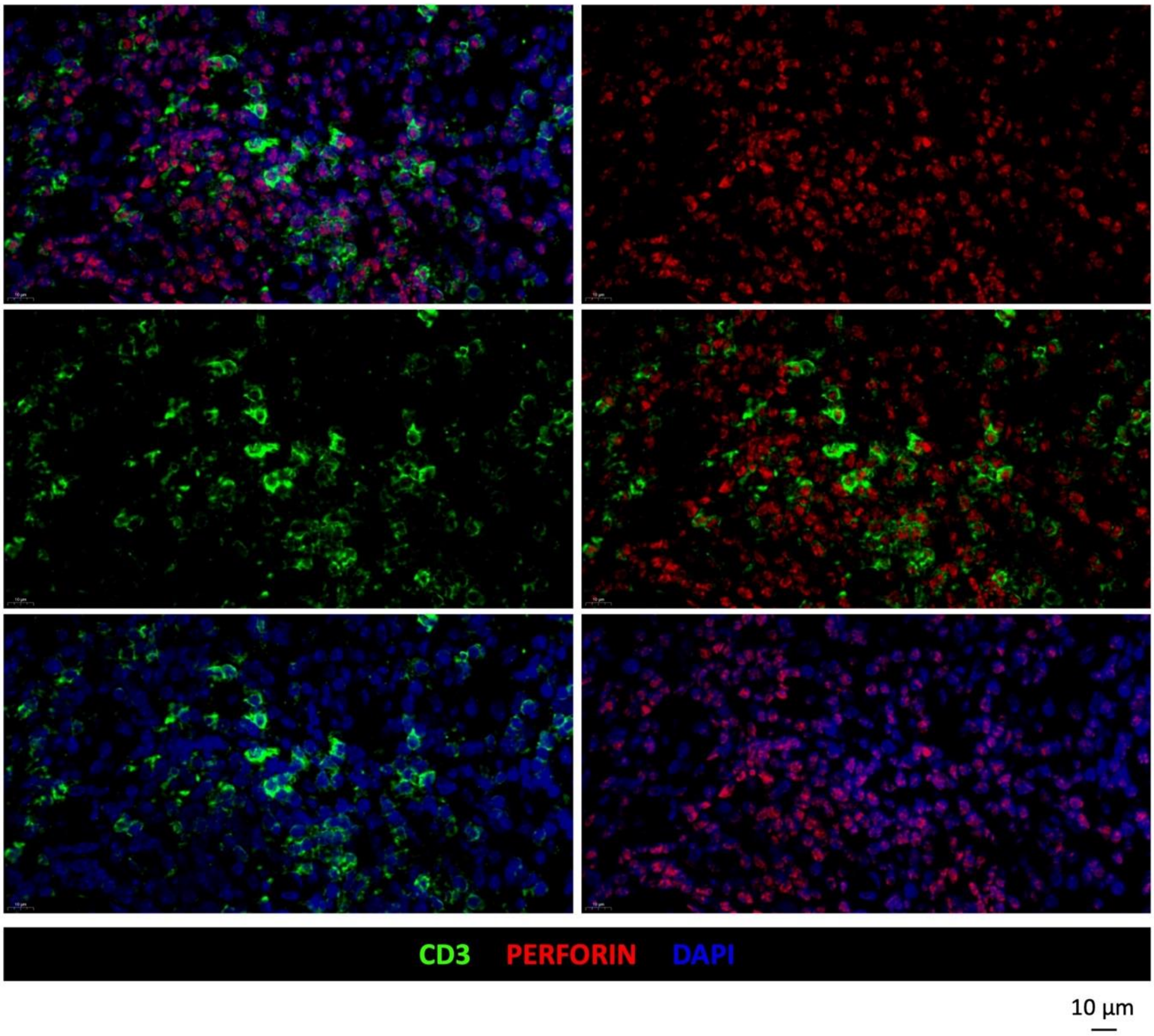

F

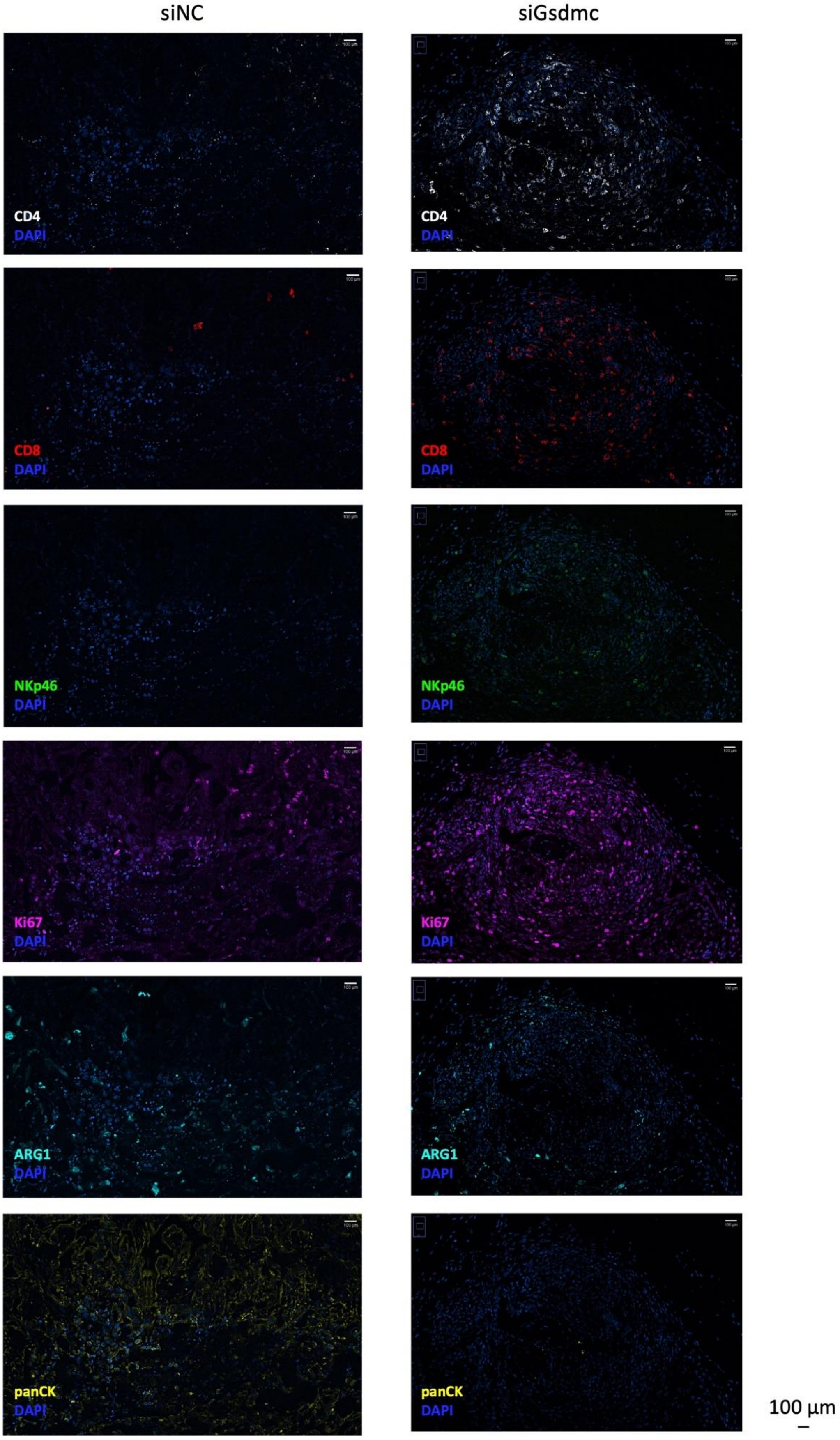
siNC
siGsdmc
CD4
DAPI
CD8
DAPI
NKp46
DAPI
Ki67
DAPI
ARG1
DAPI
panCK
DAPI
100 µm

G

siNC

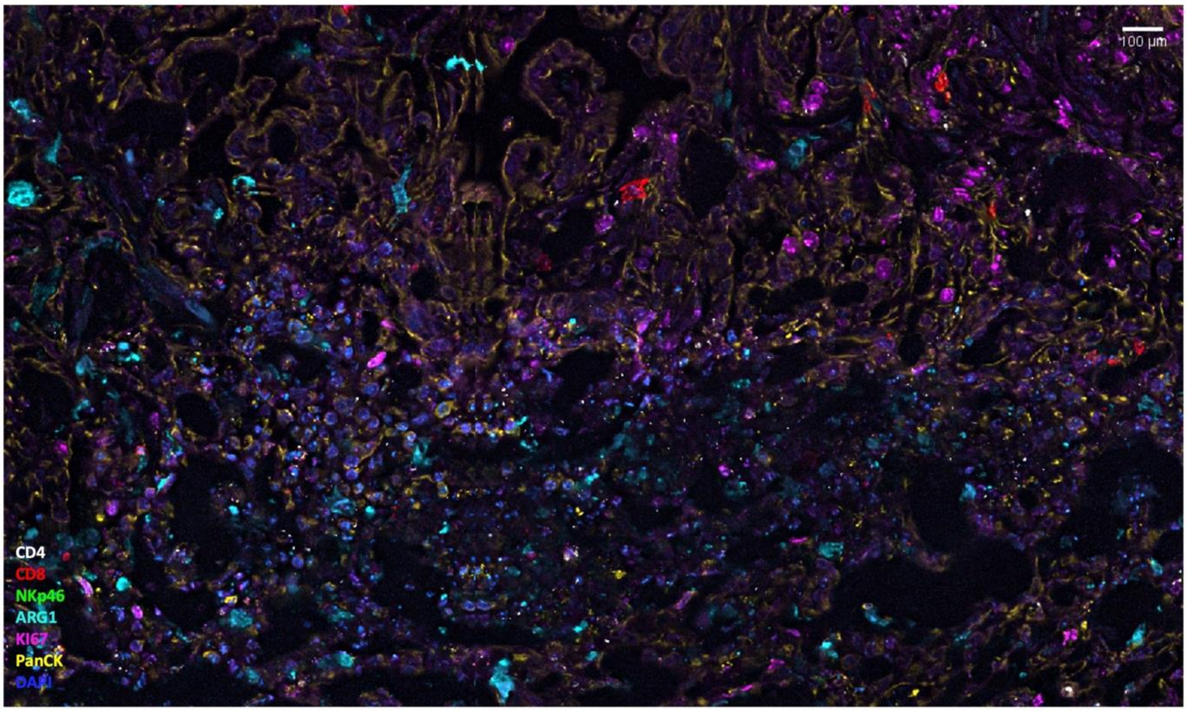

siGsdmc

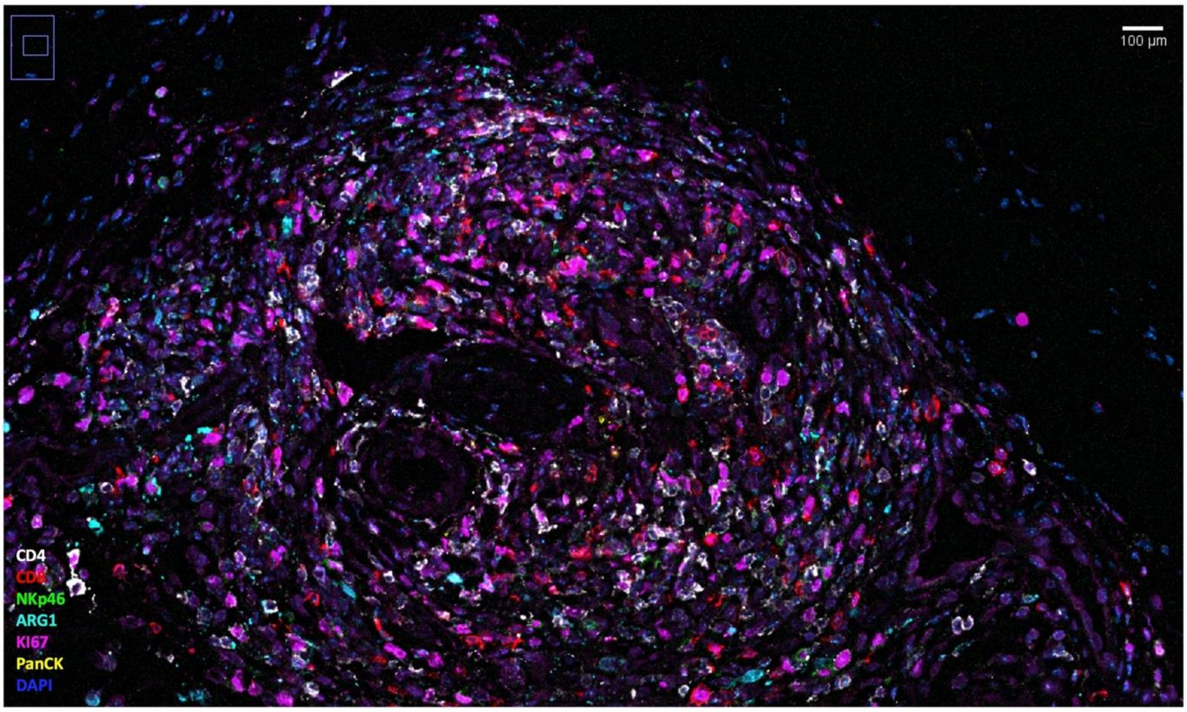

100 µm

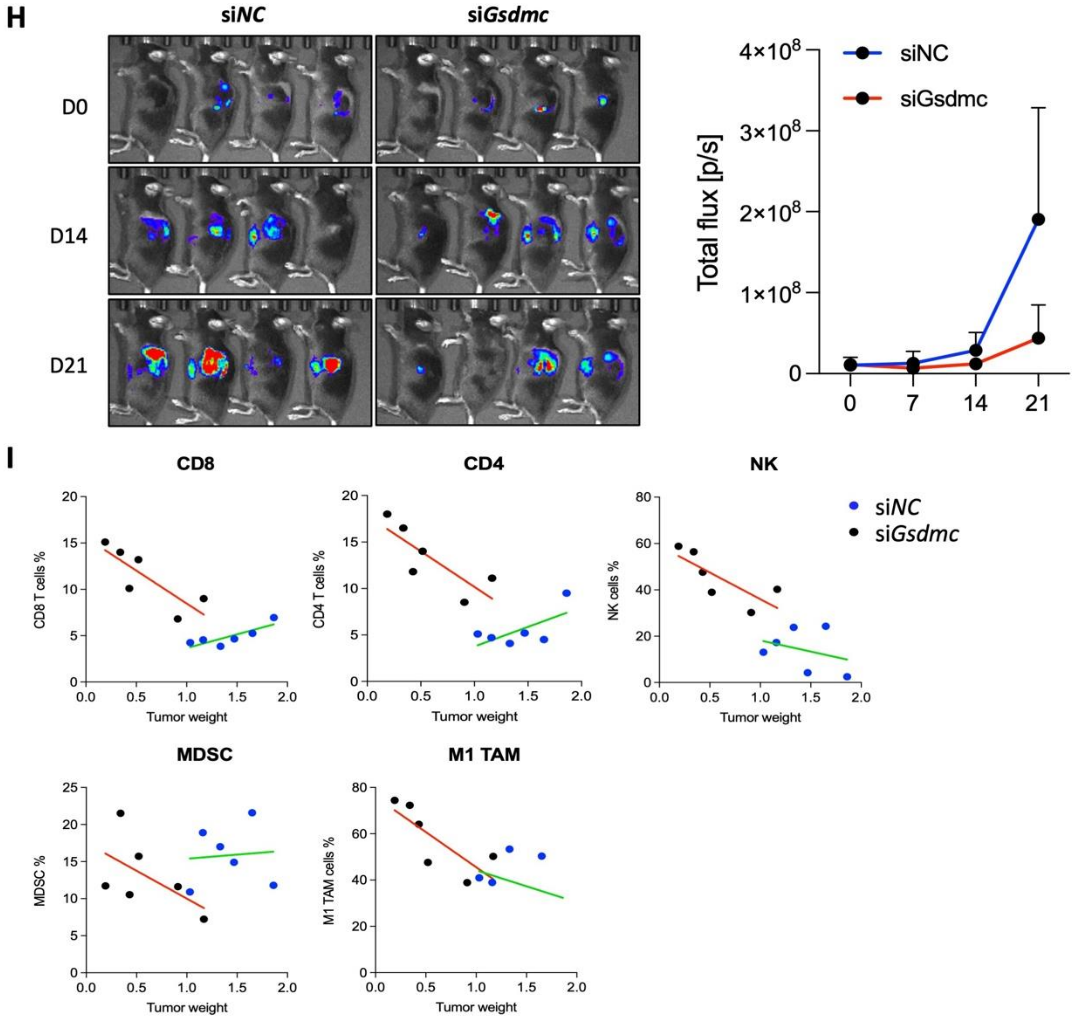

Figure S8 – Therapeutic targeting of GSDMC in preclinical PDAC models. (**A**) Schematic of the experimental design involving subcutaneous inoculation of KPC cells (CHX2000), followed by intratumoral siRNA injections starting after 14 days (**upper panel**). Macroscopic images of the tumors following intratumoral injections of si*Gsdmc* or control si*NC* (**lower panel).** Quantification of tumor sizes (**right panel);** n=8. (**B**) Western blot analysis to assess the GSDMC knockdown following treatment with si*Gsdmc* versus si*NC* in murine orthotopic tumors (CHX2000). ACTIN was used as a loading control. The ratio of GSDMC to β-ACTIN expression is indicated. (**C**) Analysis for proliferation (Ki67, **upper panel**) and apoptosis (cleaved CASPASE 3; c-CASP-3, **lower panel**) in murine KPC tumors (CHX2000) with (si*Gsdmc*) or without (si*NC*) knockdown of Gsdmc. Representative images (**left panel)** and quantification (**right panel)** (n=3 independent samples). (**D**) H&E and IHC staining for CD3, CD8, and CD4. Representative images (**left panel)** and quantification of cell counts (**right panel)** (n=3 independent samples). (**E**) Immunofluorescence of CD3 and PERFORIN in si*Gsdmc*-treated murine tumors to demonstrate the presence of double-positive cytotoxic T cells. (**F**) Two-color display for CODEX multiplex imaging for tumors treated with si*NC* versus si*Gsdmc*. (**G**) Merge images. (**H**)

Representative IVIS images for luciferase^{+} KPC tumors following systemic delivery of si*Gsdmc* compared to si*NC* control (**left panel**). Quantification of the total flux as a surrogate for tumor size (**right panel**). (**I**) Correlation analysis for tumors of equal size treated with intratumoral injections of si*Gsdmc* versus control si*NC* as shown in Figure 8A. The relationship between CD8^{+} T cells, CD4^{+} T cells, NK cells, MDSCs, M1 TAM, and tumor weight is shown. * $p<0.05$ and *** $p<0.001$; Mann-Whitney U test, two-tailed.
